# Renal protective effects and mechanisms of tripterygium glycosides in animal models of diabetic nephropathy: a preclinical systematic review and meta-analysis

**DOI:** 10.3389/fphar.2026.1863463

**Published:** 2026-08-25

**Authors:** Mengdi He, Weiyi Feng, Chengchen Xu, Zhitian Cheng, Rensong Yue, Maoyi Yang, Zhipeng Hu

**Affiliations:** 1 Hospital of Chengdu University of Traditional Chinese Medicine, Chengdu, China; 2 School of Clinical Medicine, Chengdu University of Traditional Chinese Medicine, Chengdu, China; 3 School of Basic Medicine, Chengdu University of Traditional Chinese Medicine, Chengdu, China

**Keywords:** animal models, diabetic nephropathy, meta-analysis, protective mechanisms, tripterygium glycosides

## Abstract

**Background:**

Diabetic nephropathy (DN) is one of the most common microvascular complications of diabetes mellitus (DM), clinically characterized by progressive loss of renal function and/or persistent albuminuria. Tripterygium glycosides (TGs) have been used in the clinical treatment of DN. There are many animal experiments on TGs intervention in DN, but the evidence from these studies remains unclear.

**Objective:**

This study aims to summarize the renal protective effects and mechanisms of TGs in DN animal models.

**Methods:**

A comprehensive search of animal studies from the inception of 8 databases to May 2026 was conducted. The bias risk of the included studies was evaluated using SYRCLE’s risk of bias tool. The evidence certainty of the outcomes was assessed using the GRADE method. The meta-analysis was conducted using RevMan 5.4 and Stata 17.0 software.

**Results:**

A total of 55 studies involving 1,481 animals were included. Analysis showed that TGs significantly reduced Scr, BUN, 24h UP, 24h UA, 24h UMA, KW, KI, BG, TC, TG, MGA, and MGV levels while increasing ALB levels, but did not significantly affect ALT, AST, WBC, RBC, or PLT levels. Additionally, TGs modulated the expression of most markers related to oxidative stress, inflammation, renal fibrosis, podocyte injury, and autophagy. Subgroup analysis by intervention time suggested that longer intervention time (>8 weeks) increased ALT levels. Dose-effect-toxicity analysis indicated that medium doses (6–9 mg/kg/day) balanced efficacy and safety, while high doses (>9 mg/kg/day) elevated AST levels and reduced WBC levels. The methodological quality was moderate to low, and the evidence quality was moderate to very low.

**Conclusion:**

TGs have a favorable renal protective effect on DN animals, which can improve renal function, basic indicators, glucose and lipid metabolism, and renal tissue pathological changes. For safety reasons, the intervention time (≤8 weeks) and dose (6–9 mg/kg/day) should be strictly restricted to prevent potential hepatotoxicity and hematological toxicity. Its protective mechanisms mainly include anti-oxidative stress, relieving inflammation, inhibiting renal fibrosis, alleviating podocyte injury, and regulating autophagy. Given the limited methodological and evidential quality of existing studies, more high-quality studies are needed to validate this conclusion.

**Systematic Review Registration:**

https://www.crd.york.ac.uk/PROSPERO/view/, Identifier CRD420251251933.

## 1 Introduction

Diabetic nephropathy (DN) is one of the most common microvascular complications of diabetes mellitus (DM). It manifests clinically as progressive loss of renal function (estimated glomerular filtration rate <60 mL/min/1.73 m^2^ for more than 3 months) and/or persistent albuminuria (urinary albumin >300 mg/day or albumin-to-creatinine ratio >0.3) (Martinez Leon et al., 2026). The prevalence of DN in the DM population ranges from 20% to 40%, which makes it the primary cause of end-stage renal disease requiring dialysis or kidney transplantation (Watanabe-Shimoji et al., 2025). Furthermore, its 10-year cumulative mortality rate reaches as high as 31.1%, resulting in significant personal health damage and substantial public economic burden (Yao et al., 2022). Continuous hyperglycemia in DM triggers a series of maladaptive cellular events and a self-reinforcing cycle of injury in the kidney, leading to glomerular hyperfiltration, podocyte damage, tubulointerstitial inflammation and fibrosis, and oxidative stress (Martinez Leon et al., 2026). These represent key mechanisms driving the development of DN. Management of this disease is based on controlling blood glucose and blood pressure, with RAAS inhibitors, SGLT2 inhibitors, GLP-1 receptor agonists, and non-steroidal mineralocorticoid receptor antagonists being the mainstream treatments, but patients still frequently encounter residual renal risks (Agarwal, 2025). Given the complex pathogenesis and current treatment status, existing therapies remain insufficient. Consequently, it is urgent to find effective drugs to slow the progression of DN.

Natural products have attracted considerable attention due to their role in drug development and disease treatment (Chopra and Dhingra, 2021). *Tripterygium wilfordii Hook. f.* is a common traditional Chinese medicine (TCM). According to the *Supplement to the Compendium of Materia Medica*, it is effective in dispelling wind and dampness, promoting blood circulation and unblocking meridians, reducing swelling and relieving pain, as well as killing parasites and neutralizing toxins (Jin et al., 2025). Tripterygium glycosides (TGs) are active metabolites extracted and purified from the roots of this botanical drug. They predominantly consist of diterpenoids, triterpenoids, and alkaloids, possessing anti-inflammatory and immunomodulatory properties (Liu et al., 2021; Wu et al., 2017; Zhang et al., 2022) (Table 1). In China, TGs have long been applied in clinical treatment of DN, and have also demonstrated therapeutic value in autoimmune diseases such as rheumatoid arthritis, systemic lupus erythematosus, and psoriasis (Jin et al., 2026). A study evaluated the clinical efficacy and safety of TGs combined with ACEI/ARB in the treatment of DN, indicating that combined therapy was more effective than ACEI/ARB alone in decreasing 24-h urinary protein (24 h UP) and serum creatinine (Scr), and increasing serum albumin (ALB) and effective rate, but with a higher risk of adverse events (Yao et al., 2024). Existing studies suggest that TGs can achieve anti-DN effects through multiple interrelated pharmacological mechanisms, including anti-inflammatory, countering oxidative stress, inhibiting fibrosis, protecting podocytes, and regulating autophagy and apoptosis (Jin et al., 2025; Liu et al., 2021).

**TABLE 1:** The main metabolites of TGs.

Metabolite name	Chemical category	Molecular formula
Triptolide	Diterpenoid	C_20_H_24_O_6_
Triptonide	Diterpenoid	C_20_H_22_O_6_
Celastrol	Triterpenoid	C_29_H_38_O_4_
Wilforlide A	Triterpenoid	C_30_H_46_O_3_
Wilforgine	Alkaloid	C_41_H_47_NO_19_
Wilforine	Alkaloid	C_43_H_49_NO_18_
Wilfordine	Alkaloid	C_43_H_49_NO_19_
Wilfortrine	Alkaloid	C_41_H_47_NO_20_

However, there is currently a lack of relevant systematic summaries, and the methods for balancing the efficacy and safety of TGs in treating DN remain unclear, which limits their wide application to some extent. In clinical practice, TGs are used to treat DN, rather than the individual metabolites they contain (such as triptolide). Furthermore, TGs differ from their individual metabolites in terms of pharmacokinetics and toxicity profiles, and including both would introduce heterogeneity in the intervention. Therefore, this study focuses on TGs as the intervention. Preclinical systematic review and meta-analysis can provide evidence-based support for the assessment and transformation of TGs. Understanding the existing mechanisms of TGs is crucial for designing novel research protocols. Therefore, this study aims to analyze the animal experiments of TGs in treating DN, evaluate their renal protective effects, summarize potential mechanisms, and preliminarily explore strategies for enhancing efficacy while reducing toxicity. This will provide reliable evidence for subsequent animal studies and clinical applications.

## 2 Materials and methods

This study was conducted following the Preferred Reporting Items for Systematic Reviews and Meta-Analyses (PRISMA) and registered in PROSPERO under the number CRD420251251933 (Page et al., 2021).

### 2.1 Search strategies

In eight databases, including four Chinese databases (CNKI, Wanfang, VIP, SinoMed) and four English databases (PubMed, Embase, Web of Science, Cochrane Library), relevant literature on the treatment of DN animal models with TGs was retrieved. The search period spanned from the establishment of each database to May 2026. The core search formula was: (“tripterygium glycosides”) and (“diabetic nephropathies”) and (“models, animal” or “animal experimentation” or “animals” or “rats” or “mice”). Use a combination of medical subject headings and free terms to formulate comprehensive search strategies. Detailed search strategies are presented in Supplementary File S1.

### 2.2 Eligibility criteria

#### 2.2.1 Inclusion criteria

(1) Population: DM animal models with renal outcomes induced by different methods. Species, strain, and sex are not restricted. (2) Intervention: TGs without limiting the intervention route, dose, and duration. (3) Comparison: A solvent without pharmacological effects or no intervention. (4) Outcomes: a. Renal function: Scr, blood urea nitrogen (BUN), 24 h UP, 24-h urinary albumin (24h UA), 24-h urinary microalbumin (24h UMA). Scr, BUN, and 24h UP are the main outcomes, and the literature must report one of these three to be included; b. Basic indicators: kidney weight (KW), kidney index (KI); c. Glucose and lipid metabolism: blood glucose (BG), total cholesterol (TC), triglycerides (TG); d. Safety: alanine aminotransferase (ALT), aspartate aminotransferase (AST), albumin (ALB), white blood cells (WBC), red blood cells (RBC), platelets (PLT); e. Renal tissue pathological changes: mean glomerular area (MGA), mean glomerular volume (MGV); f. Protective mechanisms: markers related to oxidative stress, inflammation, renal fibrosis, podocyte injury, and autophagy. (5) Study design: *In vivo* animal experiments.

#### 2.2.2 Exclusion criteria

(1) Duplicate publications (merged or retained literature with more complete data). (2) Clinical and *in vitro* studies. (3) Reviews, scientific achievements, and conference abstracts. (4) Literature lacking a DM control group. (5) Studies in which the experimental group used a metabolite of TGs or TGs in combination with other drugs. (6) Literature for which the full text and data are unavailable.

### 2.3 Data extraction

Two researchers independently read the titles, abstracts and full texts of the literature to identify studies meeting the criteria. In the event of disagreement, a third researcher was consulted. Then, two researchers independently extracted the following data using Excel tables: first author and publication year; animal species, strains, sex, and weight; modeling methods and success indicators; types of DM; interventions and sample sizes of experimental and control groups; administration route, dose, duration, and manufacturer of TGs; and outcomes. They would also check the consistency of the data with each other. If a study reported outcomes for multiple TGs doses or measurement time points, outcomes for all doses and the final time point were included. Data presented as standard error (SE) would be converted to standard deviation (SD) to standardise the format to mean and SD. When data were reported only as figures, we attempted to contact the authors. If there was no response, data were extracted via WebPlotDigitizer.

### 2.4 Risk of bias

Two researchers used SYRCLE‘s risk of bias tool to assess the bias risk and methodological quality of each included study (Hooijmans et al., 2014), and quantified scores from the following 10 domains: (1) sequence generation; (2) baseline characteristics; (3) allocation concealment; (4) random housing; (5) blinding animal breeders and researchers; (6) random outcome assessment; (7) blinding outcome assessors; (8) incomplete outcome data; (9) selective outcome reporting; (10) other sources of bias. Each domain could be rated as low risk of bias (Yes), high risk of bias (No), or uncertain risk of bias (Uncertain). If a domain was rated as “Yes”, the study received 1 point, with a maximum score of 10 points. When encountering disagreements, we will discuss them with the third researcher.

### 2.5 Evidence certainty

The GRADE method was used to evaluate the evidence certainty of important outcomes. The initial evidence grade of randomized controlled trials was high. Based on the five downgrading factors of bias risk, inconsistency, indirectness, imprecision, and publication bias, the quality of evidence was graded into four levels: high, moderate, low, and very low.

### 2.6 Data merging and splitting

For multi-arm studies involving different doses of TGs, we followed the Cochrane Handbook for Systematic Reviews of Interventions (CHSRI) to combine all dose experimental groups into one intervention group. The following formulas can be used to calculate the combined sample size, combined mean, and combined SD (Cumpston et al., 2019).

Combined sample size:N=N1+N2

$$\text{Combined mean}:Mean=\frac{N1M1+N2M2}{N1+N2}$$

$$\text{Combined SD}:SD=\sqrt{\frac{\left(N1-1\right){SD1}^{2}+\left(N2-1\right){SD2}^{2}+\frac{N1N2}{N1+N2}{\left(M1-M2\right)}^{2}}{N1+N2-1}}$$

Combining different experimental groups from multi-arm studies will result in the loss of key information about the dose-response relationship. Especially for drugs like TGs, which have well-defined toxicity and a narrow therapeutic window, identifying the appropriate dose within the safe range is of significant clinical value. Therefore, Scr, BUN, and 24h UP were used as efficacy indicators, while ALT, AST, and WBC were used as toxicity indicators. After equally splitting the common control group of the multi-arm study (control group sample size divided by the number of experimental groups) (Cumpston et al., 2019), TGs doses were grouped as low dose (<6 mg/kg/day), medium dose (6–9 mg/kg/day), and high dose (>9 mg/kg/day). Subgroup analysis can reflect the impact of different doses on the effect size, so it can be used to preliminarily explore the dose-effect-toxicity relationship and investigate whether dose leads to heterogeneity.

### 2.7 Statistical analysis

This meta-analysis was conducted with RevMan 5.4 and Stata 17.0. All outcomes in this study were continuous variables. When measurement methods and units were consistent across studies, results were quantified by the weighted mean difference (WMD) and 95% confidence interval (CI); otherwise, they were expressed as the standardised mean difference (SMD) and 95% CI. A difference was considered statistically significant if *P* < 0.05. Heterogeneity among studies was assessed using *I* ^*2*^. Studies with *I* ^*2*^ ≤ 50% were pooled using the fixed-effect model, while studies with *I* ^*2*^ > 50% were analyzed using the random-effect model. The sources of heterogeneity were explored by sensitivity analysis, subgroup analysis, or meta-regression analysis.

Leave-one-out sensitivity analysis can assess the robustness of results and identify sources of heterogeneity. Subgroup analysis will be conducted based on animal species (rats or mice), sex (male or female), type of DM (T1DM or T2DM), number of study arms (double-arm or multi-arm studies), and intervention duration (≤8 weeks or >8 weeks). When at least 10 studies report the same outcome, meta-regression analysis will be conducted on the modeling method, strain, manufacturer of TGs, STZ dose, methodological quality score, sample size, reference type (journal article or thesis), and publication year to further explore the factors causing heterogeneity. *P* < 0.05 means that this factor may be a source of heterogeneity. Furthermore, the funnel plot and Egger’s test were employed to assess publication bias, with asymmetry in the funnel plot or *P* < 0.05 indicating the presence of publication bias. For the outcomes with publication bias, the trim-and-fill method was used to estimate the number of missing studies, adjust the pooled effect size, and evaluate the robustness of the results.

## 3 Results

### 3.1 Literature screening

**FIGURE 1:**
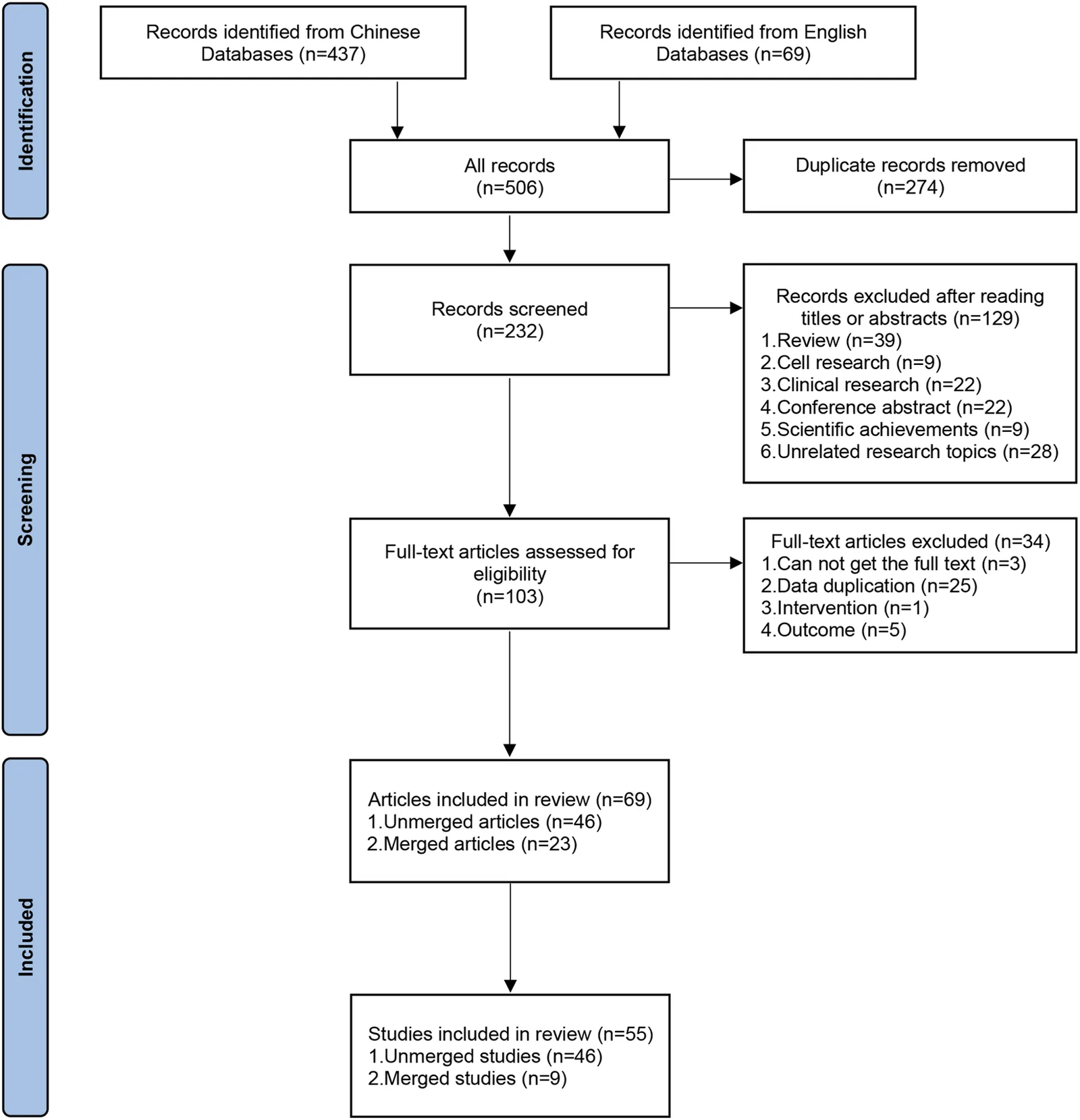
Literature screening flowchart.

A total of 506 articles were obtained by searching 8 databases, of which 437 were from Chinese databases (CNKI: 183; Wanfang: 115; VIP: 72; SinoMed: 67) and 69 were from English databases (PubMed: 11; EMBASE: 22; Web of Science: 36; Cochrane Library: 0). Firstly, 274 duplicate articles were excluded. Then, after title and abstract review, 129 articles were excluded, and a detailed full-text evaluation was conducted on the remaining 103 articles. Among them, the full text of 3 articles could not be obtained, 25 articles contained duplicate data, and 1 article’s intervention and 5 articles’ outcomes did not meet the inclusion criteria. Ultimately, 69 articles (representing 55 studies) were included in the analysis (Chang et al., 2014; Chang et al., 2012; Chang et al., 2021; Chen et al., 2023; Chen et al., 2015; Chi et al., 2009; Ding et al., 2017; Duan et al., 2023; Guo, 2013; Guo et al., 2025; He, 2020; He et al., 2026; Huang, 2015; Kong et al., 2021; Kong, 2013; Li, 2014; Li, 2016a; Li et al., 2016b; Li et al., 2019; Li and Yan, 2015; Li et al., 2012; Liang et al., 2026; Ling et al., 2016; Liu et al., 2014b; Liu et al., 2014a; Liu, 2013; Liu et al., 2017; Lu et al., 2015; Lv and Liu, 2009; Ma, 2015; Mao, 2016; Meng et al., 2020; Shen et al., 2014; Song et al., 2015; Song et al., 2023; Song et al., 2020; Song et al., 2026; Wang et al., 2016b; Wang et al., 2016a; Wang et al., 2008; Wang S. S., 2014; Wang X. Y., 2014; Wang et al., 2017; Wu et al., 2017; Xu et al., 2018; Yang, 2016; Yu et al., 2021; Zhang et al., 2025; Zhang et al., 2010; Zhang J. et al., 2013; Zhang et al., 2020; Zhang et al., 2022; Zhang J. et al., 2014; Zhang et al., 2016; Zhang et al., 2019; Zhang W. et al., 2014; Zhang et al., 2012; Zhang Y. et al., 2013; Zhang, 2014; Zhao et al., 2014; Zhao, 2013; Zhao et al., 2012; Zhao, 2014; Zhao, 2015; Zhao et al., 2017; Zhao, 2016; Zhao et al., 2015; Zhao et al., 2013; Zheng et al., 2011). The detailed literature screening process is shown in Figure 1.

### 3.2 Basic characteristics of included studies

In the 69 articles, 64 were published in Chinese and 5 in English. These articles corresponded to 55 experimental studies, of which 23 articles were merged into 9 experimental studies due to the same research characteristics and partially repeated outcomes, and 46 articles corresponded to the remaining 46 experimental studies. A total of 1,481 rodents (928 in the experimental group and 553 in the control group) were included, comprising 1,219 SD rats, 180 Wistar rats, and 82 C57BL/6 mice. In terms of sex, 49 studies used male animals, 3 used female animals, and 3 did not report sex. Regarding the type of DM, 33 studies focused on DN caused by T1DM and 22 focused on DN caused by T2DM. In the DN modeling methods, 25 studies used streptozotocin (STZ), 18 used high-fat and high-sugar diet (HFSD) combined with STZ, 8 used unilateral nephrectomy (UN) combined with STZ, 3 used high-fat diet (HFD) combined with STZ, and 1 used HFD combined with UN and STZ. Regarding the administration route, all studies used gavage (ig). The dose range of TGs was 0.1–1,800 mg/kg/day. Moreover, 34 studies used 1 dose, 9 used 2 doses, 11 used 3 doses, and 1 used 4 doses. Additionally, the intervention duration was 4–16 weeks. TGs originated from 11 manufacturers, with the top four in frequency of use being Jiangsu Meitong Pharmaceutical Co., Ltd. (12), Shanghai Fudan Fuhua Pharmaceutical Co., Ltd. (12), Zhejiang Deende Pharmaceutical Co., Ltd. (9), and Huangshi Feiyun Pharmaceutical Co., Ltd. (6). The basic characteristics of the included studies are shown in Supplementary Table S1.

### 3.3 Quality assessment

**FIGURE 2:**
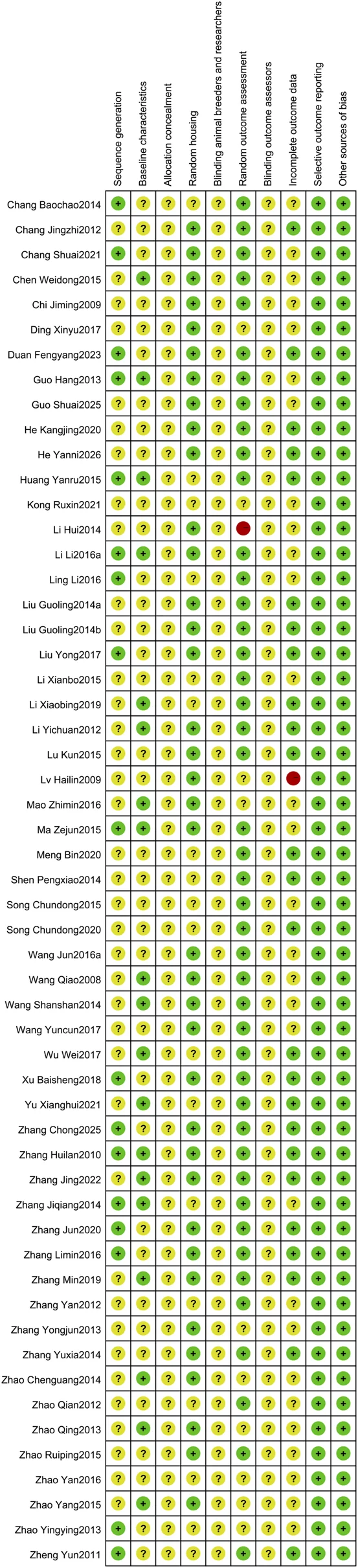
Risk of bias summary.

**FIGURE 3:**
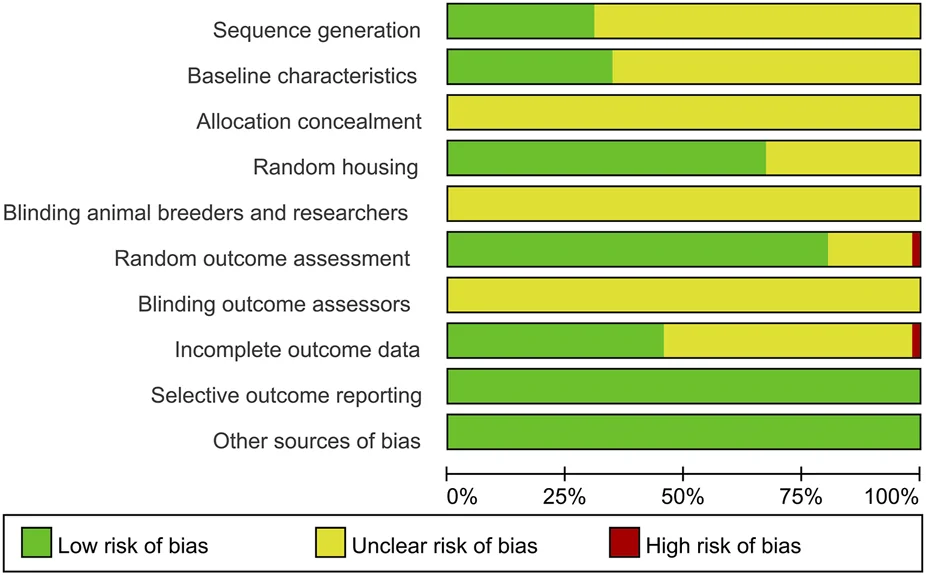
Risk of bias graph.

The methodological quality of the 55 included studies was assessed by the SYRCLE checklist. Their scores ranged from 2 to 7, with an average score of 4.58. Most of the studies were of moderate quality (80%), while a few were of low quality (20%). Seventeen studies described the specific methods of generating random sequences. Nineteen studies confirmed that the animal models were in similar baseline states. The above two aspects belong to the type of selective bias, and no study was rated as high risk of bias in this type. Thirty-seven studies indicated that animals were randomly placed in the same housing environment. In random outcome assessment, 44 studies randomly selected animals, 10 studies were at unclear risk of bias, and 1 study was at high risk of bias. Regarding incomplete outcome data, 25 studies reported complete data, 1 study had incomplete data reporting, and 29 studies were rated as having unclear risk of bias. No studies reported allocation concealment, blinding of animal breeders and researchers, or blinding of outcome assessors, resulting in unclear risks in these three domains. None of the studies found selective outcome reporting and other sources of bias, so they were rated as low bias risk (Figures 2, 3).

### 3.4 Effects of TGs on outcomes

The following six types of outcomes were analyzed, and the effects of TGs on outcomes in the DN animal model are summarized in Table 2.

**TABLE 2:** Effects of TGs on outcomes.

Outcomes	No. of studies	No. of animals	SMD [95% CI]	*P*-value for overall effect	Heterogeneity (*I* ^*2*^)
Renal function					
Scr	49	1299	−1.89 [-2.30, −1.49]	**<0.001**	87%
BUN	48	1335	−1.96 [-2.36, −1.56]	**<0.001**	86%
24h UP	29	812	−3.71 [-4.26, −3.16]	**<0.001**	80%
24h UA	6	220	−2.28 [-3.14, −1.41]	**<0.001**	77%
24h UMA	7	204	−4.30 [-6.32, −2.27]	**<0.001**	93%
Basic indicators					
KW	12	337	−1.19 [−1.58, −0.80]	**<0.001**	53%
KI	29	842	−1.97 [−2.46, −1.47]	**<0.001**	86%
Glucose and lipid metabolism					
BG	45	1197	−1.22 [−1.60, −0.84]	**<0.001**	86%
TC	12	349	−1.39 [−2.01, −0.77]	**<0.001**	80%
TG	12	349	−1.60 [−2.15, −1.05]	**<0.001**	74%
Safety					
ALT	17	451	−0.14 [−0.58, 0.30]	0.54	75%
AST	11	336	0.18 [−0.06, 0.42]	0.14	0%
ALB	10	181	1.29 [0.95, 1.62]	**<0.001**	3%
WBC	7	267	−0.10 [−0.37, 0.17]	0.46	49%
RBC	4	146	−0.20 [−0.57, 0.16]	0.27	0%
PLT	4	146	0.07 [−0.30, 0.43]	0.72	0%
Renal tissue pathological changes					
MGA	4	107	−2.19 [−2.70, −1.68]	**<0.001**	0%
MGV	7	167	−2.34 [−3.01, −1.68]	**<0.001**	59%
Protective mechanisms					
Oxidative stress					
SOD	3	67	4.67 [1.53, 7.82]	**0.004**	90%
Serum CAT	3	96	1.54 [1.03, 2.05]	**<0.001**	0%
GPx	2	72	1.23 [0.67, 1.79]	**<0.001**	29%
MDA	4	123	−2.37 [−3.64, −1.10]	**<0.001**	83%
Inflammation					
TNF-α	4	98	−2.33 [−3.76, −0.91]	**0.001**	81%
TNF-α mRNA	2	78	−1.95 [−2.54, −1.35]	**<0.001**	0%
IL-1β	3	65	−2.84 [−5.10, −0.59]	**0.01**	84%
IL-1β mRNA	1	45	−1.54 [−2.30, −0.79]	**<0.001**	-
MCP-1	3	83	−3.79 [−6.37, −1.22]	**0.004**	91%
MCP-1 mRNA	5	110	−2.71 [−3.27, −2.14]	**<0.001**	0%
VCAM-1	2	79	−2.45 [−3.08, −1.82]	**<0.001**	0%
VCAM-1 mRNA	2	79	−1.60 [−2.16, −1.05]	**<0.001**	0%
NF-κB	7	193	−3.04 [−4.01, −2.07]	**<0.001**	71%
NF-κB mRNA	5	124	−2.72 [−3.71, −1.73]	**<0.001**	59%
p-p38MAPK/p38MAPK	2	20	−3.41 [−8.04, 1.22]	0.15	82%
p-JAK2	2	74	−4.25 [−7.59, −0.91]	**0.01**	92%
JAK2 mRNA	1	34	0.61 [−0.20, 1.42]	0.14	-
p-STAT3	2	74	−4.34 [−6.46, −2.22]	**<0.001**	82%
STAT3 mRNA	1	34	0.00 [−0.79, 0.79]	1.00	-
Renal fibrosis					
TGF-β1	6	132	−3.46 [−4.91, −2.02]	**<0.001**	78%
TGF-β1 mRNA	6	167	−3.16 [−4.26, −2.07]	**<0.001**	77%
CTGF	2	53	−3.04 [−4.88, −1.20]	**0.001**	76%
CTGF mRNA	3	37	−3.23 [−4.37, −2.10]	**<0.001**	0%
α-SMA	2	55	−4.91 [−10.63, 0.81]	0.09	82%
α-SMA mRNA	3	80	−2.09 [−2.70, −1.49]	**<0.001**	0%
Col-IV	4	119	−2.95 [−4.20, −1.69]	**<0.001**	71%
Col-IV mRNA	7	167	−3.71 [−4.68, −2.73]	**<0.001**	61%
LN	2	68	−2.65 [−4.75, −0.55]	**0.01**	88%
LN mRNA	2	68	−4.11 [−5.02, −3.21]	**<0.001**	0%
Wnt1	2	29	−2.28 [−3.38, −1.18]	**<0.001**	32%
Wnt1 mRNA	2	22	−4.46 [−6.32, −2.61]	**<0.001**	0%
β-catenin	2	29	−2.22 [−3.31, −1.12]	**<0.001**	41%
β-catenin mRNA	3	42	−8.62 [−13.65, −3.58]	**<0.001**	75%
Podocyte injury					
Nephrin	5	113	3.54 [0.89, 6.18]	**0.009**	93%
Nephrin mRNA	5	128	2.85 [1.87, 3.83]	**<0.001**	68%
Podocin	3	63	6.67 [2.17, 11.17]	**0.004**	88%
Podocin mRNA	3	71	3.21 [2.44, 3.98]	**<0.001**	0%
VEGF	2	68	−2.91 [−4.93, −0.89]	**0.005**	86%
VEGF mRNA	3	86	−3.34 [−4.99, −1.68]	**<0.001**	81%
Autophagy					
p-PI3K/PI3K	2	40	−5.26 [−12.83, 2.30]	0.17	96%
p-PI3K	1	10	2.61 [0.69, 4.53]	**0.008**	-
PI3K	1	30	−0.51 [−1.41, 0.40]	0.27	-
p-Akt/Akt	3	50	−6.82 [−11.95, −1.68]	**0.009**	90%
p-Akt	1	52	−3.20 [−4.11, −2.29]	**<0.001**	-
p-Akt mRNA	1	52	−5.34 [−6.58, −4.09]	**<0.001**	-
Akt	2	82	−0.03 [−0.55, 0.49]	0.91	0%
Akt mRNA	1	52	0.00 [−0.65, 0.65]	1	-
p-mTOR/mTOR	2	20	−10.67 [−29.82, 8.48]	0.27	90%
p-mTOR	3	82	−3.11 [−5.18, −1.04]	**0.003**	77%
p-mTOR mRNA	1	52	−2.84 [−3.70, −1.98]	**<0.001**	-
mTOR	4	112	−0.21 [−0.99, 0.56]	0.59	53%
mTOR mRNA	2	58	−13.93 [−49.25, 21.39]	0.44	76%
LC3-II/LC3-I	2	40	6.37 [−2.84, 15.58]	0.18	93%
LC3 mRNA	2	40	9.05 [−5.23, 23.33]	0.21	94%
Beclin-1	2	40	3.93 [−0.89, 8.76]	0.11	90%
Beclin-1 mRNA	2	40	6.54 [−3.23, 16.31]	0.19	94%

Bold text indicates statistically significant differences between subgroups.

#### 3.4.1 Renal function

##### 3.4.1.1 Scr, BUN, 24h UP, 24h UA, and 24h UMA

**FIGURE 4:**
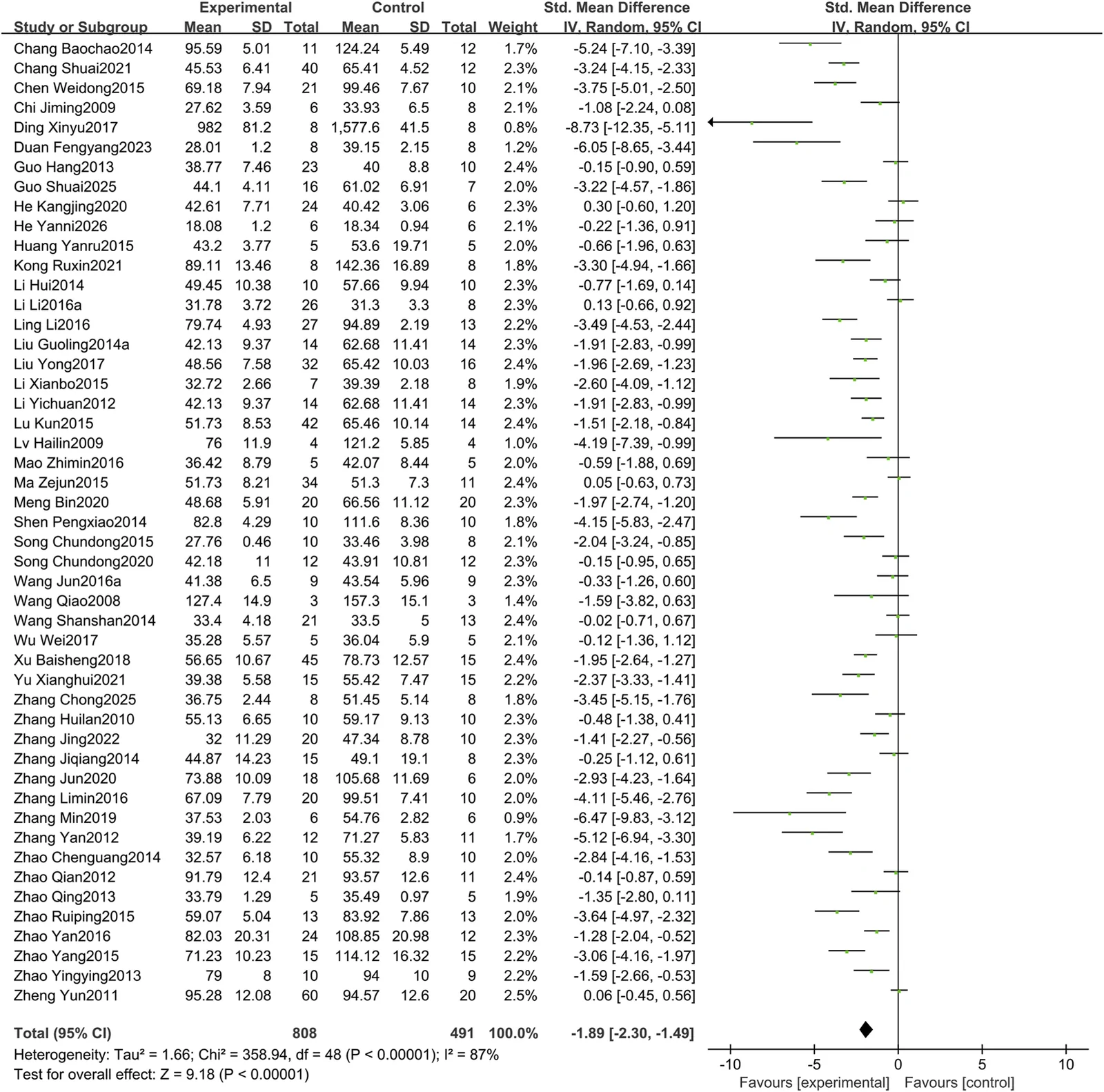
Forest plot of the effect of TGs on Scr.

**FIGURE 5:**
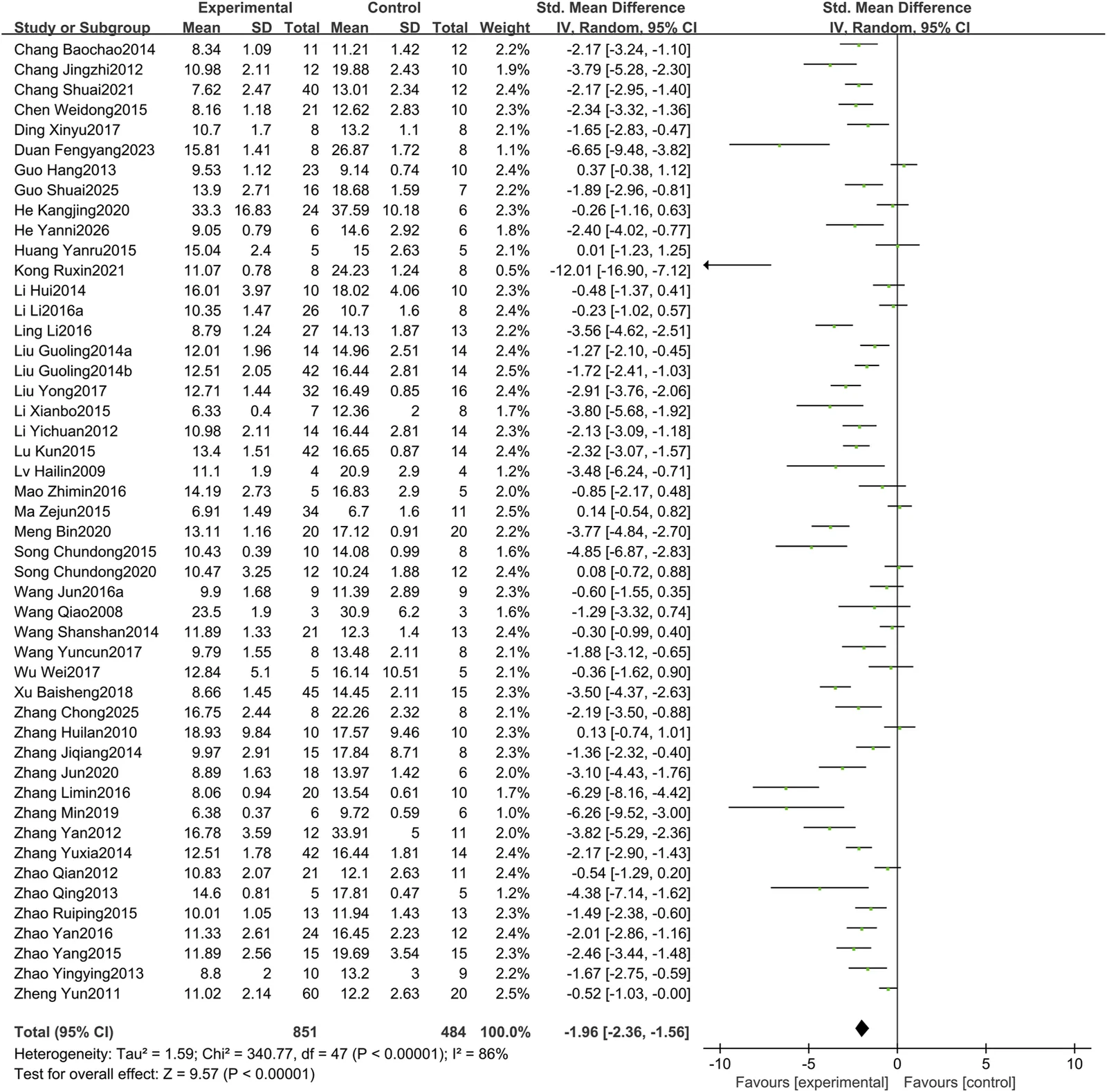
Forest plot of the effect of TGs on BUN.

**FIGURE 6:**
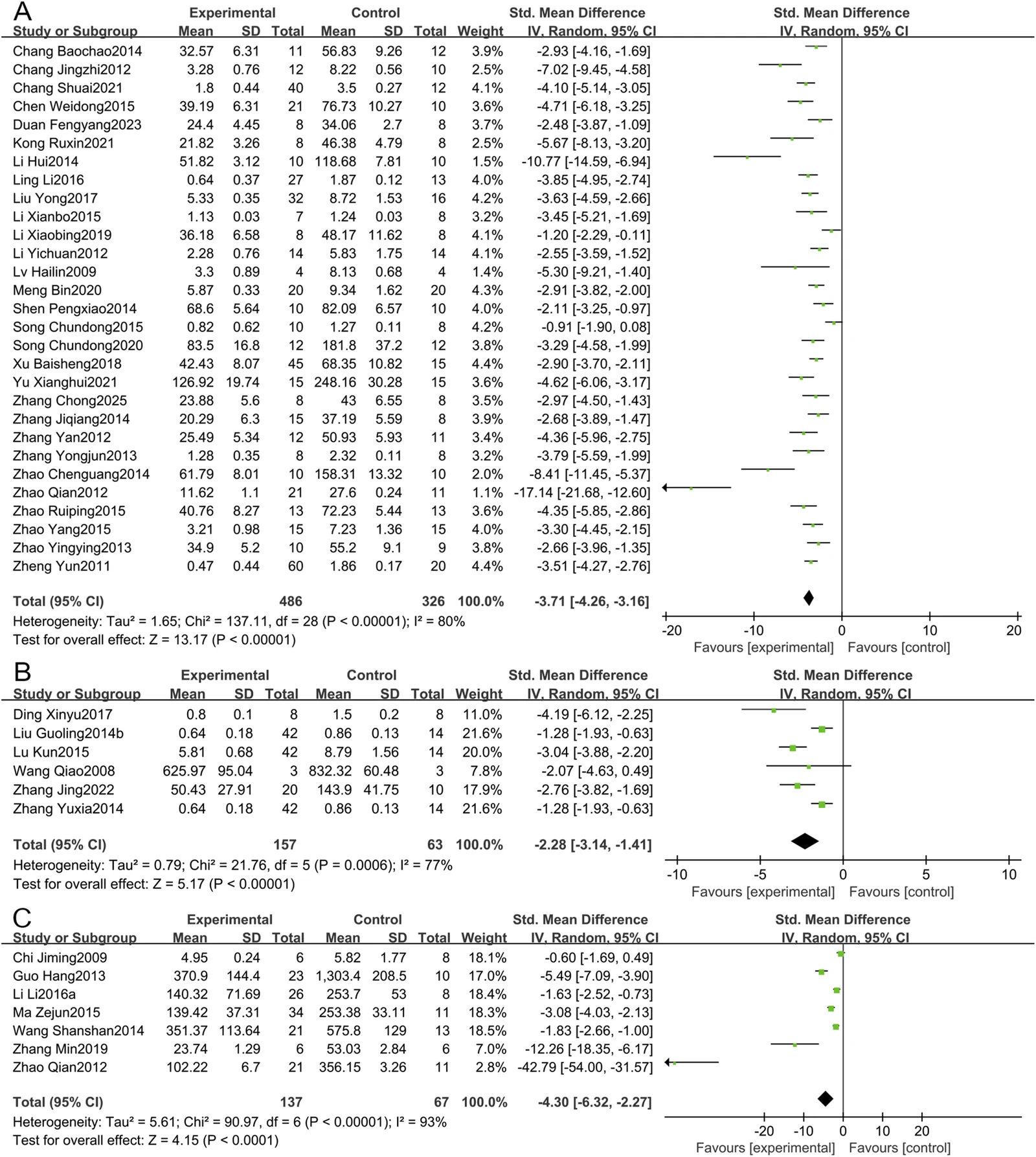
**(A)** Forest plot of the effect of TGs on 24h UP. **(B)** Forest plot of the effect of TGs on 24h UA. **(C)** Forest plot of the effect of TGs on 24h UMA.

Forty-nine studies (Chang et al., 2014; Chang et al., 2021; Chen et al., 2015; Chi et al., 2009; Ding et al., 2017; Duan et al., 2023; Guo, 2013; Guo et al., 2025; He, 2020; He et al., 2026; Huang, 2015; Kong et al., 2021; Li, 2014; Li, 2016a; Li and Yan, 2015; Li et al., 2012; Ling et al., 2016; Liu et al., 2014a; Liu et al., 2017; Lu et al., 2015; Lv and Liu, 2009; Ma, 2015; Mao, 2016; Meng et al., 2020; Shen et al., 2014; Song et al., 2015; Song et al., 2020; Wang et al., 2016a; Wang et al., 2008; Wang S. S., 2014; Wu et al., 2017; Xu et al., 2018; Yu et al., 2021; Zhang et al., 2025; Zhang et al., 2010; Zhang et al., 2020; Zhang et al., 2022; Zhang J. et al., 2014; Zhang et al., 2016; Zhang et al., 2019; Zhang et al., 2012; Zhao et al., 2014; Zhao, 2013; Zhao et al., 2012; Zhao, 2015; Zhao, 2016; Zhao et al., 2015; Zhao et al., 2013; Zheng et al., 2011) reported the effect of TGs on Scr in DM animals. Compared with the control group, TGs significantly reduced Scr levels (n = 1,299, SMD = −1.89, 95% CI [−2.30, −1.49], *P* < 0.001, *I* ^*2*^ = 87%) (Figure 4). Forty-eight studies (Chang et al., 2014; Chang et al., 2012; Chang et al., 2021; Chen et al., 2015; Ding et al., 2017; Duan et al., 2023; Guo, 2013; Guo et al., 2025; He, 2020; He et al., 2026; Huang, 2015; Kong et al., 2021; Li, 2014; Li, 2016a; Li and Yan, 2015; Li et al., 2012; Ling et al., 2016; Liu et al., 2014b; Liu et al., 2014a; Liu et al., 2017; Lu et al., 2015; Lv and Liu, 2009; Ma, 2015; Mao, 2016; Meng et al., 2020; Song et al., 2015; Song et al., 2020; Wang et al., 2016a; Wang et al., 2008; Wang S. S., 2014; Wang et al., 2017; Wu et al., 2017; Xu et al., 2018; Zhang et al., 2025; Zhang et al., 2010; Zhang et al., 2020; Zhang J. et al., 2014; Zhang et al., 2016; Zhang et al., 2019; Zhang et al., 2012; Zhang, 2014; Zhao, 2013; Zhao et al., 2012; Zhao, 2015; Zhao, 2016; Zhao et al., 2015; Zhao et al., 2013; Zheng et al., 2011) evaluated changes in BUN levels. TGs treatment significantly reduced BUN levels (n = 1,335, SMD = −1.96, 95% CI [−2.36, −1.56], *P* < 0.001, *I* ^*2*^ = 86%) (Figure 5). A random-effects model was used to pool 29 studies (Chang et al., 2014; Chang et al., 2012; Chang et al., 2021; Chen et al., 2015; Duan et al., 2023; Kong et al., 2021; Li, 2014; Li et al., 2019; Li and Yan, 2015; Li et al., 2012; Ling et al., 2016; Liu et al., 2017; Lv and Liu, 2009; Meng et al., 2020; Shen et al., 2014; Song et al., 2015; Song et al., 2020; Xu et al., 2018; Yu et al., 2021; Zhang et al., 2025; Zhang J. et al., 2014; Zhang et al., 2012; Zhang Y. et al., 2013; Zhao et al., 2014; Zhao et al., 2012; Zhao, 2015; Zhao et al., 2015; Zhao et al., 2013; Zheng et al., 2011) reporting 24 h UP, and the results showed that 24 h UP levels were significantly decreased after TGs intervention (n = 812, SMD = −3.71, 95% CI [−4.26, −3.16], *P* < 0.001, *I* ^*2*^ = 80%) (Figure 6A). Moreover, six studies (Ding et al., 2017; Liu et al., 2014b; Lu et al., 2015; Wang et al., 2008; Zhang et al., 2022; Zhang, 2014) analyzed 24h UA in DM animals, showing that TGs also decreased 24h UA levels (n = 220, SMD = −2.28, 95% CI [−3.14, −1.41], *P* < 0.001, *I* ^*2*^ = 77%) (Figure 6B). Seven studies (Chi et al., 2009; Guo, 2013; Li, 2016a; Ma, 2015; Wang S. S., 2014; Zhang et al., 2019; Zhao et al., 2012) evaluated the effect of TGs on 24h UMA. The pooled results showed that TGs could also reduce 24h UMA levels, but the heterogeneity between studies was high (n = 204, SMD = −4.30, 95% CI [−6.32, −2.27], *P* < 0.001, *I* ^*2*^ = 93%) (Figure 6C).

#### 3.4.2 Basic indicators

##### 3.4.2.1 KW and KI

**FIGURE 7:**
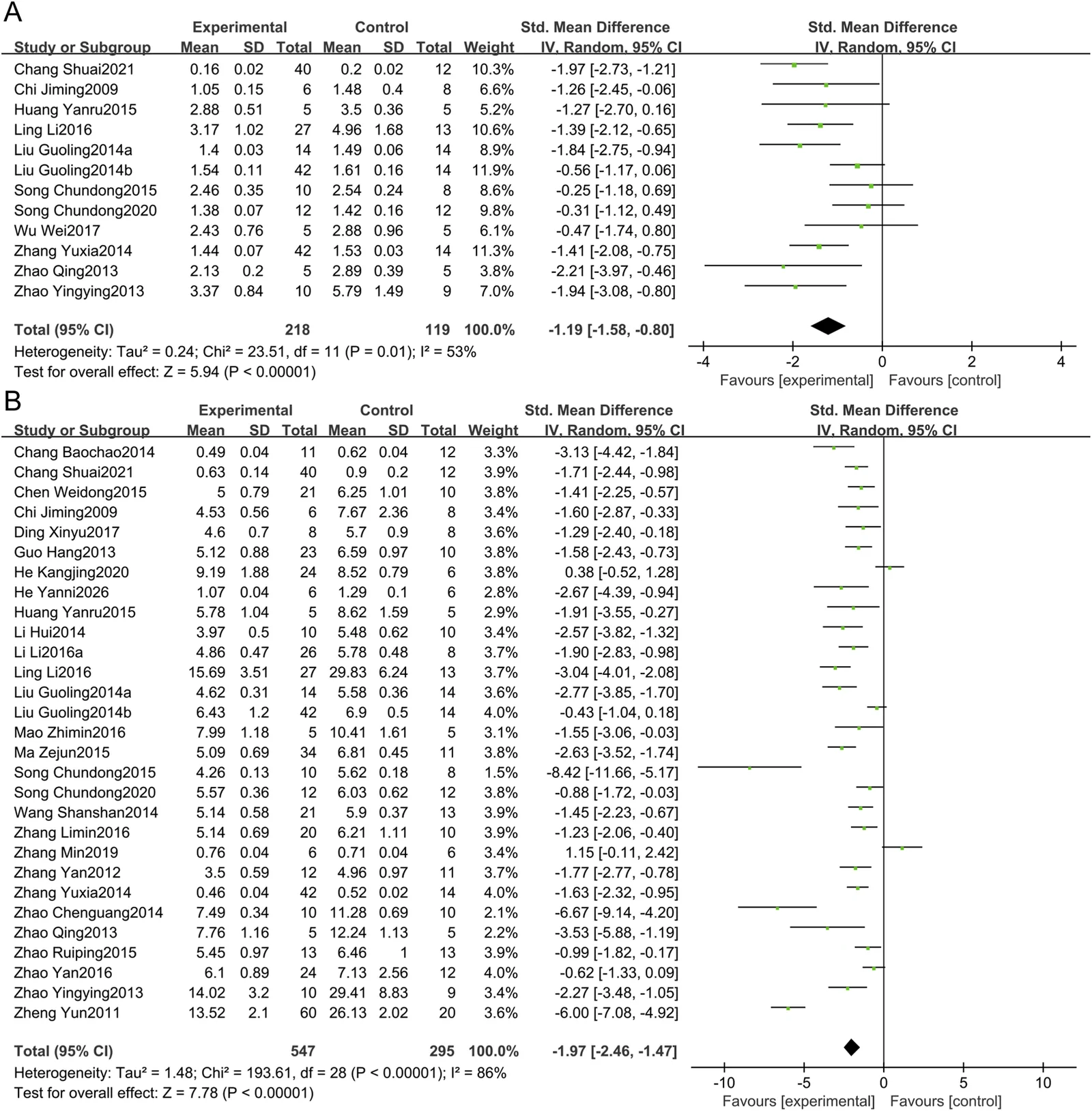
**(A)** Forest plot of the effect of TGs on KW. **(B)** Forest plot of the effect of TGs on KI.

An analysis of 12 studies (Chang et al., 2021; Chi et al., 2009; Huang, 2015; Ling et al., 2016; Liu et al., 2014b; Liu et al., 2014a; Song et al., 2015; Song et al., 2020; Wu et al., 2017; Zhang, 2014; Zhao, 2013; Zhao et al., 2013) reporting KW found that TGs treatment was beneficial to reduce KW of DM animals, with moderate heterogeneity (n = 337, SMD = −1.19, 95% CI [−1.58, −0.80], *P* < 0.001, *I* ^*2*^ = 53%) (Figure 7A). Meanwhile, 29 studies (Chang et al., 2014; Chang et al., 2021; Chen et al., 2015; Chi et al., 2009; Ding et al., 2017; Guo, 2013; He, 2020; He et al., 2026; Huang, 2015; Li, 2014; Li, 2016a; Ling et al., 2016; Liu et al., 2014b; Liu et al., 2014a; Ma, 2015; Mao, 2016; Song et al., 2015; Song et al., 2020; Wang S. S., 2014; Zhang et al., 2016; Zhang et al., 2019; Zhang et al., 2012; Zhang, 2014; Zhao et al., 2014; Zhao, 2013; Zhao, 2015; Zhao, 2016; Zhao et al., 2013; Zheng et al., 2011) assessed the effect of TGs on KI, indicating that it could significantly reduce KI (n = 842, SMD = −1.97, 95% CI [-2.46, −1.47], *P* < 0.001, *I* ^*2*^ = 86%) (Figure 7B).

#### 3.4.3 Glucose and lipid metabolism

##### 3.4.3.1 BG, TC, and TG

**FIGURE 8:**
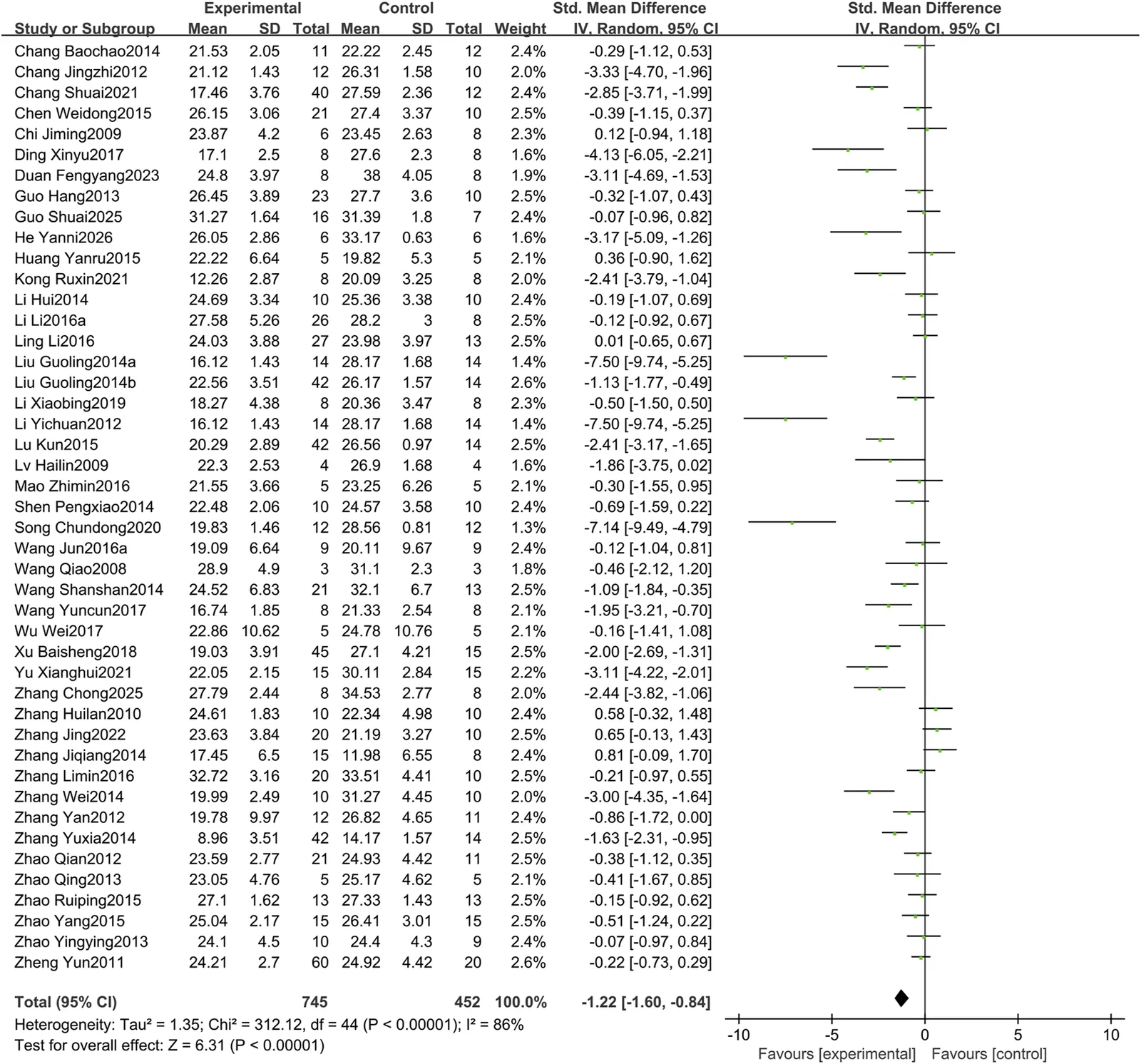
Forest plot of the effect of TGs on BG.

**FIGURE 9:**
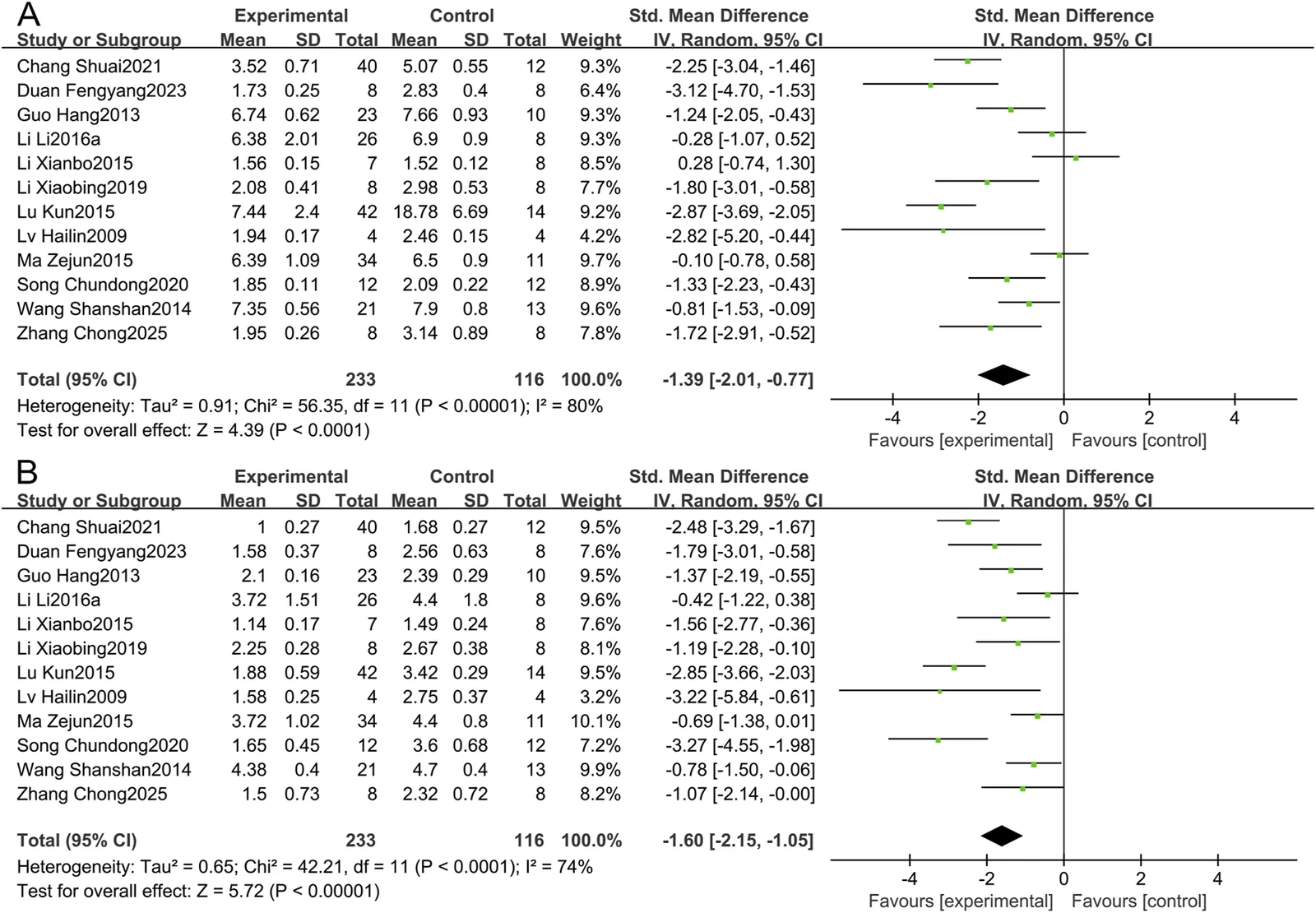
**(A)** Forest plot of the effect of TGs on TC. **(B)** Forest plot of the effect of TGs on TG.

Forty-five studies (Chang et al., 2014; Chang et al., 2012; Chang et al., 2021; Chen et al., 2015; Chi et al., 2009; Ding et al., 2017; Duan et al., 2023; Guo, 2013; Guo et al., 2025; He et al., 2026; Huang, 2015; Kong et al., 2021; Li, 2014; Li, 2016a; Li et al., 2019; Li et al., 2012; Ling et al., 2016; Liu et al., 2014b; Liu et al., 2014a; Lu et al., 2015; Lv and Liu, 2009; Mao, 2016; Shen et al., 2014; Song et al., 2020; Wang et al., 2016a; Wang et al., 2008; Wang S. S., 2014; Wang et al., 2017; Wu et al., 2017; Xu et al., 2018; Yu et al., 2021; Zhang et al., 2025; Zhang et al., 2010; Zhang et al., 2022; Zhang J. et al., 2014; Zhang et al., 2016; Zhang W. et al., 2014; Zhang et al., 2012; Zhang, 2014; Zhao, 2013; Zhao et al., 2012; Zhao, 2015; Zhao et al., 2015; Zhao et al., 2013; Zheng et al., 2011) involving 1,197 animals analyzed the BG levels. BG levels in the experimental group were markedly lower than those in the control group after TGs intervention (n = 1,197, SMD = −1.22, 95% CI [−1.60, −0.84], *P* < 0.001, *I* ^*2*^ = 86%) (Figure 8). Both TC and TG were reported in 12 of the same studies (Chang et al., 2021; Duan et al., 2023; Guo, 2013; Li, 2016a; Li et al., 2019; Li and Yan, 2015; Lu et al., 2015; Lv and Liu, 2009; Ma, 2015; Song et al., 2020; Wang S. S., 2014; Zhang et al., 2025). TGs treatment not only reduced serum TC levels (n = 349, SMD = −1.39, 95% CI [−2.01, −0.77], *P* < 0.001, *I* ^*2*^ = 80%) (Figure 9A), but also reduced serum TG levels (n = 349, SMD = −1.60, 95% CI [−2.15, −1.05], *P* < 0.001, *I* ^*2*^ = 74%) (Figure 9B).

#### 3.4.4 Safety

##### 3.4.4.1 ALT, AST, and ALB

**FIGURE 10:**
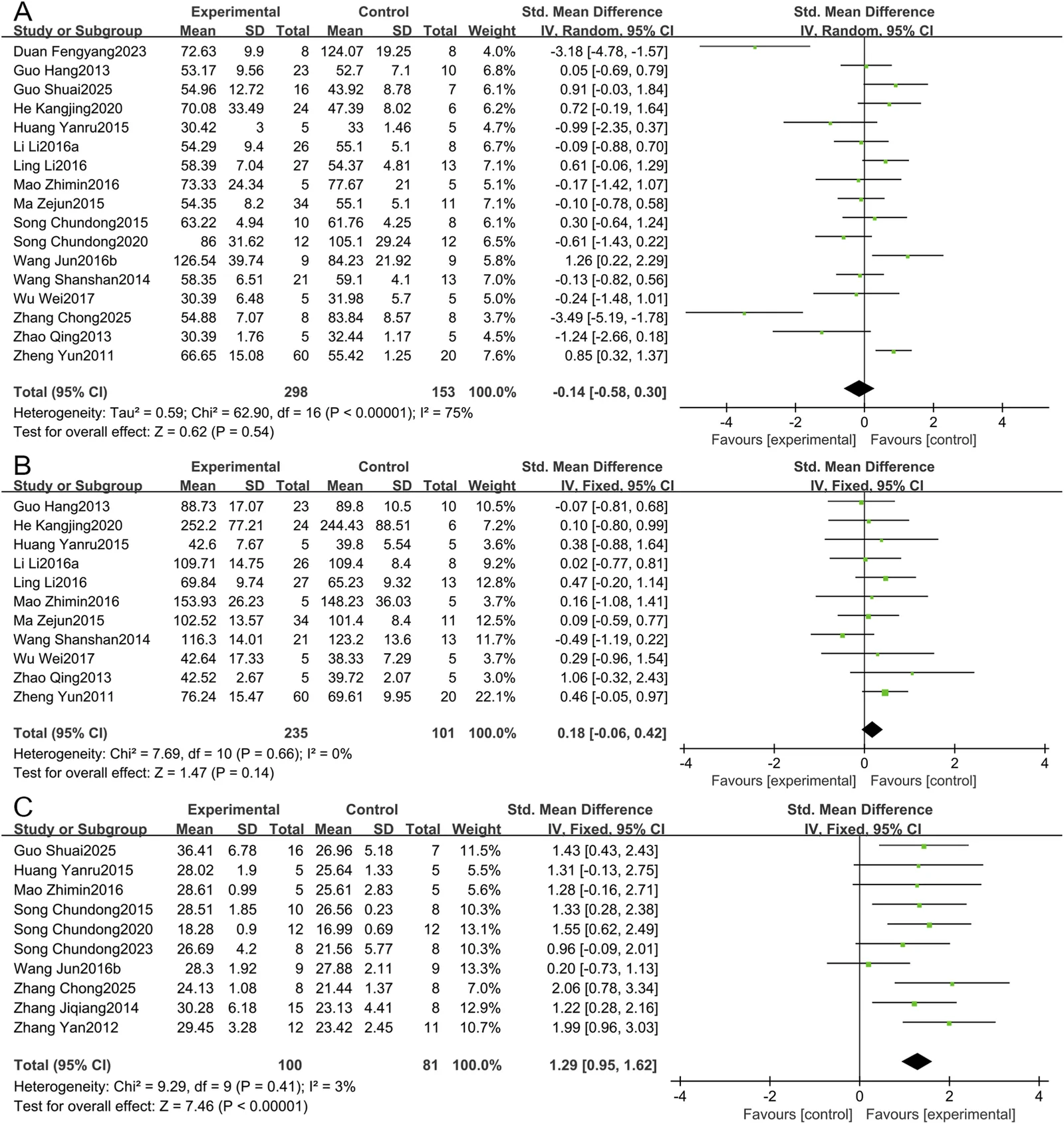
**(A)** Forest plot of the effect of TGs on ALT. **(B)** Forest plot of the effect of TGs on AST. **(C)** Forest plot of the effect of TGs on ALB.

After analyzing ALT in 17 studies (Duan et al., 2023; Guo, 2013; Guo et al., 2025; He, 2020; Huang, 2015; Li, 2016a; Ling et al., 2016; Ma, 2015; Mao, 2016; Song et al., 2015; Song et al., 2020; Wang et al., 2016b; Wang S. S., 2014; Wu et al., 2017; Zhang et al., 2025; Zhao, 2013; Zheng et al., 2011), it was found that TGs had no significant effect on ALT levels in DM animals (n = 451, SMD = −0.14, 95% CI [−0.58, 0.30], *P* = 0.54, *I* ^*2*^ = 75%) (Figure 10A). Furthermore, 11 studies (Guo, 2013; He, 2020; Huang, 2015; Li, 2016a; Ling et al., 2016; Ma, 2015; Mao, 2016; Wang S. S., 2014; Wu et al., 2017; Zhao, 2013; Zheng et al., 2011) also evaluated AST. The results did not observe that TGs markedly increased AST, and there was no heterogeneity between studies, so a fixed-effect model was used (n = 336, SMD = 0.18, 95% CI [−0.06, 0.42], *P* = 0.14, *I* ^*2*^ = 0%) (Figure 10B). Additionally, 10 studies (Guo et al., 2025; Huang, 2015; Mao, 2016; Song et al., 2015; Song et al., 2023; Song et al., 2020; Wang et al., 2016b; Zhang et al., 2025; Zhang J. et al., 2014; Zhang et al., 2012) evaluated the effect of TGs on serum ALB, and TGs intervention was found to help increase serum ALB levels (n = 181, SMD = 1.29, 95% CI [0.95, 1.62], *P* < 0.001, *I* ^*2*^ = 3%) (Figure 10C). It is worth noting that this is consistent with the reduction in 24h UA levels, suggesting that TGs treatment for DN is beneficial in reducing the loss of ALB in the body.

##### 3.4.4.2 WBC, RBC, and PLT

**FIGURE 11:**
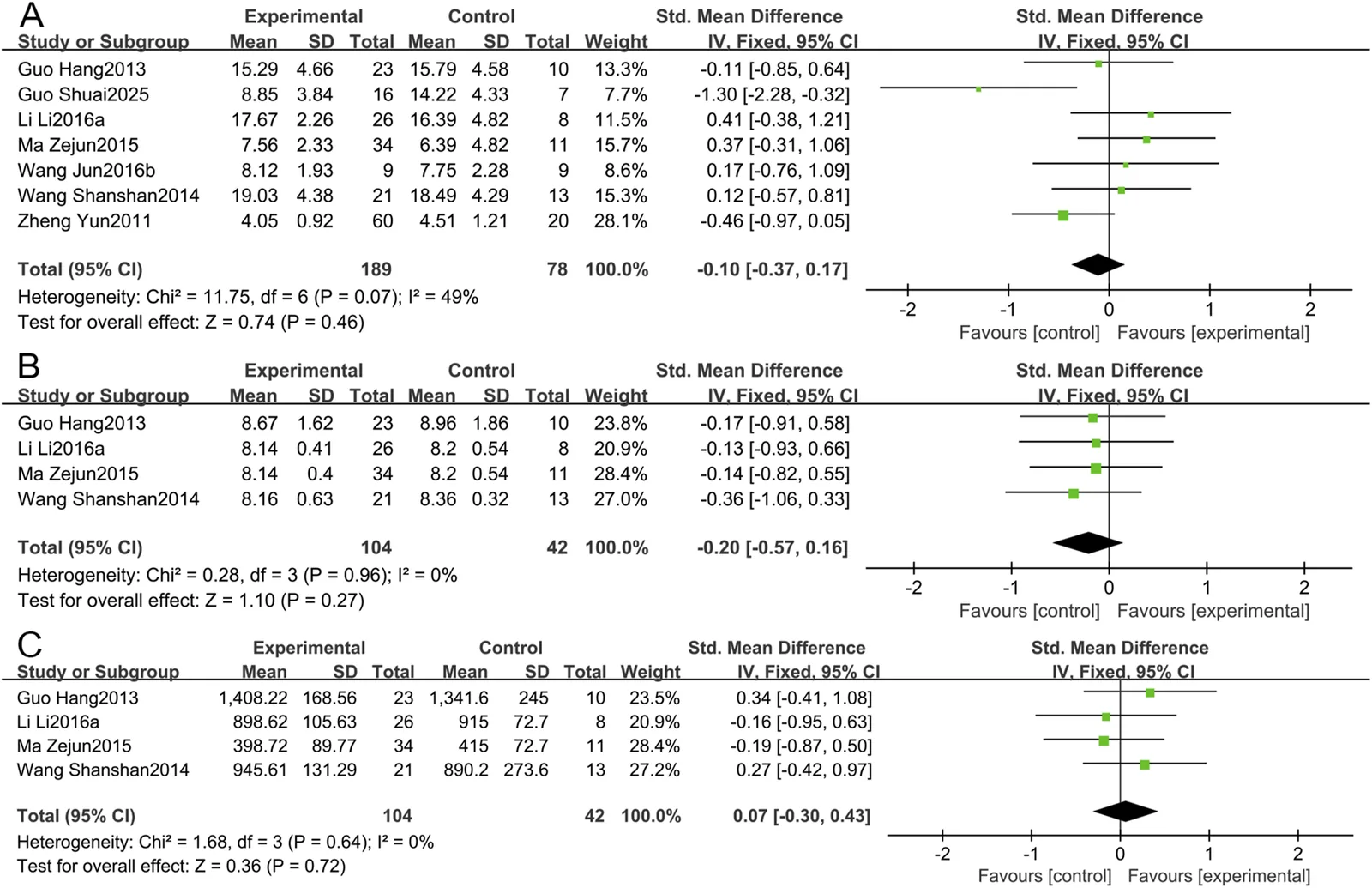
**(A)** Forest plot of the effect of TGs on WBC. **(B)** Forest plot of the effect of TGs on RBC. **(C)** Forest plot of the effect of TGs on PLT.

Seven studies (Guo, 2013; Guo et al., 2025; Li, 2016a; Ma, 2015; Wang et al., 2016b; Wang S. S., 2014; Zheng et al., 2011) involving 267 animals reported the effect of TGs on WBC. The analysis showed that there was no statistically significant difference in WBC levels between the TGs group and the control group (n = 267, SMD = −0.10, 95% CI [−0.37, 0.17], *P* = 0.46, *I* ^*2*^ = 49%) (Figure 11A). Four studies (Guo, 2013; Li, 2016a; Ma, 2015; Wang S. S., 2014) simultaneously assessed changes in RBC and PLT levels. Fixed-effect model analysis showed no significant effect of TGs on RBC levels (n = 146, SMD = −0.20, 95% CI [−0.57, 0.16], *P* = 0.27, *I* ^*2*^ = 0%) (Figure 11B) or PLT levels (n = 146, SMD = 0.07, 95% CI [−0.30, 0.43], *P* = 0.72, *I* ^*2*^ = 0%) (Figure 11C).

#### 3.4.5 Renal tissue pathological changes

##### 3.4.5.1 MGA and MGV

**FIGURE 12:**
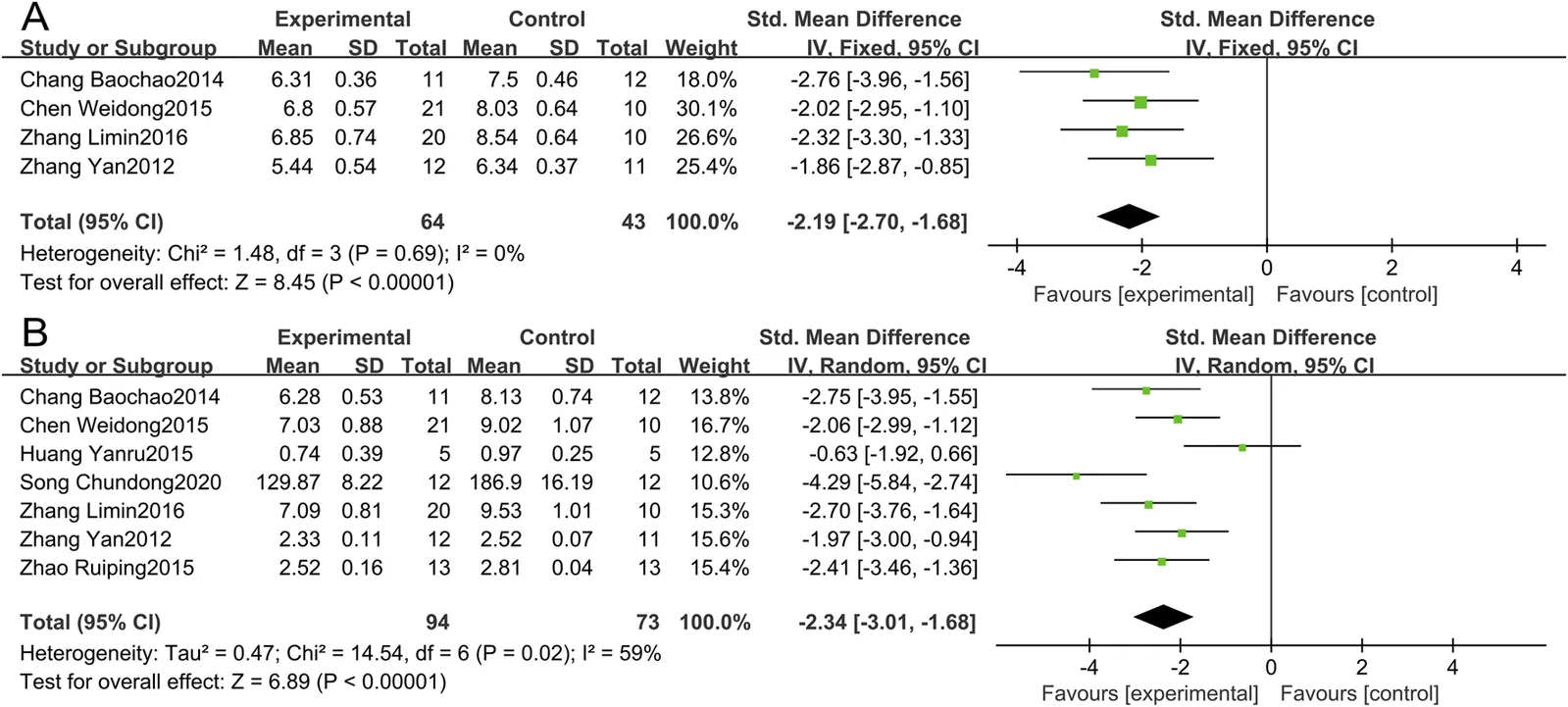
**(A)** Forest plot of the effect of TGs on MGA. **(B)** Forest plot of the effect of TGs on MGV.

Four comparisons (Chang et al., 2014; Chen et al., 2015; Zhang et al., 2016; Zhang et al., 2012) of renal MGA levels between the TGs group and the control group indicated that TGs treatment significantly lowered MGA levels (n = 107, SMD = −2.19, 95% CI [−2.70, −1.68], *P* < 0.001, *I* ^*2*^ = 0%) (Figure 12A). Seven studies (Chang et al., 2014; Chen et al., 2015; Huang, 2015; Song et al., 2020; Zhang et al., 2016; Zhang et al., 2012; Zhao, 2015) mentioned renal MGV. The combined results indicated that TGs led to a decrease in MGV levels (n = 167, SMD = −2.34, 95% CI [−3.01, −1.68], *P* < 0.001, *I* ^*2*^ = 59%) (Figure 12B).

#### 3.4.6 Protective mechanisms

##### 3.4.6.1 Markers related to oxidative stress

###### 3.4.6.1.1 SOD, serum CAT, and GPx

Three studies (Ding et al., 2017; Li et al., 2012; Zhang J. et al., 2013) analyzed renal SOD levels, while another three studies (Li et al., 2019; Zhang et al., 2020; Zhang, 2014) analyzed serum CAT levels. The results indicated that TGs significantly increased renal SOD levels (n = 67, SMD = 4.67, 95% CI [1.53, 7.82], *P* = 0.004, *I* ^*2*^ = 90%) (Supplementary Material S2.1A) and serum CAT levels (n = 96, SMD = 1.54, 95% CI [1.03, 2.05], *P* < 0.001, *I* ^*2*^ = 0%) (Supplementary Material S2.1B). Renal GPx was reported in 2 studies (Li et al., 2019; Zhang, 2014), and the combined results indicated that TGs helped to increase GPx expression (n = 72, SMD = 1.23, 95% CI [0.67, 1.79], *P* < 0.001, *I* ^*2*^ = 29%) (Supplementary Material S2.1C).

###### 3.4.6.1.2 MDA

Four studies (Li et al., 2019; Li et al., 2012; Zhang J. et al., 2013; Zhang, 2014) analyzed renal MDA levels, and the results showed that TGs reduced MDA levels, with a statistically significant difference (n = 123, SMD = −2.37, 95% CI [-3.64, −1.10], *P* < 0.001, *I* ^*2*^ = 83%) (Supplementary Material S2.1D).

##### 3.4.6.2 Markers related to inflammation

###### 3.4.6.2.1 TNF-α and TNF-α mRNA, IL-1β and IL-1β mRNA

A random-effect model was used to analyze TNF-α in four studies (Kong, 2013; Ma, 2015; Wu et al., 2017; Zhao, 2013), and a fixed-effect model was used to analyze TNF-α mRNA in two studies (Kong, 2013; Ma, 2015). The results indicated that TGs lowered renal TNF-α levels (n = 98, SMD = −2.33, 95% CI [-3.76, −0.91], *P* = 0.001, *I* ^*2*^ = 81%) and TNF-α mRNA expression (n = 78, SMD = −1.95, 95% CI [−2.54, −1.35], *P* < 0.001, *I* ^*2*^ = 0%) (Supplementary Material S2.2). Additionally, three studies (Ma, 2015; Wu et al., 2017; Zhao, 2013) assessed IL-1β, and one study (Mao, 2016) assessed IL-1β mRNA. Analysis found that TGs could lower IL-1β levels (n = 65, SMD = −2.84, 95% CI [−5.10, −0.59], *P* = 0.01, *I* ^*2*^ = 84%) and IL-1β mRNA expression in the kidneys of DM animals (Supplementary Material S2.3).

###### 3.4.6.2.2 MCP-1 and MCP-1 mRNA, VCAM-1 and VCAM-1 mRNA

MCP-1 (Wang X. Y., 2014; Zhang J. et al., 2014; Zhao et al., 2017) and MCP-1 mRNA (Chi et al., 2009; Guo, 2013; Wang X. Y., 2014; Zhang J. et al., 2014; Zhao et al., 2017) were reported by three and five studies, respectively. Compared with the control group, TGs decreased renal MCP-1 levels (n = 83, SMD = −3.79, 95% CI [−6.37, −1.22], *P* = 0.004, *I* ^*2*^ = 91%) and MCP-1 mRNA expression (n = 110, SMD = −2.71, 95% CI [−3.27, −2.14], *P* < 0.001, *I* ^*2*^ = 0%) (Supplementary Material S2.4). Two studies (Ma, 2015; Wang X. Y., 2014) simultaneously reported the changes in VCAM-1 and VCAM-1 mRNA levels. The results showed that there was no heterogeneity for both, and TGs helped to decrease renal VCAM-1 levels (n = 79, SMD = −2.45, 95% CI [−3.08, −1.82], *P* < 0.001, *I* ^*2*^ = 0%) and VCAM-1 mRNA expression (n = 79, SMD = −1.60, 95% CI [−2.16, −1.05], P < 0.001, *I* ^*2*^ = 0%) (Supplementary Material S2.5).

###### 3.4.6.2.3 NF-κB and NF-κB mRNA

Seven studies (Chen et al., 2023; Ma, 2015; Wu et al., 2017; Xu et al., 2018; Yang, 2016; Zhang et al., 2019; Zhao et al., 2017) analyzed NF-κB, and five studies (Chen et al., 2023; Guo, 2013; Ma, 2015; Yang, 2016; Zhao et al., 2017) analyzed NF-κB mRNA. Renal NF-κB levels (n = 193, SMD = −3.04, 95% CI [−4.01, −2.07], *P* < 0.001, *I* ^*2*^ = 71%) and NF-κB mRNA expression (n = 124, SMD = −2.72, 95% CI [−3.71, −1.73], *P* < 0.001, *I* ^*2*^ = 59%) in DM animals treated with TGs were both decreased (Supplementary Material S2.6).

###### 3.4.6.2.4 p-p38MAPK/p38MAPK, p-JAK2 and JAK2 mRNA, p-STAT3 and STAT3 mRNA

After pooling the two studies (Wu et al., 2017; Zhao, 2013), no significant effect of TGs on the p-p38MAPK/p38MAPK ratio was observed (n = 20, SMD = −3.41, 95% CI [−8.04, 1.22], *P* = 0.15, *I* ^*2*^ = 82%) (Supplementary Material S2.7). Two studies (Li, 2016a; Meng et al., 2020) evaluated the effects of TGs on p-JAK2 and p-STAT3 levels. The analysis suggests that TGs could diminish renal p-JAK2 levels (n = 74, SMD = −4.25, 95% CI [−7.59, −0.91], *P* = 0.01, *I* ^*2*^ = 92%) and p-STAT3 levels (n = 74, SMD = −4.34, 95% CI [−6.46, −2.22], *P* < 0.001, *I* ^*2*^ = 82%) (Supplementary Materials S2.8A, S2.9A). However, one study (Li, 2016a) indicated that TGs did not exert regulatory effects on the expression of JAK2 mRNA and STAT3 mRNA at the gene level (Supplementary Materials S2.8B, S2.9B).

##### 3.4.6.3 Markers related to renal fibrosis

###### 3.4.6.3.1 TGF-β1 and TGF-β1 mRNA, CTGF and CTGF mRNA

TGF-β1 (Huang, 2015; Ma, 2015; Wu et al., 2017; Yang, 2016; Zhang et al., 2012; Zhao, 2013) and TGF-β1 mRNA (Ding et al., 2017; Liu, 2013; Ma, 2015; Yang, 2016; Zhang et al., 2012; Zhang Y. et al., 2013) were each mentioned in 6 studies. Data were pooled using a random-effect model, and the results indicated that TGF-β1 levels (n = 132, SMD = −3.46, 95% CI [-4.91, −2.02], *P* < 0.001, *I* ^*2*^ = 78%) and TGF-β1 mRNA expression (n = 167, SMD = −3.16, 95% CI [−4.26, −2.07], *P* < 0.001, *I* ^*2*^ = 77%) in the kidneys of DM animals were significantly decreased (Supplementary Material S2.10). Moreover, 2 studies (Zhang J. et al., 2014; Zhao et al., 2015) mentioned CTGF, and 3 studies (Lv and Liu, 2009; Wang et al., 2008; Zhang J. et al., 2014) mentioned CTGF mRNA, indicating that TGs decreased renal CTGF levels (n = 53, SMD = −3.04, 95% CI [−4.88, −1.20], *P* = 0.001, *I* ^*2*^ = 76%) and TGF-β1 mRNA expression (n = 37, SMD = −3.23, 95% CI [−4.37, −2.10], *P* < 0.001, *I* ^*2*^ = 0%) (Supplementary Material S2.11).

###### 3.4.6.3.2 α-SMA and α-SMA mRNA, Col-IV and Col-IV mRNA, LN and LN mRNA

Two studies (Ma, 2015; Zhao, 2013) reported α-SMA, and three studies (Guo et al., 2025; Ma, 2015; Zhao et al., 2014) reported α-SMA mRNA. Although TGs had no significant effect on renal α-SMA levels (n = 55, SMD = −4.91, 95% CI [−10.63, 0.81], *P* = 0.09, *I* ^*2*^ = 82%), they did reduce α-SMA mRNA expression (n = 80, SMD = −2.09, 95% CI [−2.70, −1.49], *P* < 0.001, *I* ^*2*^ = 0%) (Supplementary Material S2.12). The analysis of four studies (Ma, 2015; Song et al., 2026; Wang S. S., 2014; Yang, 2016) reporting Col-IV and seven studies (Li, 2014; Ma, 2015; Song et al., 2026; Wang S. S., 2014; Yang, 2016; Zhang Y. et al., 2013; Zhao et al., 2014) reporting Col-IV mRNA indicated that TGs reduced Col-IV mRNA expression at the genetic level (n = 167, SMD = −3.71, 95% CI [−4.68, −2.73], *P* < 0.001, *I* ^*2*^ = 61%), thereby reducing renal Col-IV protein levels (n = 119, SMD = −2.95, 95% CI [−4.20, −1.69], *P* < 0.001, *I* ^*2*^ = 71%) (Supplementary Material S2.13). Two studies (Wang S. S., 2014; Yang, 2016) simultaneously reported LN and LN mRNA. Renal LN levels (n = 68, SMD = −2.65, 95% CI [−4.75, −0.55], *P* = 0.01, *I* ^*2*^ = 88%) and LN mRNA expression (n = 68, SMD = −4.11, 95% CI [−5.02, −3.21], *P* < 0.001, *I* ^*2*^ = 0%) in the TGs group were lower than those in the control group (Supplementary Material S2.14).

###### 3.4.6.3.3 Wnt1 and Wnt1 mRNA, β-catenin and β-catenin mRNA

Two studies each analyzed changes in Wnt1 (Chang et al., 2014; Guo et al., 2025) and Wnt1 mRNA (Chang et al., 2014; Kong et al., 2021) levels, showing that TGs resulted in a reduction in renal Wnt1 levels (n = 29, SMD = −2.28, 95% CI [−3.38, −1.18], *P* < 0.001, *I* ^*2*^ = 32%) and Wnt1 mRNA expression (n = 22, SMD = −4.46, 95% CI [−6.32, −2.61], *P* < 0.001, *I* ^*2*^ = 0%), with low heterogeneity. Therefore, the fixed-effect model was adopted (Supplementary Material S2.15). Two studies (Chang et al., 2014; Guo et al., 2025) analyzed β-catenin, and three studies (Chang et al., 2014; Kong et al., 2021; Li, 2014) analyzed β-catenin mRNA. The results found that renal β-catenin levels (n = 29, SMD = −2.22, 95% CI [-3.31, −1.12], *P* < 0.001, *I* ^*2*^ = 41%) and β-catenin mRNA expression (n = 42, SMD = −8.62, 95% CI [−13.65, −3.58], *P* < 0.001, *I* ^*2*^ = 75%) were diminished (Supplementary Material S2.16).

##### 3.4.6.4 Markers related to podocyte injury

###### 3.4.6.4.1 Nephrin and Nephrin mRNA, podocin and podocin mRNA

Five studies (Huang, 2015; Li et al., 2016b; Wang et al., 2017; Zhao, 2014; Zhao et al., 2013) involving 113 animals evaluated Nephrin, and another five studies (Guo et al., 2025; Li et al., 2016b; Wang et al., 2016b; Zhao, 2014; Zhao et al., 2013) involving 128 animals evaluated Nephrin mRNA. The pooled analysis indicated that renal Nephrin levels (n = 113, SMD = 3.54, 95% CI [0.89, 6.18], *P* = 0.009, *I* ^*2*^ = 93%) and Nephrin mRNA expression (n = 128, SMD = 2.85, 95% CI [1.87, 3.83], *P* < 0.001, *I* ^*2*^ = 68%) in the experimental group were significantly higher than those in the control group (Supplementary Material S2.17). Three studies each evaluated Podocin (Li et al., 2016b; Mao, 2016; Zhao et al., 2013) and Podocin mRNA (Li et al., 2016b; Wang et al., 2016b; Zhao et al., 2013). The results showed that TGs increased renal Podocin levels (n = 63, SMD = 6.67, 95% CI [2.17, 11.17], *P* = 0.004, *I* ^*2*^ = 88%) and Podocin mRNA expression (n = 71, SMD = 3.21, 95% CI [2.44, 3.98], *P* < 0.001, *I* ^*2*^ = 0%) (Supplementary Material S2.18).

###### 3.4.6.4.2 VEGF and VEGF mRNA

The analysis of two studies (Li et al., 2016b; Zhao, 2014) reporting VEGF and three studies (Li et al., 2016b; Wang et al., 2016a; Zhao, 2014) reporting VEGF mRNA revealed that TGs contributed to lowering VEGF levels (n = 68, SMD = −2.91, 95% CI [−4.93, −0.89], *P* = 0.005, *I* ^*2*^ = 86%) and VEGF mRNA expression (n = 86, SMD = −3.34, 95% CI [−4.99, −1.68], *P* < 0.001, *I* ^*2*^ = 81%) in the kidneys of DM animals (Supplementary Material S2.19).

##### 3.4.6.5 Markers related to autophagy

###### 3.4.6.5.1 p-PI3K/PI3K, p-PI3K, and PI3K

Two studies (Mao, 2016; Yu et al., 2021) mentioned p-PI3K/PI3K, and one study each mentioned p-PI3K(Huang, 2015) and PI3K(He, 2020). No significant effect of TGs on the renal p-PI3K/PI3K ratio (n = 40, SMD = −5.26, 95% CI [−12.83, 2.30], *P* = 0.17, *I* ^*2*^ = 96%) or PI3K levels was found, but there was a decreasing trend. Additionally, renal p-PI3K levels were significantly elevated (Supplementary Material S2.20).

###### 3.4.6.5.2 p-Akt/Akt, p-Akt and p-Akt mRNA, Akt and Akt mRNA

Three comparisons (Huang, 2015; Mao, 2016; Yu et al., 2021) of p-Akt/Akt were conducted between the TGs group and the control group, revealing that TGs significantly reduced the renal p-Akt/Akt ratio (n = 50, SMD = −6.82, 95% CI [−11.95, −1.68], *P* = 0.009, *I* ^*2*^ = 90%). One study (Chang et al., 2021) found that renal p-Akt levels and p-Akt mRNA expression decreased significantly after TGs intervention. Two and one studies respectively found that TGs did not change renal Akt levels (Chang et al., 2021; He, 2020) (n = 82, SMD = −0.03, 95% CI [−0.55, 0.49], *P* = 0.91, *I* ^*2*^ = 0%) and Akt mRNA expression (Chang et al., 2021) (Supplementary Material S2.21).

###### 3.4.6.5.3 p-mTOR/mTOR, p-mTOR and p-mTOR mRNA, mTOR and mTOR mRNA

After combining the two studies (Huang, 2015; Mao, 2016), no statistically significant difference was observed in the renal p-mTOR/mTOR ratio between the TGs group and the control group (n = 20, SMD = −10.67, 95% CI [−29.82, 8.48], *P* = 0.27, *I* ^*2*^ = 90%). However, three studies (Chang et al., 2021; Liang et al., 2026; Zhang et al., 2020) reported that TGs reduced renal p-mTOR levels (n = 82, SMD = −3.11, 95% CI [−5.18, −1.04], *P* = 0.003, *I* ^*2*^ = 77%), and one study (Chang et al., 2021) reported that TGs reduced p-mTOR mRNA expression. Four studies (Chang et al., 2021; He, 2020; Liang et al., 2026; Zhang et al., 2020) reported mTOR, and two studies (Chang et al., 2021; Liang et al., 2026) reported mTOR mRNA. The pooled analysis suggested that TGs had no regulatory effect on renal mTOR levels (n = 112, SMD = −0.21, 95% CI [−0.99, 0.56], *P* = 0.59, *I* ^*2*^ = 53%) and mTOR mRNA expression (n = 58, SMD = −13.93, 95% CI [−49.25, 21.39], *P* = 0.44, *I* ^*2*^ = 76%) (Supplementary Material S2.22).

###### 3.4.6.5.4 LC3-II/LC3-I and LC3 mRNA, Beclin-1 and Beclin-1 mRNA

Two studies (Zhang et al., 2025; Zhang et al., 2020) simultaneously assessed LC3-II/LC3-I, LC3 mRNA, Beclin-1, and Beclin-1 mRNA in the kidneys. No significant effect of TGs on these outcomes was observed, but there was an upward trend (Supplementary Materials S2.23, S2.24). Notably, the individual effect sizes of both studies were positive, whereas the pooled results were negative. This might be attributed to the fact that the TGs dose of Zhang Chong 2025 (Zhang et al., 2025) (6.25 mg/kg/d) was much higher than that of Zhang Jun 2020 (Zhang et al., 2020) (0.1/0.5/1 mg/kg/d), resulting in a large heterogeneity. Using a random-effect model would widen the CI. Therefore, the results of the two studies were reported separately to avoid misleading conclusions. Both studies indicated that TGs significantly increased these autophagy markers.

### 3.5 Sensitivity analysis

Given the limited number of studies on mechanism-related biomarkers, no sensitivity analysis was conducted for these outcomes. When heterogeneity among studies exceeds 50%, a sensitivity analysis is performed for that outcome. After excluding each study one by one, the upper limits of the 95% CIs for Scr, BUN, 24 h UP, 24 h UA, 24 h UMA, KW, KI, BG, TC, TG, and MGV were all less than 0, while the 95% CIs for ALT included 0. This did not significantly alter the overall effect estimates, confirming the robustness of the results (Supplementary Material S3).

### 3.6 Subgroup analysis

As there were limited studies reporting markers related to protective mechanisms, no subgroup analysis was performed for this type of outcome. When the heterogeneity between studies was large, and at least 2 studies were included in each subgroup, the outcome was analyzed according to the preset subgroup perspective to investigate the causes of heterogeneity.

#### 3.6.1 Species

Species subgroup analysis of Scr and BG revealed that TGs reduced Scr levels in both rat and mouse groups, as well as BG levels in the rat group, but did not reduce BG levels in the mouse group. This indicated that species were not the source of heterogeneity for both (Table 3; Supplementary Materials S4.1, 4.2).

**TABLE 3:** Subgroup analysis of species.

Outcomes	Subgroup differences	Rats				Mice			
	*P*	N	SMD [95% CI]	*I* ^*2*^	*P*	N	SMD [95% CI]	*I* ^*2*^	*P*
Scr	0.63	47	−1.87 [−2.29, −1.46]	87%	<0.001	2	−2.32 [−4.11, −0.53]	88%	0.01
BG	0.95	43	−1.22 [−1.59, −0.84]	85%	<0.001	2	−1.10 [−4.52, 2.33]	97%	0.53

#### 3.6.2 Sex

In the sex subgroup analysis, the subgroup results of Scr, KW, and KI were consistent with the overall results, which were all reduced by TGs. Regarding Scr, the heterogeneity in the male group remained unchanged, while that in the female group became 0%, indicating that sex caused the heterogeneity (*P* = 0.003). The analysis of KW and KI suggested that sex was not the source of heterogeneity. For BUN and BG, TGs were ineffective in the female group, suggesting that sex is a source of heterogeneity for BG (*P* < 0.001) but not for BUN. Moreover, sex did not result in heterogeneity for ALT (Table 4; Supplementary Materials S4.3–4.8).

**TABLE 4:** Subgroup analysis of sex.

Outcomes	Subgroup differences	Male				Female			
	*P*	N	SMD [95% CI]	*I* ^*2*^	*P*	N	SMD [95% CI]	*I* ^*2*^	*P*
Scr	**0.003**	43	−1.88 [−2.31, −1.45]	87%	<0.001	3	−0.71 [−1.36, −0.05]	0%	0.03
BUN	0.24	42	−1.99 [−2.42, −1.56]	87%	<0.001	3	−0.90 [−2.69, 0.90]	79%	0.33
KW	0.31	9	−1.03 [−1.45, −0.61]	52%	<0.001	2	−1.65 [−2.76, −0.54]	0%	0.004
KI	0.51	26	−1.95 [−2.49, −1.41]	87%	<0.001	2	−2.50 [−4.03, −0.97]	19%	0.001
BG	**<0.001**	39	−1.29 [−1.70, −0.88]	86%	<0.001	3	0.28 [−0.36, 0.91]	0%	0.39
ALT	0.053	15	−0.04 [−0.49, 0.42]	75%	0.87	2	−1.11 [−2.09, −0.13]	0%	0.03

Bold text indicates statistically significant differences between subgroups.

#### 3.6.3 Type of DM

Subgroup analysis based on DM type revealed that the subgroup results of Scr, BUN, 24 h UP, KW, KI, BG, TC, TG, ALT, and MGV were consistent with the overall results. For TG, the T1DM group showed a greater reduction in TG levels than the T2DM group, and the heterogeneity of the two groups was decreased, indicating that DM type may be a source of heterogeneity (*P* = 0.03). Analysis of 24h UMA found that DM type was not the source of heterogeneity (Table 5; Supplementary Materials S4.9–4.19).

**TABLE 5:** Subgroup analysis of DM type.

Outcomes	Subgroup differences	T1DM				T2DM			
	*P*	N	SMD [95% CI]	*I* ^*2*^	*P*	N	SMD [95% CI]	*I* ^*2*^	*P*
Scr	0.83	28	−1.85 [−2.36, −1.35]	84%	<0.001	21	−1.94 [−2.61, −1.28]	90%	<0.001
BUN	0.55	27	−1.86 [−2.34, −1.37]	83%	<0.001	21	−2.11 [−2.80, −1.43]	89%	<0.001
24h UP	0.74	16	−3.81 [−4.55, −3.08]	77%	<0.001	13	−3.62 [−4.49, −2.75]	83%	<0.001
24h UMA	0.79	2	−6.03 [−17.43, 5.37]	93%	0.30	5	−4.47 [−6.85, −2.10]	94%	<0.001
KW	0.87	9	−1.15 [−1.60, −0.71]	46%	<0.001	3	−1.24 [−2.16, −0.31]	75%	0.009
KI	0.40	18	−1.79 [−2.51, −1.07]	89%	<0.001	11	−2.18 [−2.75, −1.62]	71%	<0.001
BG	0.15	26	−1.48 [−2.04, −0.92]	88%	<0.001	19	−0.92 [−1.42, −0.42]	83%	<0.001
TC	0.60	5	−1.62 [−2.84, −0.41]	83%	0.009	7	−1.24 [−1.96, −0.52]	79%	<0.001
TG	**0.03**	5	−2.31 [−3.18, −1.44]	59%	<0.001	7	−1.19 [−1.74, −0.65]	66%	<0.001
ALT	0.92	7	−0.12 [−0.78, 0.54]	68%	0.72	10	−0.17 [−0.79, 0.45]	80%	0.59
MGV	0.97	4	−2.37 [−3.62, −1.12]	78%	<0.001	3	−2.34 [−2.96, −1.71]	0%	<0.001

Bold text indicates statistically significant differences between subgroups.

#### 3.6.4 Number of study arms

In the subgroup analysis of number of study arms, TGs lowered Scr, BUN, 24 h UP, 24 h UA, KW, KI, BG, TC, TG, and MGV levels in both the double-arm and multi-arm groups. Among them, compared with the multi-arm group, TGs could more effectively lower the BG levels in the double-arm group, indicating that the number of study arms led to heterogeneity (*P* = 0.02). The subgroup results of 24 h UMA and ALT were inconsistent with the overall results. Analysis indicated that this factor did not result in heterogeneity of 24 h UMA. The results of ALT in the multi-arm group showed statistical differences, and heterogeneity was significantly reduced, suggesting that this factor caused heterogeneity (*P* = 0.02) (Table 6; Supplementary Materials S4.20–4.31).

**TABLE 6:** Subgroup analysis of number of study arms.

Outcomes	Subgroup differences	Double-arm studies				Multi-arm studies			
	*P*	N	SMD [95% CI]	*I* ^*2*^	*P*	N	SMD [95% CI]	*I* ^*2*^	*P*
Scr	0.055	30	−2.23 [−2.76, −1.69]	81%	<0.001	19	−1.44 [−2.04, −0.85]	90%	<0.001
BUN	0.29	28	−2.18 [−2.76, −1.60]	82%	<0.001	20	−1.74 [−2.31, −1.16]	90%	<0.001
24h UP	0.38	21	−3.55 [−4.23, −2.88]	77%	<0.001	8	−4.09 [−5.07, −3.11]	84%	<0.001
24h UA	0.27	2	−3.30 [−5.35, −1.26]	40%	0.002	4	−2.03 [−2.94, −1.13]	82%	<0.001
24h UMA	0.79	2	−6.03 [−17.43, 5.37]	93%	0.30	5	−4.47 [−6.85, −2.10]	94%	<0.001
KW	0.63	8	−1.11 [−1.67, −0.54]	50%	<0.001	4	−1.31 [−1.89, −0.72]	66%	<0.001
KI	0.40	16	−2.19 [−2.92, −1.47]	80%	<0.001	13	−1.76 [−2.46, −1.05]	90%	<0.001
BG	**0.02**	29	−1.64 [−2.22, −1.05]	86%	<0.001	16	−0.71 [−1.20, −0.23]	86%	0.004
TC	0.62	6	−1.58 [−2.53, −0.63]	70%	0.001	6	−1.25 [−2.12, −0.38]	87%	0.005
TG	0.46	6	−1.82 [−2.53, −1.11]	46%	<0.001	6	−1.42 [−2.21, −0.63]	84%	<0.001
ALT	**0.02**	9	−0.81 [−1.69, 0.07]	79%	0.07	8	0.35 [0.02, 0.67]	38%	0.04
MGV	0.98	5	−2.36 [−3.35, −1.36]	71%	<0.001	2	−2.34 [−3.04, −1.64]	0%	<0.001

Bold text indicates statistically significant differences between subgroups.

#### 3.6.5 Intervention duration

Subgroup Analysis of intervention duration showed that subgroup results of most outcomes were consistent with the overall results. This factor could not explain the heterogeneity of Scr, BUN, and BG. For 24 h UP and KI, longer intervention duration led to a greater reduction in 24 h UP and KI, indicating that this factor was the source of heterogeneity (*P* = 0.007, 0.007). ALT levels were elevated in the long intervention duration subgroup, and heterogeneity in this subgroup disappeared, suggesting that this factor caused the heterogeneity (*P* < 0.001) and long duration led to liver damage (Table 7; Supplementary Materials S4.32–4.37).

**TABLE 7:** Subgroup analysis of intervention duration.

Outcomes	Subgroup differences	Intervention duration ≤ 8w				Intervention duration > 8w			
	*P*	N	SMD [95% CI]	*I* ^*2*^	*P*	N	SMD [95% CI]	*I* ^*2*^	*P*
Scr	0.42	37	−1.78 [−2.22, −1.35]	85%	<0.001	12	−2.25 [−3.28, −1.22]	90%	<0.001
BUN	0.84	37	−1.99 [−2.46, −1.51]	87%	<0.001	11	−1.90 [−2.67, −1.13]	82%	<0.001
24h UP	**0.007**	20	−3.25 [−3.77, −2.72]	71%	<0.001	9	−5.47 [−7.00, −3.95]	88%	<0.001
KI	**0.007**	23	−1.51 [−1.94, −1.09]	77%	<0.001	6	−3.67 [−5.17, −2.16]	88%	<0.001
BG	0.07	32	−1.39 [−1.90, −0.88]	89%	<0.001	13	−0.78 [−1.21, −0.34]	64%	<0.001
ALT	**<0.001**	14	−0.38 [−0.88, 0.11]	71%	0.13	3	0.78 [0.41, 1.16]	0%	<0.001

Bold text indicates statistically significant differences between subgroups.

### 3.7 Dose-effect-toxicity analysis

One multi-arm study was not suitable for splitting due to the small sample size of the control group, so it was excluded from the analysis (He, 2020). For Scr, BUN, and 24 h UP, the subgroup results were consistent with the overall results. Scr and BUN levels decreased the most in the high-dose group, followed by the medium-dose group, and the least in the low-dose group, suggesting that TGs may reduce both in a dose-dependent manner. There was no significant difference in the decrease of 24 h UP among the different doses. Regarding toxicity indicators, three doses did not affect ALT levels, and low and medium doses did not affect AST and WBC levels, but high doses could significantly increase AST levels and reduce WBC levels, suggesting that high doses cause liver damage and hematological toxicity. Moreover, this also supplemented the dose as a source of heterogeneity for Scr and BUN (*P* = 0.001, 0.048). Overall, treatment of DN with medium dose TGs strikes a balance between efficacy and safety (Table 8).

**TABLE 8:** Dose-effect-toxicity relationship.

Outcomes	Subgroup differences	Overall			Low dose			Medium dose			High dose		
	*P*	SMD [95% CI]	*I* ^*2*^	*P*	SMD [95% CI]	*I* ^*2*^	*P*	SMD [95% CI]	*I* ^*2*^	*P*	SMD [95% CI]	*I* ^*2*^	*P*
Scr	**0.001**	−1.80 [−2.15, −1.46]	81%	<0.001	−0.99 [−1.42, −0.56]	61%	<0.001	−1.90 [−2.68, −1.13]	83%	<0.001	−2.29 [−2.85, −1.73]	83%	<0.001
BUN	**0.048**	−1.92 [−2.26, −1.58]	79%	<0.001	−1.31 [−1.84, −0.79]	72%	<0.001	−2.10 [−2.81, −1.38]	81%	<0.001	−2.19 [−2.70, −1.68]	80%	<0.001
24h UP	0.130	−4.36 [−5.03, −3.68]	84%	<0.001	−3.29 [−4.46, −2.11]	84%	<0.001	−4.84 [−6.50, −3.18]	84%	<0.001	−4.73 [−5.65, −3.81]	81%	<0.001
ALT	0.733	−0.02 [−0.36, 0.33]	59%	0.931	−0.09 [−0.68, 0.51]	63%	0.772	−0.15 [−0.81, 0.52]	64%	0.668	0.18 [−0.42, 0.79]	55%	0.551
AST	0.156	0.21 [−0.05, 0.46]	0%	0.110	−0.13 [−0.62, 0.37]	0%	0.615	0.15 [−0.28, 0.57]	0%	0.497	0.49 [0.08, 0.90]	0%	0.018
WBC	0.126	−0.11 [−0.38, 0.17]	0%	0.450	0.14 [−0.29, 0.57]	0%	0.527	−0.07 [−0.51, 0.37]	0%	0.751	−0.61 [−1.20, −0.03]	16%	0.040

Bold text indicates statistically significant differences between subgroups.

### 3.8 Meta-regression analysis

The results of meta-regression analysis showed that the modeling method, manufacturer of TGs, methodological quality score, sample size, and publication year had no statistically significant effect on heterogeneity, indicating that they were not sources of heterogeneity. In contrast, reference type was associated with potential heterogeneity for Scr (*P* = 0.005), BUN (*P* = 0.004), TC (*P* = 0.046), and TG (*P* = 0.009). STZ dose was a source of heterogeneity for BUN (*P* = 0.019), and strain was a source of heterogeneity for 24 h UP (*P* = 0.0185) (Supplementary Material S5).

### 3.9 Publication bias

For publication bias, the funnel plot can quickly assess symmetry, but it is subjective. Egger’s test quantifies objectively, but its effectiveness is limited by the number of studies. Combining the two methods can improve the accuracy. Outcomes with 10 or more studies included were Scr, BUN, 24h UP, KW, KI, BG, TC, TG, ALT, AST, and ALB. Among them, the funnel plots of Scr, BUN, 24h UP, KI, BG, and ALT were asymmetric, suggesting that these outcomes may have publication bias. Subsequent Egger’s tests further confirmed the presence of publication bias (*P* < 0.001, <0.001, <0.001, = 0.005, <0.001, = 0.004). While the funnel plots of KW, TC, TG, AST, and ALB were symmetrical and the Egger’s tests showed no statistical difference (*P* = 0.556, 0.219, 0.188, 0.777, 0.401), indicating that they had no publication bias (Supplementary Materials S6.1, S6.2).

The trim-and-fill method did not identify any missing studies for Scr, BUN, 24 h UP, or BG, and the pooled effect sizes remained unchanged before and after adjustment, indicating that the results were not affected by publication bias. For KI, five missing studies were identified. After adjustment, the pooled SMD changed from −1.97 [−2.46, −1.47] to −2.43 [−3.01, −1.86], and the significance of the effect size remained unchanged, suggesting that the results are robust. Additionally, for ALT, two missing studies were identified. After adjustment, the pooled SMD changed from −0.14 [−0.58, 0.30] to −0.33 [−0.79, 0.13], and the effect size remained non-significant, confirming the robustness of the results (Supplementary Materials S6.3, S6.4).

### 3.10 Evidence certainty

The results regarding the evidence quality showed that, of the 18 outcomes assessed, TC, TG, AST, ALB, WBC, RBC, PLT, and MGA were rated as moderate (44.44%), while Scr, BUN, 24 h UP, 24 h UA, KW, KI, BG, ALT, and MGV were rated as low (50.00%), and 24 h UMA was rated as very low (5.56%). Regarding the reasons for downgrading, the risk of bias was the main factor. All outcomes were downgraded because it was not stated whether allocation concealment and blinding were employed. The heterogeneity of 24 h UA, KW, and MGV was high, and the sources were not found, resulting in serious inconsistency. Furthermore, the heterogeneity of 24 h UMA exceeded 90% for unknown reasons, leading to a very serious inconsistency. Some outcomes were downgraded by one level due to publication bias (Supplementary Material S7).

## 4 Discussion

### 4.1 Main results

By combining 55 experimental studies, the results showed that compared with the control group, TGs significantly reduced Scr, BUN, 24 h UP, 24 h UA, 24 h UMA, KW, KI, BG, TC, TG, MGA, and MGV levels while increasing ALB levels, but did not significantly affect ALT, AST, WBC, RBC, or PLT levels. Moreover, TGs modulated the expression of most markers related to oxidative stress, inflammation, renal fibrosis, podocyte injury, and autophagy. Sensitivity analysis confirmed the robustness of the results. Subgroup analysis revealed that species was not considered a source of heterogeneity, possibly due to the small number of mouse studies included. Sex could explain the heterogeneity of Scr and BG. Type of DM was a source of heterogeneity for TG. Moreover, number of study arms led to the heterogeneity of BG and ALT. Intervention duration was the source of heterogeneity for 24 h UP, KI, and ALT. Dose-effect-toxicity analysis supplemented the dose as a source of heterogeneity for Scr and BUN. Meta-regression analysis showed that reference type, STZ dose, and strain were sources of heterogeneity. Egger’s test found that there was publication bias in some outcomes. However, the trim-and-fill method indicated that the results are robust. The methodological quality was moderate to low, and the evidence quality was moderate to very low.

### 4.2 The renal protective mechanisms of TGs

**FIGURE 13:**
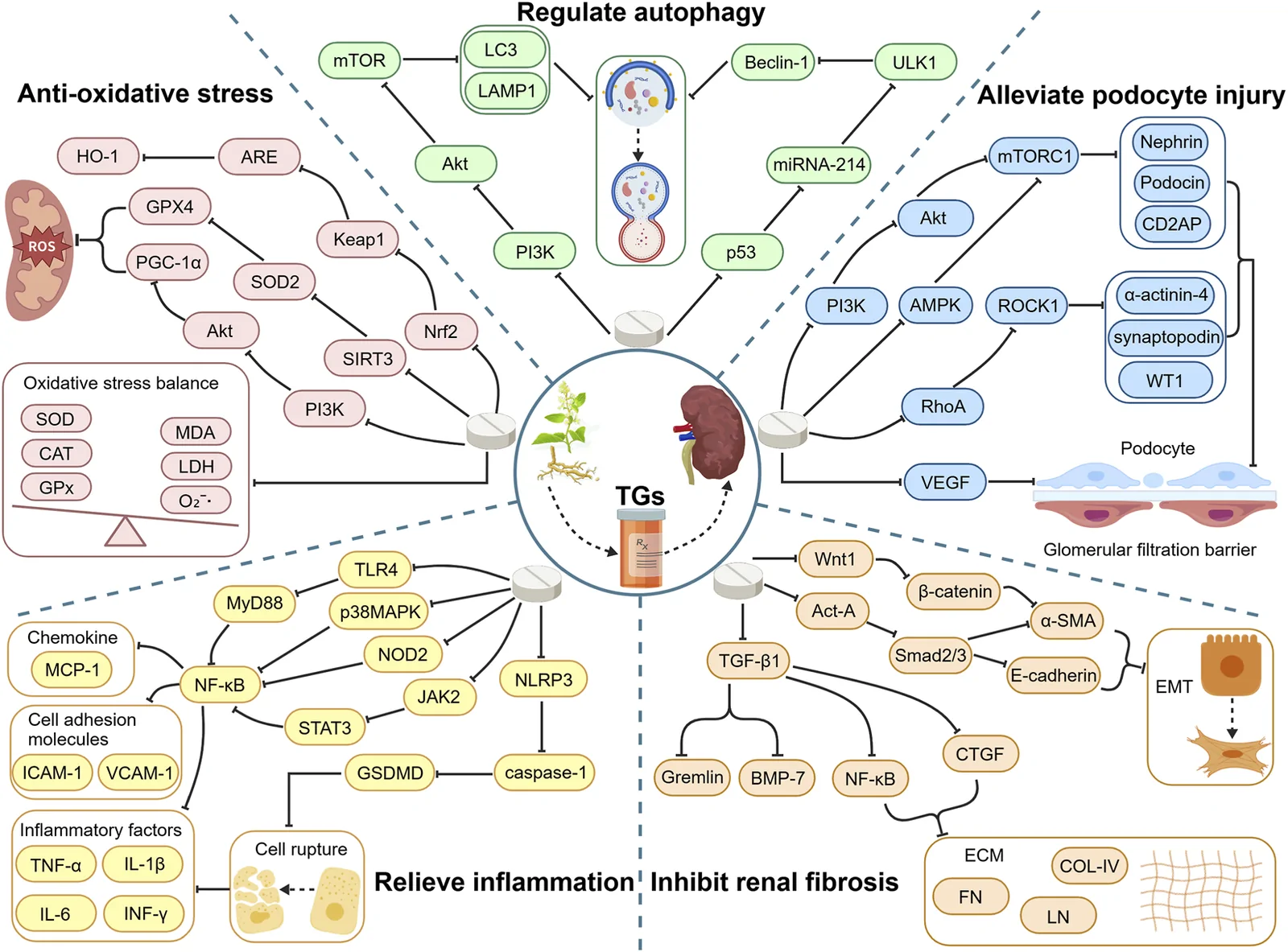
Protective mechanisms of TGs in treating DN.

The protective mechanisms of TGs in treating DN mainly involve five aspects: anti-oxidative stress, relieving inflammation, inhibiting renal fibrosis, alleviating podocyte injury, and regulating autophagy (Jiang et al., 2025) (Figure 13). Furthermore, TGs can also suppress immune response, improve glucose and lipid metabolism, and prevent apoptosis.

#### 4.2.1 Anti-oxidative stress

The extent of oxidative stress depends on the balance between antioxidant and pro-oxidant factors, which is a key mechanism for the progression of DN. The Nrf2/Keap1/ARE pathway is a crucial antioxidant stress pathway. One study found that TGs alleviate renal damage by activating this pathway to upregulate the expression of Nrf2 and HO-1 and downregulate the expression of Keap1 (Yang et al., 2023). Mitochondria are recognized as the main source of reactive oxygen species, and TGs effectively restore mitochondrial redox homeostasis and alleviate ferroptosis by upregulating the SIRT3/SOD2/GPX4 pathway (Zhang et al., 2026). Additionally, PGC-1α can protect mitochondria, and TGs may exert antioxidant effects by inhibiting the PI3K/Akt pathway and promoting PGC-1α expression (Yu et al., 2021). In STZ-induced DN rats, TGs decreased lactate dehydrogenase activity and increased SOD activity (Ding et al., 2017). Another study found that TGs can increase serum CAT and renal GPx activity, while reducing serum superoxide anion and renal MDA levels (Zhang, 2014).

#### 4.2.2 Relieve inflammation

TGs relieve inflammation by regulating multiple signaling pathways, particularly the NF-κB inflammatory core pathway, thereby preventing the release of inflammatory factors, chemokines, and cell adhesion molecules, as well as the recruitment of inflammatory cells. In SD rats, TGs can reduce glomerular macrophage infiltration, suppress the overexpression of renal inflammatory factors, and block the activation of the p38MAPK and NF-κB pathways, thereby improving glomerulosclerosis (Wu et al., 2017). Concurrently, TGs also alleviated tubulointerstitial lesions in DN, which was associated with inhibiting the TLR4/MyD88/NF-κB pathway and preventing inflammation mediated by TNF-α, IL-1β, and VCAM-1 (Ma, 2015). In the kidneys of T2DM rats, TGs can inhibit the JAK2/STAT3 pathway, upregulate SOCS1, and downregulate downstream molecules including ICAM-1, IL-6, INF-γ, and MCP-1 (Li, 2016a; Meng et al., 2020). Furthermore, TGs also suppressed the activity of the NOD2/NF-κB pathway (Chen et al., 2023). The activation of pyroptosis is the trigger of the inflammatory response, and TGs inhibit the release of inflammatory factors after cell rupture by blocking the NLRP3/caspase-1/GSDMD pathway (Song et al., 2023).

#### 4.2.3 Inhibit renal fibrosis

Renal fibrosis is an important pathological feature of DN. The anti-fibrotic effects of TGs involve blocking TGF-β1 and Wnt1/β-catenin pathways, improving levels of fibrosis markers, and thereby inhibiting extracellular matrix (ECM) proliferation and epithelial-mesenchymal transition (EMT). Specifically, TGs dose-dependently inhibit TGF-β1/NF-κB pathway-mediated ECM synthesis, including COL-IV, FN, and LN (Yang, 2016). CTGF is a key downstream mediator of TGF-β1-induced ECM synthesis, and TGs can reduce CTGF levels (Zhang J. et al., 2014). Overexpression of TGF-β1 in DN upregulates Gremlin, which antagonizes the antifibrotic effects of BMP-7, while TGs can restore this imbalance (Zhang et al., 2012). In the DN model induced by HFD combined with STZ, TGs reversed podocyte EMT by inhibiting the Wnt1/β-catenin pathway and reducing α-SMA (Guo et al., 2025). Other studies have found that TGs help downregulate Act-A and Smad2/3 and upregulate E-cadherin, thereby delaying the transformation of tubulointerstitial cells into myofibroblasts and slowing fibrosis (Zhang W. et al., 2014; Zhao et al., 2014).

#### 4.2.4 Alleviate podocyte injury

Podocytes maintain the integrity of the glomerular filtration barrier through the slit diaphragm and cytoskeleton. Within the glomerulus, VEGF is primarily expressed in podocytes, and its overexpression leads to increased permeability of the filtration barrier and promotes proteinuria. TGs can increase the expression of nephrin and podocin and reduce VEGF levels, thus preserving the slit diaphragm (Li et al., 2016b). TGs can also increase the expression of podocyte structural molecules CD2AP, the binding level of Nephrin and Neph1, and alleviate podocyte injury by regulating the activity of the PI3K/Akt/mTORC1 and AMPK/mTORC1 pathway (Mao, 2016). Moreover, TGs inhibit RhoA/ROCK1, increase renal α-actinin-4, synaptopodin, and WT1 levels, and remodel the podocyte cytoskeleton, ultimately reducing podocyte loss (Guo et al., 2025).

#### 4.2.5 Regulate autophagy

Inhibition of autophagy function will promote DN. Beclin-1 and LC3 are both critical autophagy regulators that participate in all stages of autophagy. One study suggests that the autophagy state is regulated by the dose of TGs. 6.25 mg/kg/day of TGs can inhibit the PI3K/Akt/mTOR pathway, increase LAMP1 and LC3 protein expression, and promote autophagy; however, when the dose exceeds 12.5 mg/kg/day, autophagy activity decreases with increasing dose (He, 2020). Another study found that the mechanisms by which TGs improve renal function include suppressing the mTOR pathway to activate autophagy, enhancing cellular self-cleaning capacity, and alleviating oxidative stress (Zhang et al., 2020). Additionally, TGs significantly increased renal ULK1 and Beclin-1 expression and the LC3-II/LC3-I ratio, downregulated p53 expression and miR-214 transcription, and restored autophagy by regulating the p53/miR-214/ULK1 axis (Zhang et al., 2025). In summary, TGs promote autophagy at routine clinical doses.

#### 4.2.6 Other mechanisms

TGs also protect DN by inhibiting immunity, improving glucose and lipid metabolism, and preventing apoptosis. The complement system, immune cells, and antibodies jointly regulate the immune response. Specifically, TGs can inhibit the lectin pathway of complement activation, reduce the infiltration of inflammatory cells and levels of inflammatory cytokines, thus alleviating the immune-inflammatory response (Zhao et al., 2017). One experiment found that different doses of TGs help regulate the Th1/Th2 cell ratio in the blood (Kong, 2013). For antibodies, levels of IgA, IgG, and IgM in the DN rat model were significantly lowered after TGs intervention (Li and Yan, 2015). Regarding glucose metabolism, the renal protective effect of TGs may be attributed to the inhibition of the RAGE/NF-κB pathway (Xu et al., 2018). Furthermore, one study suggests that TG is a potential therapeutic target for DN, as TGs promote TG catabolism by upregulating renal adipose triglyceride lipase (Zhang et al., 2022). Regarding apoptosis, TGs can reduce Caspase-3 activity and the expression of inflammatory factors (Ding et al., 2017).

### 4.3 Efficacy and safety

The duration and dose of TGs are the two key factors determining their efficacy and safety (Ru et al., 2019). A clinical meta-analysis found that the incidence of adverse reactions was higher with TGs combined with ACEI/ARB than with ACEI/ARB alone (Yao et al., 2024). This finding differs from the overall pooled results for safety outcomes in this study, primarily due to three differences in the study population, the intervention, and the types of safety outcomes. Clinical studies, which involved human subjects and used combination therapy for 2–24 weeks, were able to capture human-specific and long-term adverse reactions such as reproductive toxicity, gastrointestinal reactions, bone marrow suppression, and menstrual disorders. In contrast, the animal study used animals receiving TGs alone for 4–16 weeks, and reported only liver function and blood routine parameters. In this study, although overall pooled results indicate that TGs have good efficacy and safety, intervention time exceeding 8 weeks is associated with hepatotoxicity, while doses above 9 mg/kg/day are linked to both hepatotoxicity and hematological toxicity. This indicates that TGs present potential safety risks, and the intervention time and dose should be strictly limited.

Notably, longer duration did not significantly affect the reduction in Scr and BUN, while the reduction in 24 h UP was greater; higher doses led to greater reduction in Scr and BUN, but did not significantly affect the reduction in 24 h UP. This suggests that appropriate doses and treatment cycles can be combined based on individual patient outcomes and pathological symptoms to enhance efficacy. Additionally, one study found that the reproductive toxicity induced by TGs can be reduced by combining with small molecule drugs, TCM, and TCM prescriptions (Niu et al., 2025). Monomer separation, structural optimization, prodrug engineering, and targeted delivery can also reduce the inherent toxicity of diterpenoids (Jin et al., 2026).

The administered doses of TGs range from 0.1 to 1800 mg/kg/day, representing a very wide span. The main reasons are as follows: First, different dose levels serve different experimental purposes. The very low doses are used to explore minimal drug effects, medium doses simulate clinically relevant therapeutic concentrations, and extremely high doses are applied in toxicity and limit-exposure mechanism studies. Second, significant interspecies differences exist in drug metabolism between experimental animals and humans, and animals tolerate much higher doses of TGs than humans do. Third, a broad dose range helps systematically characterize the dose-effect-toxicity relationship and avoids missing non-monotonic effects or threshold phenomena.

The optimal dose (6–9 mg/kg/day) in animals, converted using the body surface area method, is equivalent to the routine clinical dose (1–1.5 mg/kg/day), and the optimal treatment duration (≤8 weeks) does not exceed the clinically recommended 3 months (Li et al., 2021), providing an experimental reference for precise clinical dosing. Individualized adjustment of treatment regimens based on pharmacodynamic differences in dose and duration can targetedly improve renal function parameters. At the clinical translation level, the pharmacodynamic patterns identified through wide-dose gradient studies, combined with the aforementioned toxicity mitigation strategies, will provide new directions for overcoming toxicity limitations and broadening clinical applications. However, significant translational challenges remain: animal experiments cannot fully replicate the complex metabolic environment in humans, and individualized criteria for dose-duration adaptation have not yet been standardized. It is recommended that clinical trials investigating dose-duration interactions be conducted next to validate safety and efficacy.

### 4.4 Innovations

This study is the first meta-analysis of the effects of TGs on DN animal models, comprehensively incorporating the outcomes of efficacy, safety, and five types of mechanisms of action. Data processing in multi-arm studies primarily focuses on pooling different experimental groups, supplemented by splitting common control groups to avoid losing the impact of dose on efficacy and toxicity, and to assess whether dose was a source of heterogeneity. Moreover, the pooled results of the outcomes (Scr, BUN, 24 h UP, ALT, AST, and WBC) analyzed by these two methods were consistent, further confirming the reliability of the results. Through subgroup analysis, the relationship between dose-time and efficacy-toxicity was preliminarily explored, facilitating the extrapolation of the optimal dose and duration from animal experiments to clinical studies. Based on this, more targeted clinical trials can be designed to deeply explore and validate the optimal dose and duration of TGs, ultimately providing insights for enhancing efficacy and reducing toxicity. Furthermore, we have comprehensively summarized the protective mechanisms of TGs in treating DN.

### 4.5 Limitations and implications

Regarding the limitations and implications, five aspects merit attention.

First, the methodological quality of the included studies was insufficient. Although many studies were included, their quality scores varied widely, and there was a lack of high-quality studies. There is significant room for improving allocation concealment and blinding in preclinical studies. Therefore, authors are encouraged to pre-register their animal experiment protocols to enhance transparency and reduce publication bias, and to complete the ARRIVE 2.0 checklist when submitting their manuscripts. Reviewers can use the results of the bias risk assessment as an important basis for their review.

Second, there is an imbalance in the selection and reporting of outcomes. Efficacy outcomes are reported more frequently than safety outcomes, and the number of studies reporting safety outcomes accounts for less than one-third of the total number of included studies, resulting in limited evidence to support conclusions regarding safety. Moreover, while researchers focused far more on liver function than on blood routine, hematological toxicity cannot be ignored. For TGs, safety metrics should be reported as one of the core outcomes. Authors are encouraged to publish papers that include explicit toxicity results, even when the drug demonstrates significant efficacy. Reporting toxicity does not negate the therapeutic value of the drug; rather, it provides important safety window information for clinical translation.

Third, most outcomes exhibited high heterogeneity. This was partly attributed to duration, TGs dose, reference type, sex, number of study arms, type of DM, STZ dose, and strain. Some outcomes still showed residual heterogeneity after subgroup analysis and meta-regression, suggesting the presence of unidentified confounding factors. Therefore, the results should be interpreted with caution. Apart from the limited number of studies and insufficient statistical power for a few outcomes, the remaining heterogeneity may stem from inconsistencies in animal housing conditions, baseline severity of renal injury, purity of TGs, reagent manufacturers, and detection methods, as well as complex interactions among multiple factors. Standardizing animal experimental conditions and reporting details, and controlling for confounding factors such as baseline differences and drug purity, are important approaches to reducing heterogeneity.

Fourth, the methods used to analyze dose-effect-toxicity relationships were relatively conventional. We employed only subgroup analysis for preliminary exploration and did not conduct meta-regression analysis to further interpret the dose-response patterns. There were three main reasons for this: (1) Most animal studies used only one or two dose levels, lacking a continuous dose gradient, which made it impossible to fit a stable dose-response curve. (2) Confounding factors such as animal strains, modeling methods, DM types, and intervention duration interfered with the trends between dose and effect. (3) The overall sample sizes of individual studies were small, reducing the testing power of meta-regression analysis and making it difficult to identify potential dose-response relationships. Further standardized animal experiments with multiple dose gradients are needed to thoroughly investigate the dose-effect-toxicity relationship.

Fifth, there is a lack of specific markers for the NF-κB signaling pathway. The included studies reported only NF-κB protein or mRNA levels without measuring phosphorylation levels, which limits the ability to accurately assess the activation status of this pathway. To more accurately assess the regulatory role of TGs on the NF-κB pathway, it is recommended that future animal studies, in addition to measuring NF-κB expression, further determine NF-κB phosphorylation levels and evaluate NF-κB nuclear translocation via immunofluorescence. This will more strongly substantiate the anti-inflammatory mechanism of TGs in DN.

## 5 Conclusion

This meta-analysis indicates that TGs have a favorable renal protective effect on DN animals, which can improve renal function, basic indicators, glucose and lipid metabolism, and renal tissue pathological changes. For safety reasons, the intervention time (≤8 weeks) and dose (6–9 mg/kg/day) should be strictly restricted to prevent potential hepatotoxicity and hematological toxicity. Its protective mechanisms mainly include anti-oxidative stress, relieving inflammation, inhibiting renal fibrosis, alleviating podocyte injury, and regulating autophagy. Given the limited methodological and evidential quality of existing studies, more high-quality animal studies are needed in the future to verify the efficacy, safety, and protective mechanism of TGs in the treatment of DN.

## Data Availability

The datasets presented in this study can be found in online repositories. The names of the repository/repositories and accession number(s) can be found in the article/Supplementary Material.

## Glossary

24 h UA: 24-h urine albumin
24 h UMA: 24-h urine microalbumin
24 h UP: 24-h urine protein
ALB: albumin
ALT: alanine aminotransferase
Akt: protein kinase B
AST: aspartate aminotransferase
BG: blood glucose
BUN: blood urea nitrogen
CAT: catalase
CI: confidence interval
Col-IV: collagen type IV
CTGF: connective tissue growth factor
DM: diabetes mellitus
DN: diabetic nephropathy
GPx: glutathione peroxidase
HFD: high-fat diet
HFSD: high-fat and high-sugar diet
ig: gavage
IL-1β: interleukin-1β
IP: intraperitoneal injection
JAK2: janus kinase 2
KI: kidney index
KW: kidney weight
LC3: microtubule-associated protein 1 light chain 3
LN: laminin
MCP-1: monocyte chemotactic protein-1
MDA: malondialdehyde
MGA: mean glomerular area
MGV: mean glomerular volume
mTOR: mammalian target of rapamycin
NF-κB: nuclear factor kappa-B
PI3K: phosphatidylinositol 3-kinase
PLT: platelets
p38MAPK: p38 mitogen-activated protein kinase
RBC: red blood cells
Scr: serum creatinine
SD: standard deviation
SD rats: Sprague-Dawley rats
SE: standard error
SMD: standardized mean difference
SOD: superoxide dismutase
STAT3: signal transducer and activator of transcription 3
STZ: streptozotocin
T1DM: type 1 diabetes mellitus
T2DM: type 2 diabetes mellitus
TC: total cholesterol
TCM: traditional Chinese medicine
TG: triglycerides
TGF-β1: transforming growth factor-β1
TGs: tripterygium glycosides
TNF-α: tumor necrosis factor-α
TIV: tail intravenous injection
α-SMA: α-smooth muscle actin
UN: unilateral nephrectomy
VCAM-1: vascular cell adhesion molecule-1
VEGF: vascular endothelial growth factor
WBC: white blood cells
WMD: weighted mean difference
Wnt1: Wnt family member 1

## References

[B1] Agarwal R. (2025). The FLOW of progress in diabetic kidney disease: semaglutide’s role in combination therapy. Diabetes Care 48 (11), 1875–1877. 10.2337/dci25-0088 41115233

[B2] Chang J. Wang C. Shen Y. You L. Guo H. (2012). Study of tripterygium glycosides in CTGF abnormal expression and the effect in the renal cortex of DM rats. Chin. J. Exp. Tradit. Med. Formulae 18 (24), 307–311. 10.13422/j.cnki.syfjx.2012.24.089

[B3] Chang B. Chen W. Zhang Y. Yang P. Liu L. Wang J. (2014). Effect of total glucosides of paeony on Wnt/β-catenin signal transduction pathway expression in kidney of diabetic rats. China J. Chin. Mater. Med. 39 (19), 3829–3835. 10.4268/cjcmm20141933 25612449

[B4] Chang S. Xu X. Xiang H. Meng W. Zheng H. Zhang H. (2021). Effects of tripterygium glycosides on the renal injury in mice with diabetic nephropathy *via* Akt/mTOR signaling pathway. Hebei Med. J. 43 (01), 25–29. 10.3969/j.issn.1002-7386.2021.01.005

[B5] Chen W. Chang B. Zhang Y. Yang P. Liu L. (2015). Effect of tripterygium glycosides on expression of hypoxia inducible factor-1α and endothelin-1 in kidney of diabetic rats. J. South. Med. Univ. 35 (04), 499–505. 10.3969/j.issn.1673-4254.2015.04.007 25907932

[B6] Chen C. Song D. Song C. Guo T. Duan F. Wang N. (2023). Effect of tripterygium wilfordii polyglycoside on diabetic nephropathy rats based on NOD2/NF-κB pathway. Acta Chin. Med. 38 (10), 2162–2167. 10.16368/j.issn.1674-8999.2023.10.349

[B7] Chi J. Xu D. Wang J. Zhang Y. (2009). The effect of Bupi Yiqi Shengyang decoction on the expression of MCP-1 mRNA in renal tissue of diabetic rats. Acta Chin. Med. Pharmacol. 37 (04), 28–32. 10.19664/j.cnki.1002-2392.2009.04.011

[B8] Chopra B. Dhingra A. K. (2021). Natural products: a lead for drug discovery and development. Phytother. Res. 35 (9), 4660–4702. 10.1002/ptr.7099 33847440

[B9] Cumpston M. Li T. J. Page M. J. Chandler J. Welch V. A. Higgins J. P. T. (2019). Updated guidance for trusted systematic reviews: a new edition of the Cochrane handbook for systematic reviews of interventions. Cochrane Database Syst. Rev. 10, ED000142. 10.1002/14651858.ED000142 31643080 PMC10284251

[B10] Ding X. Cao Y. Guo Y. Gao W. Bai Y. (2017). Effects and functional mechanism of tripterygium wilfordii polyglycoside with catalpol on diabetic nephropathy. Int. J. Clin. Exp. Med. 10 (12), 16210–16216. Available online at: https://www.embase.com/records?id=L620003069 (Accessed July 12, 2026).

[B11] Duan F. Song C. Song D. Ding Y. Ren X. Zhang X. (2023). Effect of tripterygium wilfordii polyglycoside on expression of NFAT2/COX-2 in kidney tissues of rats with diabetic nephropathy. Chin. J. Exp. Tradit. Med. Formulae 29 (05), 16–23. 10.13422/j.cnki.syfjx.20222445

[B12] Guo H. (2013). Effect of Tripterygium wilfordii Polyglucoside Tablets on Infiltration of Macrophages and Expression of NF-κB, MCP-1 in Rats with Diabetic Nephropathy. Tianjin: Tianjin Medical University. Master thesis. 10.7666/d.Y2397037

[B13] Guo S. Li S. Luo H. Wang L. Chi Y. (2025). Mechanism of tripterygium glycoside in reducing proteinuria in diabetic nephropathy rats through RhoA/ROCK1 and Wnt1/β-catenin signaling pathways. J. Radiat. Res. Appl. Sci. 18 (3), 101819. 10.1016/j.jrras.2025.101819

[B14] He K. J. (2020). Study on the dose-effect-toxicity Relationship of Tripterygium Glycosides Tablets in Diabetic Nephropathy Rats. Beijing: Beijing University of Chinese Medicine. Master thesis.

[B15] He Y. Li P. Hou P. Ni J. Yu D. Chen H. (2026). Protective mechanism of tripterygium glycosides on diabetic nephropathy based on the theory of “gut kidney axis”. Chin. J. Integr. Tradit. West. Nephrol. 27 (2), 106–112. 10.3969/j.issn.1009-587X.2026.02.005

[B16] Hooijmans C. R. Rovers M. M. de Vries R. B. M. Leenaars M. Ritskes-Hoitinga M. Langendam M. W. (2014). SYRCLE's risk of bias tool for animal studies. BMC Med. Res. Methodol. 14, 43. 10.1186/1471-2288-14-43 24667063 PMC4230647

[B17] Huang Y. R. (2015). Effects and Mechanisms of multi-glycosides of Tripterygium wilfordii Hook. for Alleviating Glomerular Hypertrophy via Inhibiting PI3K/Akt/mTOR Signaling Pathway Activity in Diabetic Nephropathy Rats. Nanjing: Nanjing University of Chinese Medicine. Master thesis.

[B18] Jiang S. Li H. Zhang L. Mu W. Zhang Y. Chen T. (2025). Generic diagramming platform (GDP): a comprehensive database of high-quality biomedical graphics. Nucleic Acids Res. 53 (D1), D1670–d1676. 10.1093/nar/gkae973 39470721 PMC11701665

[B19] Jin D. Fu Y. Zhang H. Sun Y. Xue J. Di Z. (2025). Recent advances in Tripterygium wilfordii Hook F against diabetic kidney disease: a review of preclinical and clinical evidences. Phytomed. Plus, 5(4), 100863. 10.1016/j.phyplu.2025.100863

[B20] Jin Y. Cui Y. Zhang Z. Huang C. Tong R. Ling Y. (2026). Tripterygium glycosides: recent advances in mechanisms, therapeutic applications, and safety optimization. Front. Med. (Lausanne) 13, 1728162. 10.3389/fmed.2026.1728162 41704700 PMC12907827

[B21] Kong Y. (2013). Mechanism of Tripterygium wilfordii Polyglycosides on Preventing and Treatment of Type 2 Diabetic Nephropathy by Th Cells Balance. Tianjin: Tianjin Medical University. Master thesis. 10.7666/d.Y2396462

[B22] Kong R. Wang Z. Sun Y. Zhang Q. Han Y. Qi Y. (2021). Protective effect of yam polysaccharide on renal injury in type 2 diabetic rats and its effect on Wnt/β-catenin signaling pathway. World Latest Med. Inf. 21 (92), 234–236. 10.3969/j.issn.1671-3141.2021.92.078

[B23] Li H. (2014). Effect of Tripterygium wilfordii Polyglucosides on Wnt/β-catenin Signaling Pathway in the Renal Interstitium of Diabetic Nephropathy Rats. Taiyuan: Shanxi Medical University. Master thesis. 10.7666/d.Y2660622

[B24] Li L. (2016a). The Effect of the Tripterygium wilfordii Polyglucosides and Berberine on JAK/STAT Pathway of Kidney in Diabetes Rats. Tianjin: Tianjin Medical University. Master thesis. 10.7666/d.D01142980

[B25] Li X. Yan Y. (2015). Experimental study on effect of tripterygium glycosides on rats with diabetic nephropathy. Shanxi J. Tradit. Chin. Med. 31 (04), 49–50+53. 10.3969/j.issn.1000-7156.2015.04.029

[B26] Li Y. Shen Y. Liu G. Chang J. Guo H. (2012). Protective effect and mechanism of tripterygium wilfordii polyglycoside on the kidney in experimental diabetic rats. Chin. J. Clin. Pharmacol. 28 (06), 450–452. 10.13699/j.cnki.1001-6821.2012.06.007

[B27] Li L. Zhao R. Li C. Sun B. Yang W. Ma Z. J. (2016b). Protective effects of tripterygium wilfordii polyglycosides on kidney of diabetic rats. Chin. J. Diabetes 24 (05), 459–464. 10.3969/j.issn.1006-6187.2016.05.017

[B28] Li X. Ren Y. Zhang L. Liu K. Cao Z. Meng Y. (2019). Effect of Danggui Shaoyao powder on oxidative stress in early diabetic renal injury rats. J. Li-shizhen Tradit. Chin. Med. 30 (09), 2082–2084. 10.3969/j.issn.1008-0805.2019.09.011

[B29] Li Y. Miao R. Liu Y. Zhang J. Dou Z. Zhao L. (2021). Efficacy and safety of tripterygium glycoside in the treatment of diabetic nephropathy: a systematic review and meta-analysis based on the duration of medication. Front. Endocrinol. (Lausanne) 12, 656621. 10.3389/fendo.2021.656621 33959100 PMC8095376

[B30] Liang S. Song C. Duan F. Zhang B. Zhang X. Ding Y. (2026). Mechanism of multi-glycosides of Tripterygium wilfordii in regulating autophagy in diabetes nephropathy rats through VDR/DDIT4/mTOR signaling pathway. China J. Tradit. Chin. Med. Pharm. 41 (1), 72–77.

[B31] Ling L. Chen L. Lao G. Zhang C. Lin D. (2016). Protective effects of triptergium wilfordii polyglycosides on podocyte lesions of rats with diabetic nephropathy. Chin. J. Prev. Control. Chron. Dis. 24 (07), 489–492. 10.16386/j.cjpccd.issn.1004-6194.2016.07.003

[B32] Liu J. Y. (2013). The Effect of Tripterygium wilfordii Polyglucoside on the TGF-β1 and FN in Rats with Diabetic Nephropathy. Tianjin: Tianjin Medical University. Master thesis. 10.7666/d.Y2396278

[B33] Liu G. Shen Y. You L. Song W. Lu K. Li Y. (2014a). Tripterygium wilfordii polyglycoside suppresses inflammatory cytokine expression in rats with diabetic nephropathy. Chin. J. Cell. Mol. Immunol. 30 (07), 721–724. 10.13423/j.cnki.cjcmi.007006 25001937

[B34] Liu G. Lu K. You L. Guo H. Li Y. (2014b). Effect of tripterygium wilfordii polyglycosidum on connective tissue growth factor in liver in diabetic nephropathy rats. Chin. J. Clin. Pharmacol. 30 (07), 603–606. 10.13699/j.cnki.1001-6821.2014.07.014

[B35] Liu Y. Bai H. Liu J. Chu Y. Yuan X. (2017). Protective effect of tripterygium glycosides on renal inflammatory injury in diabetic nephropathy rats. Guangming J. Chin. Med. 32 (11), 1577–1579. 10.3969/j.issn.1003-8914.2017.11.022

[B36] Liu P. Zhang J. Wang Y. Shen Z. Wang C. Chen D. Q. (2021). The active compounds and therapeutic target of Tripterygium wilfordii hook. f. in attenuating proteinuria in diabetic nephropathy: a review. Front. Med. (Lausanne) 8, 747922. 10.3389/fmed.2021.747922 34621768 PMC8490618

[B37] Lu K. Liu G. Shen Y. Song W. Li Y. (2015). Protective effect of tripterygium wilfordii polyglycoside on inflammatory injury kidney of rats with diabetic nephropathy. Pharmacol. Clin. Chin. Mater. Med. 31 (03), 93–96. 10.13412/j.cnki.zyyl.2015.03.027

[B38] Lv H. Liu L. (2009). Influence of tripterygium wilfordii glycoside tablet on the expression of connective tissue growth factor in diabetic nephropathy rats. Chin. J. Integr. Tradit. West. Nephrol. 10 (04), 312–314. 10.3969/j.issn.1009-587X.2009.04.008

[B39] Ma Z. J. (2015). The Effect and Mechanism of the Triptygium Glycosides on Diabetic Renal Tubulointerstitial Lesions. Doctor Thesis. Tianjin: Tianjin Medical University.

[B40] Mao Z. M. (2016). Effects and Mechanisms of Multiglycoside of Tripterygium wilfordii Hook. F. Improving Podocyte Injury in Diabetic Nephropathy Rats by Regulating the Activation of mTORC1 Signaling Pathways. Nanjing: Nanjing University of Chinese Medicine. Master thesis.

[B41] Martinez Leon V. Hilburg R. Susztak K. (2026). Mechanisms of diabetic kidney disease and established and emerging treatments. Nat. Rev. Endocrinol. 22 (1), 21–35. 10.1038/s41574-025-01171-3 40935879 PMC13714763

[B42] Meng B. Yan C. Cai S. (2020). Protective effect of tripterygium glycosides on kidney tissue of rats with diabetic nephropathy. Chin. J. Clin. Pharmacol. 36 (10), 1270–1273. 10.13699/j.cnki.1001-6821.2020.10.027

[B43] Niu Z. Zhang H. Cai C. Yang T. Ma T. Xu D. (2025). The mechanisms of tripterygium glycosides-induced reproductive toxicity and detoxification strategies. Reprod. Toxicol. 132, 108830. 10.1016/j.reprotox.2025.108830 39778665

[B44] Page M. J. McKenzie J. E. Bossuyt P. M. Boutron I. Hoffmann T. C. Mulrow C. D. (2021). The PRISMA 2020 statement: an updated guideline for reporting systematic reviews. PLoS Med. 18 (3), e1003583. 10.1371/journal.pmed.1003583 33780438 PMC8007028

[B45] Ru Y. Luo Y. Zhou Y. Kuai L. Sun X. Xing M. (2019). Adverse events associated with treatment of Tripterygium wilfordii hook F: a quantitative evidence synthesis. Front. Pharmacol. 10, 1250. 10.3389/fphar.2019.01250 31780926 PMC6851843

[B46] Shen P. Wang J. Zhao Y. (2014). Effects of tripterygium glycosides on the expression of Desmin and Synaptopodin in the kidney of diabetic nephropathy rats. Mod. Med. J. China 16 (01), 21–23.

[B47] Song C. Hou X. Xue L. Ren R. Yang X. (2015). Effects of triperygium wilfordii multiglucoside on WT1 in rat renal tissue with diabetic nephropathy. China J. Tradit. Chin. Med. Pharm. 30 (12), 4472–4474.

[B48] Song C. Song D. Ren X. Zhai W. Ding Y. (2020). Effects of tripterygium wilfordii multiglucoside tablets on expressions of RhoA and ROCK1 in renal tissue of rats with diabetic nephropathy. Chin. Arch. Tradit. Chin. Med. 38 (08), 166–169+280. 10.13193/j.issn.1673-7717.2020.08.040

[B49] Song C. Song D. Jia P. Duan F. Ding Y. Ren X. (2023). Effect of multi-glycosides of tripterygium wilfordii on renal injury in diabetic kidney disease rats through NLRP3/caspase-1/GSDMD pyroptosis pathway. China J. Chin. Mater. Med. 48 (10), 2639–2645. 10.19540/j.cnki.cjcmm.20230113.401 37282925

[B50] Song D. Jia P. Song K. Wang Y. (2026). Study on the mechanism of multi-glycosides of Tripterygium wilfordii against renal interstitial fibrosis in rats with diabetic nephropathy. Chin. Arch. Tradit. Chin. Med., 1–9. Available online at: https://link.cnki.net/urlid/21.1546.R.20260203.1036.008 (Accessed July 12, 2026).

[B51] Wang S. S. (2014). Intervention of Tripterygium wilfordii Polyglucosides and Berberine on the Glomerular Basement Membranes Related Factors in Rats with Diabetic Nephropathy. Tianjin: Tianjin Medical University. Master thesis. 10.7666/d.Y3192353

[B52] Wang X. Y. (2014). Research of the Intervention of Tripterygium wilfordii Polyglucosides and Berberine on Glomerular Endothelial Cell Related Factors in Rats with Diabetic Nephropathy. Tianjin: Tianjin Medical University. Master thesis. 10.7666/d.Y3192351

[B53] Wang Q. Du Q. Lei J. (2008). Abnormal expression of CTGF and drug intervention in rats with diabetic nephropathy. J. Shanghai Jiaot. Univ. Med. Sci. (05), 536–539.

[B54] Wang J. Cheng X. Zhu X. Zhang H. Wang Y. (2016a). Effect of the union of Yiqi Bushen Fang and tripterygium wilfordii polyglycosidium on expression of VEGF and secretion of IL-6 and TNF-α in rats with diabetic nephropathy. Chin. J. Mod. Appl. Pharm. 33 (06), 711–716. 10.13748/j.cnki.issn1007-7693.2016.06.08

[B55] Wang J. Cheng X. Zhu X. Yang Y. Wang Y. (2016b). Effect of Yiqi Bushen prescription together with GTW on expression of Nephrin and Podocin in rats with diabetic nephropathy. Chin. Arch. Tradit. Chin. Med. 34 (02), 365–369. 10.13193/j.issn.1673-7717.2016.02.032

[B56] Wang Y. Liu L. Yang P. (2017). Study on protective effect of tripterygium wilfordii polyglucosides in rats with type 2 diabetic kidney disease. Chin. J. Diabetes 25 (10), 909–913. 10.3969/j.issn.1006-6187.2017.10.011

[B57] Watanabe-Shimoji K. Tanabe H. Ohsugi M. Kawakami E. Tanaka K. Kazama J. J. (2025). Diverse combination of factors associated with the development of diabetic kidney disease among data-driven diabetes subtypes: analysis of the J-DREAMS registry. Diabetologia 69, 883–899. 10.1007/s00125-025-06594-1 41247492 PMC12957631

[B58] Wu W. Yang J.-J. Yang H.-M. Huang M.-M. Fang Q.-J. Shi G. (2017). Multi-glycoside of Tripterygium wilfordii Hook. f. attenuates glomerulosclerosis in a rat model of diabetic nephropathy by exerting anti-microinflammatory effects without affecting hyperglycemia. Int. J. Mol. Med. 40 (3), 721–730. 10.3892/ijmm.2017.3068 28731135 PMC5548017

[B59] Xu B. Xia L. Hu C. (2018). Effect of tripterygium wilfordii polyglycosidium on the RAGE/NF-κB signaling pathway in rats with STZ-induced diabetic nephropathy. J. Chengdu Med. Coll. 13 (03), 262–265. Available online at: https://link.cnki.net/urlid/51.1705.R.20180403.1610.006 (Accessed July 12, 2026).

[B60] Yang W. (2016). The Effect and Mechanism of Intervention of Tripterygium wilfordii Polyglucosides on Nephridial Tissue Fibrosis in Diabetic Rats. Tianjin: Tianjin Medical University. Master thesis. 10.7666/d.D01142982

[B61] Yang W. Song D. Song C. Song K. Chen C. Duan F. (2023). Effects of tripterygium glycosides tablets on Nrf2/Keap1/ARE signaling pathway in diabetic nephropathy rats. J. Li-shizhen Tradit. Chin. Med. 34 (01), 17–20. 10.3969/j.issn.1008-0805.2023.01.05

[B62] Yao L. Liang X. Qiao Y. Chen B. Wang P. Liu Z. (2022). Mitochondrial dysfunction in diabetic tubulopathy. Metabolism 131, 155195. 10.1016/j.metabol.2022.155195 35358497

[B63] Yao Z. Wang P. Fu Q. Song Q. Xu H. Zhang P. (2024). Efficacy and safety of tripterygium glycosides combined with ACEI/ARB on diabetic nephropathy: a meta-analysis. Front. Pharmacol. 15, 1493590. 10.3389/fphar.2024.1493590 39898319 PMC11782225

[B64] Yu X. He Y. Yang Y. Zhang G. Feng X. (2021). Tripterygium glycosides tablets affect the levels of heat shock protein 90 and PGC-1α in rats with diabetic nephropathy through PI3K/Akt pathway. China J. Mod. Med. 31 (22), 10–16. 10.3969/j.issn.1005-8982.2021.22.003

[B65] Zhang Y. X. (2014). The Antioxidative Effect of Tripterygium wilfordii Polyglycoside on Diabetic Rats and its Protective Mechanism. Xinxiang: Xinxiang Medical University. Master thesis.

[B66] Zhang H. Sun W. Wan Y. Che X. He F. Pu H. (2010). Preventive effects of multi-glycoside of Tripterygium wilfordii on glomerular lesions in experimental diabetic nephropathy. China J. Chin. Mater. Med. 35 (11), 1460–1465. 10.4268/cjcmm20101122 20822021

[B67] Zhang Y. Chang B. Chen W. (2012). Triptergium wilfordii polyglucosides interfere the expression of TGF-β1, BMP-7 and Gremlin in renal tissue of diabetic nephropathy rats. Chin. J. Nephrol. Dial. Transpl. 21 (03), 237–243. 10.3969/j.issn.1006-298X.2012.03.007

[B68] Zhang J. Chen W. Zhang H. Yin X. Yang P. Liu L. (2013). Effects of different doses of tripterygium glycosides on the expression of MDA, SOD and MCP-1 in kidney of rats with early diabetic nephropathy. Chin. J. Nephrol. 29 (7), 534–535. 10.3760/cma.j.issn.1001-7097.2013.07.012

[B69] Zhang Y. Liu D. Sun Y. Mo Z. (2013). Protective effect of tripterygium glycosides on kidney of diabetic rats and its possible inflammatory mechanism. Guangdong Med. J. 34 (17), 2634–2636. 10.13820/j.cnki.gdyx.2013.17.002

[B70] Zhang W. Ren X. Zhao C. Yu W. (2014). The impact of triperygium wilfordii polyglucoside in diabetic nephropathy rats renal tissue on Smad2/3 and Collagen type IV. Chin. J. Integr. Tradit. West. Nephrol. 15 (06), 487–490.

[B71] Zhang J. Zhang Y. Yin X. Yang P. Zhang H. Guo Y. (2014). Effects of triptergium glycosides on the expression of MCP-1 and CTGF in rats with early diabetic nephropathy. Chin. J. Nephrol. Dial. Transpl. 23 (02), 146–151+160.

[B72] Zhang L. Li H. Gao Y. Li J. Chen H. (2016). Protective effect of tripterygium glycosides on renal injury in diabetic nephropathy rats and its possible mechanism. Chin. J. Gerontol. 36 (14), 3389–3391. 10.3969/j.issn.1005-9202.2016.14.017

[B73] Zhang M. Chen Y. Yang M.-j. Fan X.-R. Xie H. Zhang L. (2019). Celastrol attenuates renal injury in diabetic rats *via* MAPK/NF-κB pathway. Phytother. Res. 33 (4), 1191–1198. 10.1002/ptr.6314 30768745

[B74] Zhang J. Ding T. Tang D. X. Wang J. P. (2020). Effect of tripterygium wilfordii polyglycoside on renal cell autophagy in rats with diabetic nephropathy and its related mechanism. Acta Anat. Sin. 51 (3), 373–377. 10.16098/j.issn.0529-1356.2020.03.010

[B75] Zhang J. Li S.-L. Lin W. Pan R.-H. Dai Y. Xia Y.-F. (2022). Tripterygium glycoside tablet attenuates renal function impairment in diabetic nephropathy mice by regulating triglyceride metabolism. J. Pharm. Biomed. Anal. 221, 115028. 10.1016/j.jpba.2022.115028 36108463

[B76] Zhang C. Song C. Wang M. Liang S. Guo X. Zhang H. (2025). Effects of tripterygium wilfordii multiglycoside on renal injury in rats with diabetic nephropathy. China Pharm. 36 (07), 815–819.

[B77] Zhang H. Guo X. Ding Y. Wang M. Song K. Chen C. (2026). Effects of multi-glycosides of tripterygium wilfordii in improving kidney injury in rats with diabetic kidney *via* regulation of mitochondrial homeostasis and ferroptosis. Chin. Pharmacol. Bull. 42 (01), 72–78. 10.12360/CPB202502028

[B78] Zhao Q. (2013). Mechanism of multi-glycosides of Tripterygium wilfordii Hook. F. for Ameliorating Glomerular Inflammatory Injury Through Regulating p38MAPK Signaling Pathway in Diabetic Nephropathy Rat. Nanjing: Nanjing University of Chinese Medicine. Master thesis.

[B79] Zhao R. (2014). Research of the Intervention of Tripterygium wilfordii Polyglucosides and Berberine on Glomerular Podocyte Related Factors in Rats with Diabetic Nephropathy. Tianjin: Tianjin Medical University. Master thesis. 10.7666/d.Y3192277

[B80] Zhao R. P. (2015). Effect of Tripterygium Wilfordii on Expression of Mannose-Binding Lectin and Inflammatory Cytokine in Kidney of Diabetic Model Rats. Bengbu: Bengbu Medical University. Master thesis. 10.7666/d.D666366

[B81] Zhao Y. (2016). The protective influence of tripterygium glucosides on kidney tissue of diabetic rats and expression of related inflammatory cytokines. Clin. J. Chin. Med. 8 (19), 7–8.

[B82] Zhao Q. Zheng M. Li Y. Chang B. Chen L. (2012). Effects of triperygium wilfordii polyglucoside on kidney structure and nephrin expression in podocytes in rats with diabetic nephropathy. Int. J. Endocrinol. Metab. 32 (4), 227–230. 10.3760/cma.j.issn.1673-4157.2012.04.003

[B83] Zhao Y. Ouyang J. Wang J. Zhang W. Zhang W. Meng J. (2013). Effect of tripterygium glycosides combined irbesartan on nephrin and podocin expression in the podocyte of diabetic nephropathy rats. Chin. J. Diabetes 5 (7), 414–417. 10.3760/cma.j.issn.1674-5809.2013.07.007

[B84] Zhao C. Yu W. Ren X. Zhang W. Li H. (2014). Effects of tripterygium wilfordii polyglucoside on expression of Activin A and influence of transdifferentiation in renal tubulointerstitium of rats with diabetic nephropathy. Natl. Med. J. China 94 (18), 1427–1432. 10.3760/cma.j.issn.0376-2491.2014.18.019 25142998

[B85] Zhao Y. Liu J. Chen Q. Xu J. Wang T. (2015). Therapeutic effect and mechanism of tripterygium glycosides administered by gavage on diabetic nephropathy in rats. Shandong Med. J. 55 (47), 31–33. 10.3969/j.issn.1002-266X.2015.47.011

[B86] Zhao R. P. Chen W. Chang B. Fan R. Liu L. Pan Y. (2017). Triperygium wilfordii multiglucoside ameliorates kidney damage in diabetic rats by inhibiting the expressions of MBL and MASP2. Chin. J. Cell. Mol. Immunol. 33 (11), 1498–1503. 10.13423/j.cnki.cjcmi.008480 29268853

[B87] Zheng Y. Hao L. Pan M. Ding N. (2011). Effect of tripterygium wilfordii polyglucoside on the podocytes of diabetic nephropathy rats. Chin. J. Nephrol. 27 (4), 288–292. 10.3760/cma.j.issn.1001-7097.2011.04.014

