# Supplementary material for: Renal protective effects and mechanisms of tripterygium glycosides in animal models of diabetic nephropathy: a preclinical systematic review and meta-analysis
Source: Front Pharmacol. 2026 Aug 25;17:1863463. doi: 10.3389/fphar.2026.1863463 (PMC13550136; doi:10.3389/fphar.2026.1863463)
Supplement: Supplementary file 1 [file Supplementaryfile1.docx]

Supplementary Material

Table of Contents

[1.	Search strategies in databases	3](#_Toc232067368)

[1.1 CNKI (183)	3](#_Toc232067369)

[1.2 Wanfang (115)	4](#_Toc232067370)

[1.3 VIP (72)	5](#_Toc232067371)

[1.4 SinoMed (67)	6](#_Toc232067372)

[1.5 PubMed (11)	7](#_Toc232067373)

[1.6 EMBASE (22)	9](#_Toc232067374)

[1.7 Web of Science (36)	10](#_Toc232067375)

[1.8 Cochrane Library (0)	12](#_Toc232067376)

[2.	Supporting information for forest plot	14](#_Toc232067377)

[2.1 Forest plot of the effect of TGs on SOD(A), serum CAT(B), GPx(C) and MDA(D)	14](#_Toc232067378)

[2.2 Forest plot of the effect of TGs on TNF-α(A) and TNF-α mRNA(B)	14](#_Toc232067379)

[2.3 Forest plot of the effect of TGs on IL-1β(A) and IL-1β mRNA(B)	15](#_Toc232067380)

[2.4 Forest plot of the effect of TGs on MCP-1(A) and MCP-1 mRNA(B)	15](#_Toc232067381)

[2.5 Forest plot of the effect of TGs on VCAM-1(A) and VCAM-1 mRNA(B)	15](#_Toc232067382)

[2.6 Forest plot of the effect of TGs on NF-κB(A) and NF-κB mRNA(B)	16](#_Toc232067383)

[2.7 Forest plot of the effect of TGs on p-p38MAPK/p38MAPK	16](#_Toc232067384)

[2.8 Forest plot of the effect of TGs on p-JAK2(A) and JAK2 mRNA(B)	16](#_Toc232067385)

[2.9 Forest plot of the effect of TGs on p-STAT3(A) and STAT3 mRNA(B)	17](#_Toc232067386)

[2.10 Forest plot of the effect of TGs on TGF-β1(A) and TGF-β1 mRNA(B)	17](#_Toc232067387)

[2.11 Forest plot of the effect of TGs on CTGF(A) and CTGF mRNA(B)	17](#_Toc232067388)

[2.12 Forest plot of the effect of TGs on α-SMA(A) and α-SMA mRNA(B)	18](#_Toc232067389)

[2.13 Forest plot of the effect of TGs on Col-IV(A) and Col-IV mRNA(B)	18](#_Toc232067390)

[2.14 Forest plot of the effect of TGs on LN(A) and LN mRNA(B)	18](#_Toc232067391)

[2.15 Forest plot of the effect of TGs on Wnt1(A) and Wnt1 mRNA(B)	19](#_Toc232067392)

[2.16 Forest plot of the effect of TGs on β-catenin(A) and β-catenin mRNA(B)	19](#_Toc232067393)

[2.17 Forest plot of the effect of TGs on Nephrin(A) and Nephrin mRNA(B)	19](#_Toc232067394)

[2.18 Forest plot of the effect of TGs on Podocin(A) and Podocin mRNA(B)	20](#_Toc232067395)

[2.19 Forest plot of the effect of TGs on VEGF(A) and VEGF mRNA(B)	20](#_Toc232067396)

[2.20 Forest plot of the effect of TGs on p-PI3K/PI3K(A), p-PI3K(B) and PI3K(C)	20](#_Toc232067397)

[2.21 Forest plot of the effect of TGs on p-Akt/Akt(A), p-Akt(B), p-Akt mRNA(C), Akt(D), and Akt mRNA(E)	21](#_Toc232067398)

[2.22 Forest plot of the effect of TGs on p-mTOR/mTOR(A), p-mTOR(B), p-mTOR mRNA(C), mTOR(D), and mTOR mRNA(E)	22](#_Toc232067399)

[2.23 Forest plot of the effect of TGs on LC3-II/LC3-I(A) and LC3 mRNA(B)	22](#_Toc232067400)

[2.24 Forest plot of the effect of TGs on Beclin-1(A) and Beclin-1 mRNA(B)	23](#_Toc232067401)

[3.	Supporting information for sensitivity analysis	23](#_Toc232067402)

[3.1 Sensitivity analysis of the effect of TGs on Scr	23](#_Toc232067403)

[3.2 Sensitivity analysis of the effect of TGs on BUN	24](#_Toc232067404)

[3.3 Sensitivity analysis of the effect of TGs on 24h UP	24](#_Toc232067405)

[3.4 Sensitivity analysis of the effect of TGs on 24h UA	25](#_Toc232067406)

[3.5 Sensitivity analysis of the effect of TGs on 24h UMA	25](#_Toc232067407)

[3.6 Sensitivity analysis of the effect of TGs on KW	26](#_Toc232067408)

[3.7 Sensitivity analysis of the effect of TGs on KI	26](#_Toc232067409)

[3.8 Sensitivity analysis of the effect of TGs on BG	27](#_Toc232067410)

[3.9 Sensitivity analysis of the effect of TGs on TC	27](#_Toc232067411)

[3.10 Sensitivity analysis of the effect of TGs on TG	28](#_Toc232067412)

[3.11 Sensitivity analysis of the effect of TGs on ALT	28](#_Toc232067413)

[3.12 Sensitivity analysis of the effect of TGs on MGV	29](#_Toc232067414)

[4.	Supporting information for subgroup analysis	30](#_Toc232067415)

[4.1 Subgroup analysis of the effect of TGs on Scr based on species	30](#_Toc232067416)

[4.2 Subgroup analysis of the effect of TGs on BG based on species	31](#_Toc232067417)

[4.3 Subgroup analysis of the effect of TGs on Scr based on sex	32](#_Toc232067418)

[4.4 Subgroup analysis of the effect of TGs on BUN based on sex	33](#_Toc232067419)

[4.5 Subgroup analysis of the effect of TGs on KW based on sex	34](#_Toc232067420)

[4.6 Subgroup analysis of the effect of TGs on KI based on sex	34](#_Toc232067421)

[4.7 Subgroup analysis of the effect of TGs on BG based on sex	35](#_Toc232067422)

[4.8 Subgroup analysis of the effect of TGs on ALT based on sex	36](#_Toc232067423)

[4.9 Subgroup analysis of the effect of TGs on Scr based on type of DM	37](#_Toc232067424)

[4.10 Subgroup analysis of the effect of TGs on BUN based on type of DM	38](#_Toc232067425)

[4.11 Subgroup analysis of the effect of TGs on 24h UP based on type of DM	39](#_Toc232067426)

[4.12 Subgroup analysis of the effect of TGs on 24h UMA based on type of DM	39](#_Toc232067427)

[4.13 Subgroup analysis of the effect of TGs on KW based on type of DM	40](#_Toc232067428)

[4.14 Subgroup analysis of the effect of TGs on KI based on type of DM	40](#_Toc232067429)

[4.15 Subgroup analysis of the effect of TGs on BG based on type of DM	41](#_Toc232067430)

[4.16 Subgroup analysis of the effect of TGs on TC based on type of DM	42](#_Toc232067431)

[4.17 Subgroup analysis of the effect of TGs on TG based on type of DM	42](#_Toc232067432)

[4.18 Subgroup analysis of the effect of TGs on ALT based on type of DM	43](#_Toc232067433)

[4.19 Subgroup analysis of the effect of TGs on MGV based on type of DM	43](#_Toc232067434)

[4.20 Subgroup analysis of the effect of TGs on Scr based on number of study arms	44](#_Toc232067435)

[4.21 Subgroup analysis of the effect of TGs on BUN based on number of study arms	45](#_Toc232067436)

[4.22 Subgroup analysis of the effect of TGs on 24h UP based on number of study arms	46](#_Toc232067437)

[4.23 Subgroup analysis of the effect of TGs on 24h UA based on number of study arms	46](#_Toc232067438)

[4.24 Subgroup analysis of the effect of TGs on 24h UMA based on number of study arms	47](#_Toc232067439)

[4.25 Subgroup analysis of the effect of TGs on KW based on number of study arms	47](#_Toc232067440)

[4.26 Subgroup analysis of the effect of TGs on KI based on number of study arms	48](#_Toc232067441)

[4.27 Subgroup analysis of the effect of TGs on BG based on number of study arms	49](#_Toc232067442)

[4.28 Subgroup analysis of the effect of TGs on TC based on number of study arms	50](#_Toc232067443)

[4.29 Subgroup analysis of the effect of TGs on TG based on number of study arms	50](#_Toc232067444)

[4.30 Subgroup analysis of the effect of TGs on ALT based on number of study arms	51](#_Toc232067445)

[4.31 Subgroup analysis of the effect of TGs on MGV based on number of study arms	51](#_Toc232067446)

[4.32 Subgroup analysis of the effect of TGs on Scr based on intervention duration	52](#_Toc232067447)

[4.33 Subgroup analysis of the effect of TGs on BUN based on intervention duration	53](#_Toc232067448)

[4.34 Subgroup analysis of the effect of TGs on 24h UP based on intervention duration	54](#_Toc232067449)

[4.35 Subgroup analysis of the effect of TGs on KI based on intervention duration	55](#_Toc232067450)

[4.36 Subgroup analysis of the effect of TGs on BG based on intervention duration	56](#_Toc232067451)

[4.37 Subgroup analysis of the effect of TGs on ALT based on intervention duration	57](#_Toc232067452)

[5.	Supporting information for meta-regression analysis	57](#_Toc232067453)

[5.1 Table of results from meta-regression analysis	57](#_Toc232067454)

[6.	Supporting information for publication bias	60](#_Toc232067455)

[6.1 Funnel plot of the effect of TGs on Scr(A), BUN(B), 24h UP(C), KW(D), KI(E), BG(F), TC(G), TG(H), ALT(I), AST(J) and ALB(K)	60](#_Toc232067456)

[6.2 Egger's test plot of the effect of TGs on Scr(A), BUN(B), 24h UP(C), KW(D), KI(E), BG(F), TC(G), TG(H), ALT(I), AST(J) and ALB(K)	61](#_Toc232067457)

[6.3 Filled funnel plot of the effect of TGs on Scr(A), BUN(B), 24h UP(C), KI(D), BG(E), ALT(F)	62](#_Toc232067458)

[6.4 Table of trim-and-fill analysis results for Scr(A), BUN(B), 24h UP(C), KI(D), BG(E), ALT(F)	62](#_Toc232067459)

[7.	Supporting information for evidence certainty	63](#_Toc232067460)

[7.1 Evidence quality grade table of important outcomes	63](#_Toc232067461)

1. Search strategies in databases

1.1 CNKI (183)

The database search in CNKI was carried out on May 19, 2026, and a total of 183 studies were found. The search query is as follows:

(SU %= '雷公藤多苷' OR SU %= '雷公藤多甙' OR SU %= '雷公藤多苷片' OR SU %= '雷公藤多甙片' OR SU %= '雷公藤多苷提取物' OR SU %= '雷公藤多甙提取物' OR TKA = '雷公藤多苷' OR TKA = '雷公藤多甙' OR TKA = '雷公藤多苷片' OR TKA = '雷公藤多甙片' OR TKA = '雷公藤多苷提取物' OR TKA = '雷公藤多甙提取物') AND (SU %= '糖尿病肾病' OR SU %= '糖尿病肾脏病' OR SU %= '糖尿病肾脏疾病' OR SU %= '糖尿病肾小球硬化症' OR SU %= '糖尿病性肾病' OR SU %= '糖尿病性肾脏病' OR SU %= '糖尿病性肾脏疾病' OR SU %= '糖尿病性肾小球硬化症' OR TKA = '糖尿病肾病' OR TKA = '糖尿病肾脏病' OR TKA = '糖尿病肾脏疾病' OR TKA = '糖尿病肾小球硬化症' OR TKA = '糖尿病性肾病' OR TKA = '糖尿病性肾脏病' OR TKA = '糖尿病性肾脏疾病' OR TKA = '糖尿病性肾小球硬化症') AND (SU %= '动物' OR SU %= '动物模型' OR SU %= '动物实验' OR SU %= '实验动物' OR SU %= '大鼠' OR SU %= '大鼠模型' OR SU %= '小鼠' OR SU %= '小鼠模型' OR TKA = '动物' OR TKA = '动物模型' OR TKA = '动物实验' OR TKA = '实验动物' OR TKA = '大鼠' OR TKA = '大鼠模型' OR TKA = '小鼠' OR TKA = '小鼠模型')

1.2 Wanfang (115)

The database search in Wanfang was carried out on May 19, 2026, and a total of 115 studies were found. The search query is as follows:

(主题:(雷公藤多苷 or 雷公藤多甙 or 雷公藤多苷片 or 雷公藤多甙片 or 雷公藤多苷提取物 or 雷公藤多甙提取物) or 题名或关键词:(雷公藤多苷 or 雷公藤多甙 or 雷公藤多苷片 or 雷公藤多甙片 or 雷公藤多苷提取物 or 雷公藤多甙提取物) or 摘要:(雷公藤多苷 or 雷公藤多甙 or 雷公藤多苷片 or 雷公藤多甙片 or 雷公藤多苷提取物 or 雷公藤多甙提取物)) and (主题:(糖尿病肾病 or 糖尿病肾脏病 or 糖尿病肾脏疾病 or 糖尿病肾小球硬化症 or 糖尿病性肾病 or 糖尿病性肾脏病 or 糖尿病性肾脏疾病 or 糖尿病性肾小球硬化症) or 题名或关键词:(糖尿病肾病 or 糖尿病肾脏病 or 糖尿病肾脏疾病 or 糖尿病肾小球硬化症 or 糖尿病性肾病 or 糖尿病性肾脏病 or 糖尿病性肾脏疾病 or 糖尿病性肾小球硬化症) or 摘要:(糖尿病肾病 or 糖尿病肾脏病 or 糖尿病肾脏疾病 or 糖尿病肾小球硬化症 or 糖尿病性肾病 or 糖尿病性肾脏病 or 糖尿病性肾脏疾病 or 糖尿病性肾小球硬化症)) and (主题:(动物 or 动物模型 or 动物实验 or 实验动物 or 大鼠 or 大鼠模型 or 小鼠 or 小鼠模型) or 题名或关键词:(动物 or 动物模型 or 动物实验 or 实验动物 or 大鼠 or 大鼠模型 or 小鼠 or 小鼠模型) or 摘要:(动物 or 动物模型 or 动物实验 or 实验动物 or 大鼠 or 大鼠模型 or 小鼠 or 小鼠模型))

1.3 VIP (72)

The database search in VIP was carried out on May 19, 2026, and a total of 72 studies were found. The search query is as follows:

(M=(雷公藤多苷 OR 雷公藤多甙 OR 雷公藤多苷片 OR 雷公藤多甙片 OR 雷公藤多苷提取物 OR 雷公藤多甙提取物) OR R=(雷公藤多苷 OR 雷公藤多甙 OR 雷公藤多苷片 OR 雷公藤多甙片 OR 雷公藤多苷提取物 OR 雷公藤多甙提取物)) AND (M=(糖尿病肾病 OR 糖尿病肾脏病 OR 糖尿病肾脏疾病 OR 糖尿病肾小球硬化症 OR 糖尿病性肾病 OR 糖尿病性肾脏病 OR 糖尿病性肾脏疾病 OR 糖尿病性肾小球硬化症) OR R=(糖尿病肾病 OR 糖尿病肾脏病 OR 糖尿病肾脏疾病 OR 糖尿病肾小球硬化症 OR 糖尿病性肾病 OR 糖尿病性肾脏病 OR 糖尿病性肾脏疾病 OR 糖尿病性肾小球硬化症)) AND (M=(动物 OR 动物模型 OR 动物实验 OR 实验动物 OR 大鼠 OR 大鼠模型 OR 小鼠 OR 小鼠模型) OR R=(动物 OR 动物模型 OR 动物实验 OR 实验动物 OR 大鼠 OR 大鼠模型 OR 小鼠 OR 小鼠模型))

1.4 SinoMed (67)

The database search in SinoMed was carried out on May 19, 2026, and a total of 67 studies were found. The search query is as follows:

( "雷公藤多苷"[常用字段:智能] OR "雷公藤多甙"[常用字段:智能] OR "雷公藤多苷片"[常用字段:智能] OR "雷公藤多甙片"[常用字段:智能] OR "雷公藤多苷提取物"[常用字段:智能] OR "雷公藤多甙提取物"[常用字段:智能]) AND( "糖尿病肾病"[常用字段:智能] OR "糖尿病肾脏病"[常用字段:智能] OR "糖尿病肾脏疾病"[常用字段:智能] OR "糖尿病肾小球硬化症"[常用字段:智能] OR "糖尿病性肾病"[常用字段:智能] OR "糖尿病性肾脏病"[常用字段:智能] OR "糖尿病性肾脏疾病"[常用字段:智能] OR "糖尿病性肾小球硬化症"[常用字段:智能]) AND( "动物"[常用字段:智能] OR "动物模型"[常用字段:智能] OR "动物实验"[常用字段:智能] OR "实验动物"[常用字段:智能] OR "大鼠"[常用字段:智能] OR "大鼠模型"[常用字段:智能] OR "小鼠"[常用字段:智能] OR "小鼠模型"[常用字段:智能])

1.5 PubMed (11)

The database search in PubMed was carried out on May 19, 2026, and a total of 11 studies were found. The search query is as follows:

| Search number | Query | Results |
| --- | --- | --- |
| 11 | #1 AND #4 AND #10 | 11 |
| 10 | (#5 OR #6) OR (#7 OR #8) OR #9 | 4,151,056 |
| 9 | ((((animal*[Title/Abstract]) OR (rat[Title/Abstract])) OR (rats[Title/Abstract])) OR (mouse[Title/Abstract])) OR (mice[Title/Abstract]) | 3,984,314 |
| 8 | ((((((((((((((((Animal Experimentation[Title/Abstract]) OR (Experimentation, Animal[Title/Abstract])) OR (Animal Experimental Use[Title/Abstract])) OR (Animal Experimental Uses[Title/Abstract])) OR (Experimental Use, Animal[Title/Abstract])) OR (Experimental Uses, Animal[Title/Abstract])) OR (Animal Research[Title/Abstract])) OR (Research, Animal[Title/Abstract])) OR (Animal Experiments[Title/Abstract])) OR (Animal Experiment[Title/Abstract])) OR (Experiment, Animal[Title/Abstract])) OR (Experiments, Animal[Title/Abstract])) OR (animal physical conditioning[Title/Abstract])) OR (animal studies[Title/Abstract])) OR (animal study[Title/Abstract])) OR (animal trial[Title/Abstract])) OR (physical conditioning, animal[Title/Abstract]) | 83,609 |
| 7 | "Animal Experimentation"[Mesh] | 10,884 |
| 6 | ((((((((((((((((Models, Animal[Title/Abstract]) OR (Animal Model[Title/Abstract])) OR (Animal Models[Title/Abstract])) OR (Model, Animal[Title/Abstract])) OR (Experimental Animal Models[Title/Abstract])) OR (Animal Model, Experimental[Title/Abstract])) OR (Animal Models, Experimental[Title/Abstract])) OR (Experimental Animal Model[Title/Abstract])) OR (Model, Experimental Animal[Title/Abstract])) OR (Models, Experimental Animal[Title/Abstract])) OR (Laboratory Animal Models[Title/Abstract])) OR (Animal Model, Laboratory[Title/Abstract])) OR (Animal Models, Laboratory[Title/Abstract])) OR (Laboratory Animal Model[Title/Abstract])) OR (Model, Laboratory Animal[Title/Abstract])) OR (Models, Laboratory Animal[Title/Abstract])) OR (animal disease model[Title/Abstract]) | 248,674 |
| 5 | "Models, Animal"[Mesh] | 714,221 |
| 4 | #2 OR #3 | 47,136 |
| 3 | (((((((((((((((((((Diabetic Nephropathies[Title/Abstract]) OR (Nephropathies, Diabetic[Title/Abstract])) OR (Nephropathy, Diabetic[Title/Abstract])) OR (Diabetic Kidney Disease[Title/Abstract])) OR (Diabetic Kidney Diseases[Title/Abstract])) OR (Kidney Disease, Diabetic[Title/Abstract])) OR (Kidney Diseases, Diabetic[Title/Abstract])) OR (Diabetic Nephropathy[Title/Abstract])) OR (Diabetic Glomerulosclerosis[Title/Abstract])) OR (Glomerulosclerosis, Diabetic[Title/Abstract])) OR (Intracapillary Glomerulosclerosis[Title/Abstract])) OR (Kimmelstiel-Wilson Disease[Title/Abstract])) OR (Kimmelstiel Wilson Disease[Title/Abstract])) OR (Nodular Glomerulosclerosis[Title/Abstract])) OR (Glomerulosclerosis, Nodular[Title/Abstract])) OR (Kimmelstiel-Wilson Syndrome[Title/Abstract])) OR (Kimmelstiel Wilson Syndrome[Title/Abstract])) OR (Syndrome, Kimmelstiel-Wilson[Title/Abstract])) OR (diabetes nephropathy[Title/Abstract])) OR (diabetic renal disease[Title/Abstract]) | 34,421 |
| 2 | "Diabetic Nephropathies"[Mesh] | 33,574 |
| 1 | ((((((((((tripterygium glycoside*[Title/Abstract]) OR (tripterygium wilfordii glycoside*[Title/Abstract])) OR (tripterygium wilfordii polyglycoside*[Title/Abstract])) OR (tripterygium wilfordii poly-glycoside*[Title/Abstract])) OR (polyglycoside* of tripterygium wilfordii[Title/Abstract])) OR (poly-glycoside* of tripterygium wilfordii[Title/Abstract])) OR (tripterygium wilfordii multiglycoside*[Title/Abstract])) OR (tripterygium wilfordii multi-glycoside*[Title/Abstract])) OR (multiglycoside* of tripterygium wilfordii[Title/Abstract])) OR (multi-glycoside* of tripterygium wilfordii[Title/Abstract])) OR (leigongteng duogan[Title/Abstract]) | 470 |

1.6 EMBASE (22)

The database search in EMBASE was carried out on May 19, 2026, and a total of 22 studies were found. The search query is as follows:

| No. | Query | Results |
| --- | --- | --- |
| #11 | #1 AND #4 AND #10 | 22 |
| #10 | #5 OR #6 OR #7 OR #8 OR #9 | 6,186,464 |
| #9 | 'animal*':ti,ab,kw OR 'rat':ti,ab,kw OR 'rats':ti,ab,kw OR 'mouse':ti,ab,kw OR 'mice':ti,ab,kw | 5,005,569 |
| #8 | 'animal experiment':ti,ab,kw OR 'animal experimentation':ti,ab,kw OR 'experimentation, animal':ti,ab,kw OR 'animal experimental use':ti,ab,kw OR 'animal experimental uses':ti,ab,kw OR 'experimental use, animal':ti,ab,kw OR 'experimental uses, animal':ti,ab,kw OR 'animal research':ti,ab,kw OR 'research, animal':ti,ab,kw OR 'animal experiments':ti,ab,kw OR 'experiment, animal':ti,ab,kw OR 'experiments, animal':ti,ab,kw OR 'animal physical conditioning':ti,ab,kw OR 'animal studies':ti,ab,kw OR 'animal study':ti,ab,kw OR 'animal trial':ti,ab,kw OR 'physical conditioning, animal':ti,ab,kw | 115,002 |
| #7 | 'animal experiment'/exp | 3,566,279 |
| #6 | 'animal model':ti,ab,kw OR 'models, animal':ti,ab,kw OR 'animal models':ti,ab,kw OR 'model, animal':ti,ab,kw OR 'experimental animal models':ti,ab,kw OR 'animal model, experimental':ti,ab,kw OR 'animal models, experimental':ti,ab,kw OR 'experimental animal model':ti,ab,kw OR 'model, experimental animal':ti,ab,kw OR 'models, experimental animal':ti,ab,kw OR 'laboratory animal models':ti,ab,kw OR 'animal model, laboratory':ti,ab,kw OR 'animal models, laboratory':ti,ab,kw OR 'laboratory animal model':ti,ab,kw OR 'model, laboratory animal':ti,ab,kw OR 'models, laboratory animal':ti,ab,kw OR 'animal disease model':ti,ab,kw | 344,715 |
| #5 | 'animal model'/exp | 2,128,260 |
| #4 | #2 OR #3 | 77,163 |
| #3 | 'diabetic nephropathy':ti,ab,kw OR 'diabetic nephropathies':ti,ab,kw OR 'nephropathies, diabetic':ti,ab,kw OR 'nephropathy, diabetic':ti,ab,kw OR 'diabetic kidney disease':ti,ab,kw OR 'diabetic kidney diseases':ti,ab,kw OR 'kidney disease, diabetic':ti,ab,kw OR 'kidney diseases, diabetic':ti,ab,kw OR 'diabetic glomerulosclerosis':ti,ab,kw OR 'glomerulosclerosis, diabetic':ti,ab,kw OR 'intracapillary glomerulosclerosis':ti,ab,kw OR 'kimmelstiel-wilson disease':ti,ab,kw OR 'kimmelstiel wilson disease':ti,ab,kw OR 'nodular glomerulosclerosis':ti,ab,kw OR 'glomerulosclerosis, nodular':ti,ab,kw OR 'kimmelstiel-wilson syndrome':ti,ab,kw OR 'kimmelstiel wilson syndrome':ti,ab,kw OR 'syndrome, kimmelstiel-wilson':ti,ab,kw OR 'diabetes nephropathy':ti,ab,kw OR 'diabetic renal disease':ti,ab,kw | 51,870 |
| #2 | 'diabetic nephropathy'/exp | 68,571 |
| #1 | 'tripterygium glycoside*':ti,ab,kw OR 'tripterygium wilfordii glycoside*':ti,ab,kw OR 'tripterygium wilfordii polyglycoside*':ti,ab,kw OR 'tripterygium wilfordii poly-glycoside*':ti,ab,kw OR 'polyglycoside* of tripterygium wilfordii':ti,ab,kw OR 'poly-glycoside* of tripterygium wilfordii':ti,ab,kw OR 'tripterygium wilfordii multiglycoside*':ti,ab,kw OR 'tripterygium wilfordii multi-glycoside*':ti,ab,kw OR 'multiglycoside* of tripterygium wilfordii':ti,ab,kw OR 'multi-glycoside* of tripterygium wilfordii':ti,ab,kw OR 'leigongteng duogan':ti,ab,kw | 658 |

1.7 Web of Science (36)

The database search in Web of Science was carried out on May 19, 2026, and a total of 36 studies were found. The search query is as follows:

| # | Search Query | Results |
| --- | --- | --- |
| 1 | TS=("tripterygium glycoside*" OR "tripterygium wilfordii glycoside*" OR "tripterygium wilfordii polyglycoside*" OR "tripterygium wilfordii poly-glycoside*" OR "polyglycoside* of tripterygium wilfordii" OR "poly-glycoside* of tripterygium wilfordii" OR "tripterygium wilfordii multiglycoside*" OR "tripterygium wilfordii multi-glycoside*" OR "multiglycoside* of tripterygium wilfordii" OR "multi-glycoside* of tripterygium wilfordii" OR "leigongteng duogan") and Preprint Citation Index (Exclude – Database) and Research Commons (Exclude – Database) | 566 |
| 2 | TS=("Diabetic Nephropathies" OR "Nephropathies, Diabetic" OR "Nephropathy, Diabetic" OR "Diabetic Kidney Disease" OR "Diabetic Kidney Diseases" OR "Kidney Disease, Diabetic" OR "Kidney Diseases, Diabetic" OR "Diabetic Nephropathy" OR "Diabetic Glomerulosclerosis" OR "Glomerulosclerosis, Diabetic" OR "Intracapillary Glomerulosclerosis" OR "Kimmelstiel-Wilson Disease" OR "Kimmelstiel Wilson Disease" OR "Nodular Glomerulosclerosis" OR "Glomerulosclerosis, Nodular" OR "Kimmelstiel-Wilson Syndrome" OR "Kimmelstiel Wilson Syndrome" OR "Syndrome, Kimmelstiel-Wilson" OR "diabetes nephropathy" OR "diabetic renal disease") and Preprint Citation Index (Exclude – Database) and Research Commons (Exclude – Database) | 64,457 |
| 3 | TS=("Models, Animal" OR "Animal Model" OR "Animal Models" OR "Model, Animal" OR "Experimental Animal Models" OR "Animal Model, Experimental" OR "Animal Models, Experimental" OR "Experimental Animal Model" OR "Model, Experimental Animal" OR "Models, Experimental Animal" OR "Laboratory Animal Models" OR "Animal Model, Laboratory" OR "Animal Models, Laboratory" OR "Laboratory Animal Model" OR "Model, Laboratory Animal" OR "Models, Laboratory Animal" OR "animal disease model") and Preprint Citation Index (Exclude – Database) and Research Commons (Exclude – Database) | 1,148,699 |
| 4 | TS=("Animal Experimentation" OR "Experimentation, Animal" OR "Animal Experimental Use" OR "Animal Experimental Uses" OR "Experimental Use, Animal" OR "Experimental Uses, Animal" OR "Animal Research" OR "Research, Animal" OR "Animal Experiments" OR "Animal Experiment" OR "Experiment, Animal" OR "Experiments, Animal" OR "animal physical conditioning" OR "animal studies" OR "animal study" OR "animal trial" OR "physical conditioning, animal") and Preprint Citation Index (Exclude – Database) and Research Commons (Exclude – Database) | 123,363 |
| 5 | TS=("animal*" OR "rat" OR "rats" OR "mouse" OR "mice") and Preprint Citation Index (Exclude – Database) and Research Commons (Exclude – Database) | 21,595,699 |
| 6 | #3 OR #4 OR #5 and Preprint Citation Index (Exclude – Database) and Research Commons (Exclude – Database) | 21,595,699 |
| 7 | #1 AND #2 AND #6 and Preprint Citation Index (Exclude – Database) and Research Commons (Exclude – Database) | 36 |

1.8 Cochrane Library (0)

The database search in Cochrane Library was carried out on May 19, 2026, and no studies were were found. The search query is as follows:

| ID | Search | Hits |
| --- | --- | --- |
| #1 | ((tripterygium glycoside*) OR (tripterygium wilfordii glycoside*) OR (tripterygium wilfordii polyglycoside*) OR (tripterygium wilfordii poly-glycoside*) OR (polyglycoside* of tripterygium wilfordii) OR (poly-glycoside* of tripterygium wilfordii) OR (tripterygium wilfordii multiglycoside*) OR (tripterygium wilfordii multi-glycoside*) OR (multiglycoside* of tripterygium wilfordii) OR (multi-glycoside* of tripterygium wilfordii) OR (leigongteng duogan)):ti,ab,kw (Word variations have been searched) | 95 |
| #2 | MeSH descriptor: [Diabetic Nephropathies] explode all trees | 2094 |
| #3 | ((Diabetic Nephropathies) OR (Nephropathies, Diabetic) OR (Nephropathy, Diabetic) OR (Diabetic Kidney Disease) OR (Diabetic Kidney Diseases) OR (Kidney Disease, Diabetic) OR (Kidney Diseases, Diabetic) OR (Diabetic Nephropathy) OR (Diabetic Glomerulosclerosis) OR (Glomerulosclerosis, Diabetic) OR (Intracapillary Glomerulosclerosis) OR (Kimmelstiel-Wilson Disease) OR (Kimmelstiel Wilson Disease) OR (Nodular Glomerulosclerosis) OR (Glomerulosclerosis, Nodular) OR (Kimmelstiel-Wilson Syndrome) OR (Kimmelstiel Wilson Syndrome) OR (Syndrome, Kimmelstiel-Wilson) OR (diabetes nephropathy) OR (diabetic renal disease)):ti,ab,kw (Word variations have been searched) | 16612 |
| #4 | #2 OR #3 | 16612 |
| #5 | MeSH descriptor: [Models, Animal] explode all trees | 1110 |
| #6 | ((Models, Animal) OR (Animal Model) OR (Animal Models) OR (Model, Animal) OR (Experimental Animal Models) OR (Animal Model, Experimental) OR (Animal Models, Experimental) OR (Experimental Animal Model) OR (Model, Experimental Animal) OR (Models, Experimental Animal) OR (Laboratory Animal Models) OR (Animal Model, Laboratory) OR (Animal Models, Laboratory) OR (Laboratory Animal Model) OR (Model, Laboratory Animal) OR (Models, Laboratory Animal) OR (animal disease model)):ti,ab,kw (Word variations have been searched) | 14256 |
| #7 | MeSH descriptor: [Animal Experimentation] explode all trees | 10 |
| #8 | ((Animal Experimentation) OR (Experimentation, Animal) OR (Animal Experimental Use) OR (Animal Experimental Uses) OR (Experimental Use, Animal) OR (Experimental Uses, Animal) OR (Animal Research) OR (Research, Animal) OR (Animal Experiments) OR (Animal Experiment) OR (Experiment, Animal) OR (Experiments, Animal) OR (animal physical conditioning) OR (animal studies) OR (animal study) OR (animal trial) OR (physical conditioning, animal)):ti,ab,kw (Word variations have been searched) | 38747 |
| #9 | ((animal*) OR (rat) OR (rats) OR (mouse) OR (mice)):ti,ab,kw (Word variations have been searched) | 45377 |
| #10 | (#5 OR #6) OR (#7 OR #8) OR #9 | 46828 |
| #11 | #1 AND #4 AND #10 | 0 |

2. Supporting information for forest plot

2.1 Forest plot of the effect of TGs on SOD(A), serum CAT(B), GPx(C) and MDA(D)

2.2 Forest plot of the effect of TGs on TNF-α(A) and TNF-α mRNA(B)

2.3 Forest plot of the effect of TGs on IL-1β(A) and IL-1β mRNA(B)

2.4 Forest plot of the effect of TGs on MCP-1(A) and MCP-1 mRNA(B)

2.5 Forest plot of the effect of TGs on VCAM-1(A) and VCAM-1 mRNA(B)

2.6 Forest plot of the effect of TGs on NF-κB(A) and NF-κB mRNA(B)

2.7 Forest plot of the effect of TGs on p-p38MAPK/p38MAPK

2.8 Forest plot of the effect of TGs on p-JAK2(A) and JAK2 mRNA(B)

2.9 Forest plot of the effect of TGs on p-STAT3(A) and STAT3 mRNA(B)

2.10 Forest plot of the effect of TGs on TGF-β1(A) and TGF-β1 mRNA(B)

2.11 Forest plot of the effect of TGs on CTGF(A) and CTGF mRNA(B)

2.12 Forest plot of the effect of TGs on α-SMA(A) and α-SMA mRNA(B)

2.13 Forest plot of the effect of TGs on Col-IV(A) and Col-IV mRNA(B)

2.14 Forest plot of the effect of TGs on LN(A) and LN mRNA(B)

2.15 Forest plot of the effect of TGs on Wnt1(A) and Wnt1 mRNA(B)

2.16 Forest plot of the effect of TGs on β-catenin(A) and β-catenin mRNA(B)

2.17 Forest plot of the effect of TGs on Nephrin(A) and Nephrin mRNA(B)

2.18 Forest plot of the effect of TGs on Podocin(A) and Podocin mRNA(B)

2.19 Forest plot of the effect of TGs on VEGF(A) and VEGF mRNA(B)

2.20 Forest plot of the effect of TGs on p-PI3K/PI3K(A), p-PI3K(B) and PI3K(C)

2.21 Forest plot of the effect of TGs on p-Akt/Akt(A), p-Akt(B), p-Akt mRNA(C), Akt(D), and Akt mRNA(E)

2.22 Forest plot of the effect of TGs on p-mTOR/mTOR(A), p-mTOR(B), p-mTOR mRNA(C), mTOR(D), and mTOR mRNA(E)

2.23 Forest plot of the effect of TGs on LC3-II/LC3-I(A) and LC3 mRNA(B)

2.24 Forest plot of the effect of TGs on Beclin-1(A) and Beclin-1 mRNA(B)

3. Supporting information for sensitivity analysis

3.1 Sensitivity analysis of the effect of TGs on Scr

3.2 Sensitivity analysis of the effect of TGs on BUN

3.3 Sensitivity analysis of the effect of TGs on 24h UP

3.4 Sensitivity analysis of the effect of TGs on 24h UA

3.5 Sensitivity analysis of the effect of TGs on 24h UMA

3.6 Sensitivity analysis of the effect of TGs on KW

3.7 Sensitivity analysis of the effect of TGs on KI

3.8 Sensitivity analysis of the effect of TGs on BG

3.9 Sensitivity analysis of the effect of TGs on TC

3.10 Sensitivity analysis of the effect of TGs on TG

3.11 Sensitivity analysis of the effect of TGs on ALT

3.12 Sensitivity analysis of the effect of TGs on MGV

4. Supporting information for subgroup analysis

4.1 Subgroup analysis of the effect of TGs on Scr based on species

4.2 Subgroup analysis of the effect of TGs on BG based on species

4.3 Subgroup analysis of the effect of TGs on Scr based on sex

4.4 Subgroup analysis of the effect of TGs on BUN based on sex

4.5 Subgroup analysis of the effect of TGs on KW based on sex

4.6 Subgroup analysis of the effect of TGs on KI based on sex

4.7 Subgroup analysis of the effect of TGs on BG based on sex

4.8 Subgroup analysis of the effect of TGs on ALT based on sex

4.9 Subgroup analysis of the effect of TGs on Scr based on type of DM

4.10 Subgroup analysis of the effect of TGs on BUN based on type of DM

4.11 Subgroup analysis of the effect of TGs on 24h UP based on type of DM

4.12 Subgroup analysis of the effect of TGs on 24h UMA based on type of DM

4.13 Subgroup analysis of the effect of TGs on KW based on type of DM

4.14 Subgroup analysis of the effect of TGs on KI based on type of DM

4.15 Subgroup analysis of the effect of TGs on BG based on type of DM

4.16 Subgroup analysis of the effect of TGs on TC based on type of DM

4.17 Subgroup analysis of the effect of TGs on TG based on type of DM

4.18 Subgroup analysis of the effect of TGs on ALT based on type of DM

4.19 Subgroup analysis of the effect of TGs on MGV based on type of DM

4.20 Subgroup analysis of the effect of TGs on Scr based on number of study arms

4.21 Subgroup analysis of the effect of TGs on BUN based on number of study arms

4.22 Subgroup analysis of the effect of TGs on 24h UP based on number of study arms

4.23 Subgroup analysis of the effect of TGs on 24h UA based on number of study arms

4.24 Subgroup analysis of the effect of TGs on 24h UMA based on number of study arms

4.25 Subgroup analysis of the effect of TGs on KW based on number of study arms

4.26 Subgroup analysis of the effect of TGs on KI based on number of study arms

4.27 Subgroup analysis of the effect of TGs on BG based on number of study arms

4.28 Subgroup analysis of the effect of TGs on TC based on number of study arms

4.29 Subgroup analysis of the effect of TGs on TG based on number of study arms

4.30 Subgroup analysis of the effect of TGs on ALT based on number of study arms

4.31 Subgroup analysis of the effect of TGs on MGV based on number of study arms

4.32 Subgroup analysis of the effect of TGs on Scr based on intervention duration

4.33 Subgroup analysis of the effect of TGs on BUN based on intervention duration

4.34 Subgroup analysis of the effect of TGs on 24h UP based on intervention duration

4.35 Subgroup analysis of the effect of TGs on KI based on intervention duration

4.36 Subgroup analysis of the effect of TGs on BG based on intervention duration

4.37 Subgroup analysis of the effect of TGs on ALT based on intervention duration

5. Supporting information for meta-regression analysis

5.1 Table of results from meta-regression analysis

| Outcomes | Characteristics (covariates) | adjusted *R^2^* | *P* | *I^2^*_res |
| --- | --- | --- | --- | --- |
| Scr | Molding method | 6.14% | 0.1577 | 86.87% |
|  | Strain | -4.35% | 0.6132 | 86.67% |
|  | Manufacturer of TGs | -7.68% | 0.6508 | 87.27% |
|  | STZ dose | 7.48% | 0.114 | 85.20% |
|  | Methodological quality score | 0.94% | 0.253 | 86.63% |
|  | Sample size | -1.16% | 0.276 | 86.52% |
|  | Reference type | 19.79% | **0.005** | 84.25% |
|  | Publication year | 1.21% | 0.260 | 85.79% |
| BUN | Molding method | 1.11% | 0.3884 | 86.53% |
|  | Strain | -7.50% | 0.9845 | 86.66% |
|  | Manufacturer of TGs | -11.65% | 0.9060 | 84.73% |
|  | STZ dose | 15.71% | **0.019** | 84.41% |
|  | Methodological quality score | -0.73% | 0.302 | 86.15% |
|  | Sample size | -4.32% | 0.612 | 86.50% |
|  | Reference type | 27.33% | **0.004** | 83.18% |
|  | Publication year | 3.29% | 0.127 | 85.34% |
| 24h UP | Molding method | -4.35% | 0.0838 | 79.73% |
|  | Strain | 19.48% | **0.0185** | 78.22% |
|  | Manufacturer of TGs | -8.47% | 0.6717 | 80.20% |
|  | STZ dose | -13.64% | 0.509 | 80.22% |
|  | Methodological quality score | -2.86% | 0.176 | 80.26% |
|  | Sample size | -11.67% | 0.961 | 79.77% |
|  | Reference type | 9.38% | 0.154 | 79.09% |
|  | Publication year | -5.19% | 0.253 | 80.19% |
| KW | Molding method | -66.43% | 0.9941 | 64.72% |
|  | Strain | 11.38% | 0.3727 | 48.26% |
|  | Manufacturer of TGs | -75.22% | 0.8993 | 69.30% |
|  | STZ dose | 27.68% | 0.166 | 44.54% |
|  | Methodological quality score | -8.67% | 0.640 | 55.87% |
|  | Sample size | -14.80% | 0.712 | 56.78% |
|  | Reference type | -7.86% | 0.419 | 54.53% |
|  | Publication year | -19.43% | 0.710 | 57.44% |
| KI | Molding method | -7.72% | 0.7046 | 85.12% |
|  | Strain | -5.70% | 0.5851 | 85.84% |
|  | Manufacturer of TGs | -10.07% | 0.5043 | 87.88% |
|  | STZ dose | -5.02% | 0.639 | 85.99% |
|  | Methodological quality score | -4.64% | 0.394 | 86.05% |
|  | Sample size | 1.39% | 0.421 | 85.40% |
|  | Reference type | -3.63% | 0.449 | 86.02% |
|  | Publication year | 11.98% | 0.105 | 84.30% |
| BG | Molding method | 7.86% | 0.1313 | 85.73% |
|  | Strain | -7.05% | 0.9363 | 86.40% |
|  | Manufacturer of TGs | 11.85% | 0.2096 | 85.33% |
|  | STZ dose | -2.82% | 0.657 | 86.15% |
|  | Methodological quality score | -3.53% | 0.962 | 86.22% |
|  | Sample size | -3.29% | 0.805 | 86.02% |
|  | Reference type | 3.84% | 0.112 | 86.05% |
|  | Publication year | 4.77% | 0.154 | 85.36% |
| TC | Molding method | -8.88% | 0.6260 | 80.81% |
|  | Strain | -0.56% | 0.4580 | 80.77% |
|  | Manufacturer of TGs | 13.85% | 0.2972 | 80.14% |
|  | STZ dose | 14.11% | 0.173 | 76.16% |
|  | Methodological quality score | -11.47% | 0.861 | 82.22% |
|  | Sample size | -10.28% | 0.820 | 81.06% |
|  | Reference type | 35.89% | **0.046** | 70.18% |
|  | Publication year | 8.05% | 0.311 | 79.11% |
| TG | Molding method | 35.36% | 0.0520 | 63.41% |
|  | Strain | 6.76% | 0.3414 | 73.09% |
|  | Manufacturer of TGs | 6.68% | 0.3808 | 74.19% |
|  | STZ dose | 38.92% | 0.057 | 61.42% |
|  | Methodological quality score | 25.95% | 0.080 | 67.55% |
|  | Sample size | -8.18% | 0.695 | 74.66% |
|  | Reference type | 70.07% | **0.009** | 44.25% |
|  | Publication year | -8.95% | 0.780 | 74.93% |
| ALT | Molding method | -0.41% | 0.3924 | 73.40% |
|  | Strain | -7.24% | 0.4770 | 75.24% |
|  | Manufacturer of TGs | 43.74% | 0.0823 | 63.82% |
|  | STZ dose | -9.04% | 0.673 | 73.36% |
|  | Methodological quality score | 14.22% | 0.093 | 73.33% |
|  | Sample size | 18.68% | 0.108 | 69.84% |
|  | Reference type | -12.71% | 0.987 | 74.90% |
|  | Publication year | 6.36% | 0.134 | 73.41% |

Note: Bold text indicates statistically significant differences.

6. Supporting information for publication bias

6.1 Funnel plot of the effect of TGs on Scr(A), BUN(B), 24h UP(C), KW(D), KI(E), BG(F), TC(G), TG(H), ALT(I), AST(J) and ALB(K)

6.2 Egger's test plot of the effect of TGs on Scr(A), BUN(B), 24h UP(C), KW(D), KI(E), BG(F), TC(G), TG(H), ALT(I), AST(J) and ALB(K)

6.3 Filled funnel plot of the effect of TGs on Scr(A), BUN(B), 24h UP(C), KI(D), BG(E), ALT(F)

6.4 Table of trim-and-fill analysis results for Scr(A), BUN(B), 24h UP(C), KI(D), BG(E), ALT(F)

| Outcomes | Meta-analysis | | | No. of studies to be filled | Filled Meta-analysis | | |
| --- | --- | --- | --- | --- | --- | --- | --- |
|  | SMD [95% CI] | *P_1_* | *P_2_* |  | SMD [95% CI] | *P_1_* | *P_2_* |
| Scr | -1.89 [-2.30, -1.49] | < 0.001 | < 0.001 | 0 | -1.89 [-2.30, -1.49] | < 0.001 | < 0.001 |
| BUN | -1.96 [-2.36, -1.56] | < 0.001 | < 0.001 | 0 | -1.96 [-2.36, -1.56] | < 0.001 | < 0.001 |
| 24h UP | -3.71 [-4.26, -3.16] | < 0.001 | < 0.001 | 0 | -3.71 [-4.26, -3.16] | < 0.001 | < 0.001 |
| KI | -1.97 [-2.46, -1.47] | < 0.001 | < 0.001 | 5 | -2.43 [-3.01, -1.86] | < 0.001 | < 0.001 |
| BG | -1.22 [-1.60, -0.84] | < 0.001 | < 0.001 | 0 | -1.22 [-1.60, -0.84] | < 0.001 | < 0.001 |
| ALT | -0.14 [-0.58, 0.30] | 0.54 | < 0.001 | 2 | -0.33 [-0.79, 0.13] | 0.16 | < 0.001 |

Note: *P_1_*, *P*-value for overall effect; *P_2_*, *P*-value for heterogeneity.

7. Supporting information for evidence certainty

7.1 Evidence quality grade table of important outcomes

| Outcomes | Risk of bias | Inconsistency | Indirectness | Imprecision | Publication bias | Certainty |
| --- | --- | --- | --- | --- | --- | --- |
| Scr | Serious^a^ | Not serious | Not serious | Not serious | Serious^c^ | Low |
| BUN | Serious^a^ | Not serious | Not serious | Not serious | Serious^c^ | Low |
| 24h UP | Serious^a^ | Not serious | Not serious | Not serious | Serious^c^ | Low |
| 24h UA | Serious^a^ | Serious^b^ | Not serious | Not serious | Not serious | Low |
| 24h UMA | Serious^a^ | Very serious^b^ | Not serious | Not serious | Not serious | Very low |
| KW | Serious^a^ | Serious^b^ | Not serious | Not serious | Not serious | Low |
| KI | Serious^a^ | Not serious | Not serious | Not serious | Serious^c^ | Low |
| BG | Serious^a^ | Not serious | Not serious | Not serious | Serious^c^ | Low |
| TC | Serious^a^ | Not serious | Not serious | Not serious | Not serious | Moderate |
| TG | Serious^a^ | Not serious | Not serious | Not serious | Not serious | Moderate |
| ALT | Serious^a^ | Not serious | Not serious | Not serious | Serious^c^ | Low |
| AST | Serious^a^ | Not serious | Not serious | Not serious | Not serious | Moderate |
| ALB | Serious^a^ | Not serious | Not serious | Not serious | Not serious | Moderate |
| WBC | Serious^a^ | Not serious | Not serious | Not serious | Not serious | Moderate |
| RBC | Serious^a^ | Not serious | Not serious | Not serious | Not serious | Moderate |
| PLT | Serious^a^ | Not serious | Not serious | Not serious | Not serious | Moderate |
| MGA | Serious^a^ | Not serious | Not serious | Not serious | Not serious | Moderate |
| MGV | Serious^a^ | Serious^b^ | Not serious | Not serious | Not serious | Low |

Explanations: a. It is not stated whether allocation concealment and blinding were adopted. b. Heterogeneity is large and cannot be reasonably explained. c. The funnel plot was asymmetric and the P value of the egger's test was less than 0.05.
